# The impact of underweight and obesity on outcomes in anticoagulated patients with atrial fibrillation: A systematic review and meta‐analysis on the obesity paradox

**DOI:** 10.1002/clc.23593

**Published:** 2021-03-26

**Authors:** Maxim Grymonprez, Andreas Capiau, Tine L. De Backer, Stephane Steurbaut, Koen Boussery, Lies Lahousse

**Affiliations:** ^1^ Department of Bioanalysis, Pharmaceutical Care Unit, Faculty of Pharmaceutical Sciences Ghent University Ghent Belgium; ^2^ Department of Cardiology Ghent University Hospital Ghent Belgium; ^3^ Centre for Pharmaceutical Research Research group of Clinical Pharmacology and Clinical Pharmacy, Vrije Universiteit Brussel Jette Belgium; ^4^ Department of Epidemiology Erasmus Medical Center Rotterdam The Netherlands

**Keywords:** anticoagulants, atrial fibrillation, body mass index, meta‐analysis, obesity, underweight

## Abstract

Although obesity is associated with the development and progression of atrial fibrillation (AF), an obesity paradox may be present, illustrated by seemingly protective effects of obesity on AF‐related outcomes. Body mass index (BMI) has an impact on outcomes in AF patients using oral anticoagulants. After searching Medline and Embase, meta‐analysis of results of four randomized and five observational studies demonstrated significantly lower risks of stroke or systemic embolism (RR 0.80, 95%CI [0.73–0.87]; RR 0.63, 95%CI [0.57–0.70]; and RR 0.42, 95%CI [0.31–0.57], respectively) and all‐cause mortality (RR 0.73, 95%CI [0.64–0.83]; RR 0.61, 95%CI [0.52–0.71]; and RR 0.56, 95%CI [0.47–0.66], respectively) in overweight, obese and morbidly obese anticoagulated AF patients (BMI 25 to <30, ≥30 and ≥40 kg/m^2^, respectively) compared to normal BMI anticoagulated AF patients (BMI 18.5 to <25 kg/m^2^). In contrast, thromboembolic (RR 1.92, 95%CI [1.28–2.90]) and mortality (RR 3.57, 95%CI [2.50–5.11]) risks were significantly increased in underweight anticoagulated AF patients (BMI <18.5 kg/m^2^). In overweight and obese anticoagulated AF patients, the risks of major bleeding (RR 0.86, 95%CI [0.76–0.99]; and RR 0.88, 95%CI [0.79–0.98], respectively) and intracranial bleeding (RR 0.75, 95%CI [0.58–0.97]; and RR 0.57, 95%CI [0.40–0.80], respectively) were also significantly lower compared to normal BMI patients, while similar risks were observed in underweight and morbidly obese patients. This meta‐analysis demonstrated lower thromboembolic and mortality risks with increasing BMI. However, as this paradox was driven by results from randomized studies, while observational studies rendered more conflicting results, these seemingly protective effects should still be interpreted with caution.

## 1 INTRODUCTION

Obesity is defined as a body mass index (BMI) of ≥30 kg/m^2^ by the World Health Organization (WHO).^1^ It has been established as an independent risk factor for new‐onset atrial fibrillation (AF) and for the progression from paroxysmal to permanent AF.^2^, ^3^, ^4^ Potential synergistic effects of other obesity‐related AF risk factors have been proposed, such as diabetes mellitus, hypertension, obstructive sleep apnoea, left atrial enlargement, and heart failure with preserved ejection fraction.^2^, ^3^, ^4^ Likewise, underweight (BMI <18.5 kg/m^2^)^1^ has been independently associated with new‐onset AF and AF‐recurrence post‐ablation.^5^, ^6^ A potential U‐shaped relationship between BMI and incident AF has been suggested.^5^ Intriguingly, there seems to be a protective effect of obesity on AF‐related outcomes, despite its association with other cardiovascular diseases, mortality, and stroke risk factors such as diabetes mellitus, metabolic syndrome and hypertension, leading to the controversial concept called the 'obesity paradox.'^7^, ^8^, ^9^, ^10^

Aiming to explore the 'obesity paradox,' this systematic review provides an overview of the literature regarding the impact of extreme BMIs on AF‐related outcomes. A meta‐analysis investigates the impact of underweight, overweight (BMI 25 to <30 kg/m^2^),^1^ obesity, and morbid obesity (BMI ≥40 kg/m^2^)^1^ compared to normal BMI on AF‐related outcomes in anticoagulated AF patients.

## 2 METHODS

An extensive literature search was performed using the Medline and Embase databases (see supplemental materials, eTable 1) by two independent reviewers (M. G. and A. C.). Discrepancies were resolved by a consensus meeting with a senior researcher (L. L.). Longitudinal studies investigating the impact of underweight (BMI <18.5 kg/m^2^),^1^ overweight (BMI 25 to <30 kg/m^2^),^1^ obesity (BMI ≥30 kg/m^2^),^1^ Class II obesity (BMI 35 to <40 kg/m^2^),^1^ and morbid/Class III obesity (BMI ≥40 kg/m^2^)^1^ on clinical outcomes in adult patients with non‐valvular AF compared to normal BMI AF patients (BMI 18.5 to <25 kg/m^2^)^1^ during a mean/median follow‐up of at least 6 months were included. Studies investigating outcomes in AF patients with low body weight (≤50–60 kg) compared to normal weight AF patients were also included and discussed in the supplemental materials, but were not considered for the meta‐analysis. Studies investigating AF subjects undergoing interventions (e.g., cardioversion, ablation) were excluded, given the associated thromboembolic risk. Outcomes of interest were stroke or systemic embolism (stroke/SE), all‐cause mortality and major bleeding (overall, intracranial and/or gastrointestinal). Phase III randomized controlled trials (RCTs) (original trial or secondary analyses), longitudinal observational cohort studies and meta‐analyses were included for the systematic review, whereas case reports, cross‐sectional studies, conference proceedings, reviews or editorials were not considered. No restriction on publication date or language was used.

For the meta‐analysis, results from Phase III RCTs (original trial or secondary analyses) and longitudinal observational cohort studies examining the risk of stroke/SE, all‐cause mortality, major bleeding and intracranial bleeding in underweight, overweight, obese and Class II–III obese AF patients using oral anticoagulants (namely vitamin K antagonists [VKAs] or non‐vitamin K antagonist oral anticoagulants [NOACs]) compared to normal BMI anticoagulated AF patients were selected, with the BMI subgroups categorized according to the WHO BMI classification.^1^ If studies included non‐anticoagulated AF patients, results were excluded from the meta‐analysis, given the significantly lower thromboembolic but potentially higher bleeding risks of anticoagulated AF patients compared to non‐anticoagulated patients, which may influence results independent from BMI.^11^ However, these results were included as a sensitivity analysis.

**FIGURE 1:**
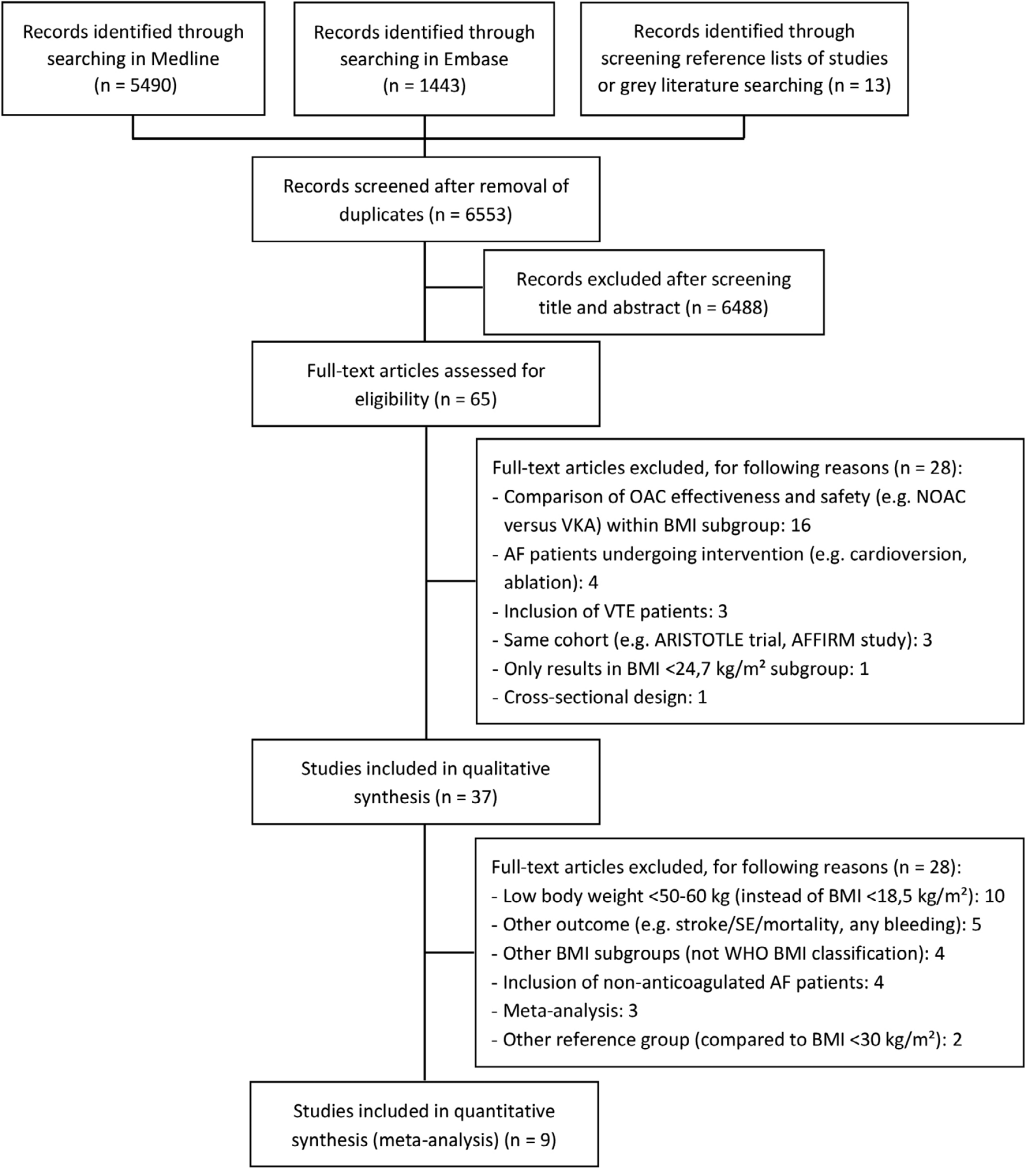
PRISMA flow diagram. AF: atrial fibrillation; BMI: body mass index; NOAC: non‐vitamin K antagonist oral anticoagulant; OAC: oral anticoagulant; PRISMA, preferred reporting items for systematic reviews and meta‐analyses; SE: systemic embolism; VKA: vitamin K antagonist; VTE: venous thromboembolism; WHO: World Health Organization

Up to February 1, 2021, 6553 articles were identified. Additional articles of interest were selected by screening the reference list of studies. If secondary analyses of Phase III RCTs did not report outcome data in specific BMI subgroups (only the case for the RE‐LY trial), the FDA (U.S. Food and Drug Administration) Advisory Committee briefing documents on regulatory submissions for drug approval of NOACs by the pharmaceutical company (e.g., Boehringer Ingelheim) were searched for the gray literature.^12^ After screening title and abstract, 65 articles were selected. After reading the full‐text, 37 articles were selected for the systematic review, of which nine were used for the meta‐analysis (four Phase III RCTs, five observational studies) (Figure 1). An overview of the included studies with study design, patient characteristics and outcome measures is displayed in eTable 2.

The meta‐analysis was performed using a random effects model with the Mantel–Haenszel method. Data of the study methodology (setting, design and duration), patient characteristics (total number and age), comparison (e.g., obesity versus normal BMI), and the aforementioned outcomes of interest were extracted from the original publications, supplemental materials or documents from regulatory submissions for FDA approval. If the number of events was not reported, this was calculated based on the event rate and/or risk estimate. The effect measures of each included study were calculated and reported as the risk ratio (RR) with 95% confidence interval (CI), visually presented in forest plots. A two‐sided p‐value of <.05 was considered statistically significant. Heterogeneity was tested using the I^2^‐statistic. The risk of bias of studies included in the meta‐analysis was assessed using the quality assessment tool 'QUALSYST' from the “Standard Quality Assessment Criteria for Evaluating Primary Research Papers from a Variety of Fields” (eTable 3).^13^ Fourteen items of each study were scored on the study quality and outcome levels depending on the degree to which the specific criteria were met or reported (“yes” = 2, “partial” = 1, “no” = 0, “n/a” if not applicable). For each study, a percentage was calculated by dividing the total score obtained across rated items by the total possible score. Studies were included if scoring ≥75% on the quality assessment tool. Furthermore, the risk of publication bias at the outcome level was evaluated through funnel plot asymmetry. Analyses were performed with Review Manager (RevMan) version 5.3 (The Nordic Cochrane Centre, The Cochrane Collaboration, Copenhagen, 2014) and R (R version 3.6.1 with RStudio version 1.2.5001). This work has been performed according to the preferred reporting items for systematic reviews and meta‐analyses (PRISMA) guidelines (PRISMA checklist included in supplemental materials, eTable 4).

## 3 RESULTS

### 3.1 Systematic review

#### 3.1.1 Thromboembolism

In RCTs investigating oral anticoagulants in AF patients, lower thromboembolic risks have been observed with increasing BMI, corroborating the 'obesity paradox' (eTable 2). Indeed, lower thromboembolic rates were observed in obese versus normal BMI AF patients in the ROCKET AF,^7^ ARISTOTLE,^8^ ENGAGE AF‐TIMI 48,^9^ RE‐LY,^12^ and AMADEUS trial (which investigated the unapproved factor Xa inhibitor idraparinux),^14^ whereas higher stroke/SE risks were observed in underweight AF patients (BMI <18.5 kg/m^2^) included in the ENGAGE AF‐TIMI 48 trial^9^ (not reported in other RCTs). After pooling these results, the meta‐analyses of Proietti et al. (based on three Phase III RCTs)^10^ and Zhou et al. (based on five RCTs including the SPORTIF trial, which investigated the unapproved direct thrombin inhibitor Ximelagatran)^15^ demonstrated a lower stroke/SE risk in (morbidly) obese versus normal BMI AF patients. Likewise, the Korean observational cohort study by Lee et al.^16^ illustrated that obese AF patients were associated with significantly lower ischemic stroke risks compared to normal BMI patients, while underweight was identified as an independent predictor of ischemic stroke^17^ and stroke/SE/mortality^18^ in five Japanese AF registries (64% VKA‐treated, 10% NOAC‐treated)^17^ and the Fushimi AF registry (investigating non‐anticoagulated subjects).^18^

However, in other observational studies, the impact of BMI on AF‐related outcomes was not or less clear, illustrating the general controversy regarding the topic. For example, obesity was associated with similar stroke,^19^ stroke/SE,^4^, ^20^, ^21^, ^22^, ^23^ stroke/SE/myocardial infarction,^24^ or stroke/SE/venous thromboembolism^25^, ^26^ risks compared to normal BMI AF patients included in the FANTASIIA registry^19^; ORBIT‐AF registry (69%–75% VKA‐treated)^4^; J‐RHYTHM registry (86%–91% VKA‐treated)^20^; Danish Diet, Cancer and Health study (19%–24% VKA‐treated)^21^; XAPASS study^24^; PREFER in AF (PROLONGATION) registries^26^; the Korean retrospective cohort study by Park et al.^22^; and two U.S. retrospective cohort studies by Kaplan et al.^23^ and Netley et al.^25^ Similarly, no significant differences in the risk of ischemic stroke,^16^ stroke/SE,^20^, ^22^ or stroke/SE/myocardial infarction^24^ could be demonstrated in underweight versus normal BMI AF patients in two Korean studies by Lee et al.^16^ and Park et al.,^22^ the J‐RHYTHM registry^20^ and the XAPASS study.^24^

In contrast, two observational studies illustrated worse outcomes in obese AF patients. A Croatian cohort study by Lucijanic et al. observed a significantly shorter time to stroke/SE in obese versus non‐obese AF patients.^27^ Likewise, in a Chinese cohort study by Wang et al., the risk of stroke/SE/myocardial infarction was 9% higher per 1 kg/m^2^ increase in BMI, although only 19% were OAC‐treated and analyses were not adjusted for confounders.^28^

#### 3.1.2 Mortality

In line with the impact on the thromboembolic risk, (morbid) obesity was associated with significantly lower all‐cause mortality risks compared to the normal BMI subgroup in the ARISTOTLE^8^ and ENGAGE AF‐TIMI 48 trial,^9^ whereas significantly higher mortality risks were demonstrated in underweight AF patients^9^ (eTable 2). Likewise, significantly lower mortality risks^4^, ^16^, ^20^, ^29^, ^30^ in overweight or obese and significantly higher risks^16^, ^20^, ^22^, ^24^, ^30^ in underweight AF patients were observed in most observational studies. However, in some observational studies, no impact of increasing BMI on the mortality risk was observed.^19^, ^22^, ^24^ Moreover, significantly higher mortality risks were observed in obese versus normal BMI AF patients in the Danish Diet, Cancer and Health study, although it should be noted that only a quarter of patients was anticoagulated at baseline, BMI was measured at the time of study entry (whereas follow‐up started on the date of incident AF) and analyses were only adjusted for the CHA_2_DS_2_‐VASc score.^21^

#### 3.1.3 Major bleeding

As opposed to the lower thromboembolic and mortality risks with increasing BMI, the impact on bleeding outcomes is less evident (eTable 2). Similar major bleeding risks were observed in (morbidly) obese versus normal BMI patients in the ROCKET AF,^7^ ARISTOTLE^8^ and ENGAGE AF‐TIMI 48 trial,^9^ as well as in underweight AF patients.^9^ After pooling results, a significantly lower odds of major bleeding in obese versus normal BMI AF patients was observed in the meta‐analysis of Proietti et al.,^10^ while this was not the case in the meta‐analysis of Zhou et al.^15^ Similar conflicting results were also present in observational studies, with no impact of (morbid) obesity^4^, ^16^, ^19^, ^20^, ^22^, ^23^, ^24^, ^25^, ^26^ or underweight^16^, ^20^, ^24^ on bleeding outcomes observed in most studies. However, the risk of major bleeding was significantly lower per 1 and 5 kg/m^2^ increase in BMI in a Taiwanese^31^ and Korean^16^ study, respectively.

Conversely, significantly higher bleeding risks in obese versus normal BMI AF patients were observed in the Chinese MISSION‐AF study,^32^ as well as a significantly shorter time to major bleeding in the Croatian study by Lucijanic et al.^27^ Similarly, underweight was identified as an independent predictor of bleeding in AF patients included in the Korean study by Park et al.,^22^ as well as in AF patients ≥80 years old included in the Japanese cohort study by Shinohara et al.^33^

In AF patients with low body weight (≤50–60 kg) compared to normal weight, worse thromboembolic and mortality outcomes have also been observed, while bleeding risks were mostly comparable (see additional systematic review in supplemental materials).^34^

### 3.2 Meta‐analysis

Results on AF‐related outcomes in anticoagulated AF patients categorized according to their BMI from four (post hoc analyses of) Phase III RCTs^7^, ^8^, ^9^, ^12^ and five longitudinal observational cohort studies^16^, ^19^, ^22^, ^23^, ^24^ were pooled in a meta‐analysis. However, as only one study^16^ provided data on the gastrointestinal bleeding risk, this outcome could not be included in the meta‐analysis.

**FIGURE 2:**
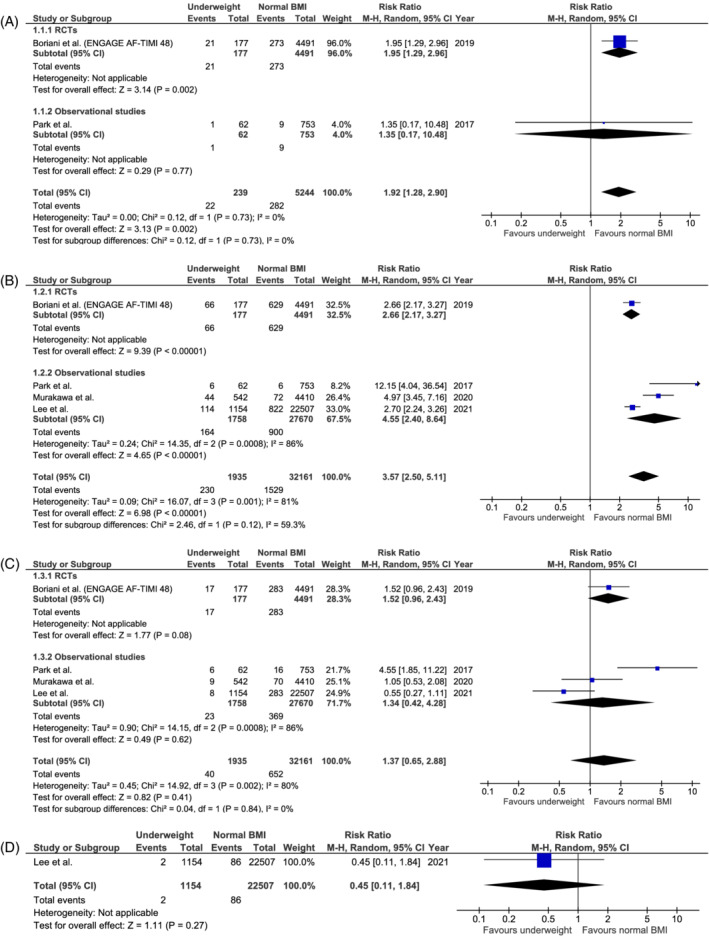
Forest plot of the risk of (A) stroke or systemic embolism, (B) all‐cause mortality, (C) major bleeding, and (D) intracranial bleeding for underweight (BMI <18.5 kg/m^2^) versus normal BMI (18.5 to <25 kg/m^2^) AF patients receiving anticoagulation, categorized according to randomized and observational studies. AF: atrial fibrillation; BMI: body mass index; CI: confidence interval; ENGAGE AF‐TIMI 48: the effective anticoagulation with factor Xa next generation in atrial fibrillation–thrombolysis in myocardial infarction 48 trial; M–H: Mantel–Haenszel (statistical method); RCT: randomized controlled trial

**FIGURE 3:**
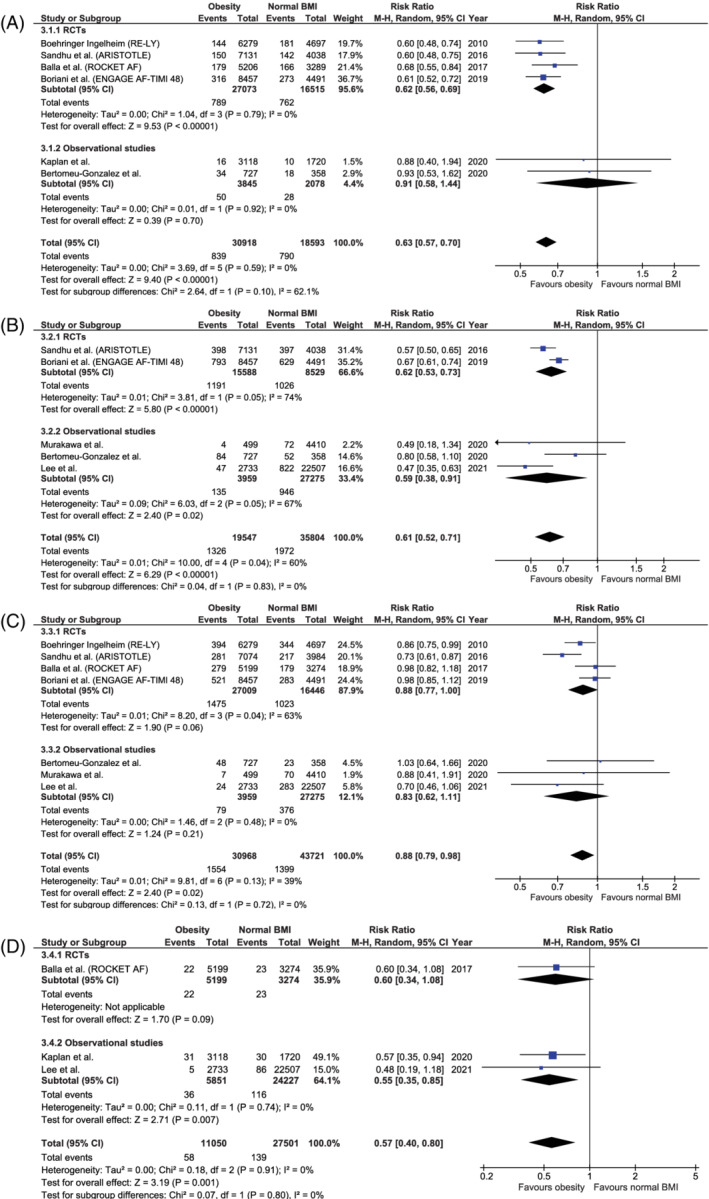
Forest plot of the risk of (A) stroke or systemic embolism, (B) all‐cause mortality, (C) major bleeding, and (D) intracranial bleeding for obese (BMI ≥30 kg/m^2^) versus normal BMI (18.5 to < 25 kg/m^2^) AF patients receiving anticoagulation, categorized according to randomized and observational studies. AF: atrial fibrillation; ARISTOTLE: the apixaban for reduction in stroke and other thromboembolic events in atrial fibrillation trial; BMI: body mass index; CI: confidence interval; ENGAGE AF‐TIMI 48: the effective anticoagulation with factor Xa next generation in atrial fibrillation–thrombolysis in myocardial infarction 48 trial; M–H: Mantel–Haenszel (statistical method); RCT: randomized controlled trial; RE‐LY: the randomized evaluation of long‐term anticoagulation therapy; ROCKET AF: the rivaroxaban once daily oral direct factor Xa inhibition compared with vitamin k antagonism for prevention of stroke and embolism trial in atrial fibrillation

Compared to normal BMI (18.5 to <25 kg/m^2^) anticoagulated AF patients, the risk of stroke/SE was significantly higher in underweight (BMI <18.5 kg/m^2^) anticoagulated AF patients (RR 1.92, 95%CI [1.28–2.90], p‐value .002), whereas significantly lower risks were seen in overweight (BMI 25 to <30 kg/m²), obese (BMI ≥30 kg/m^2^) and morbidly obese (BMI ≥40 kg/m^2^) anticoagulated AF patients (RR 0.80, 95%CI [0.73–0.87], p‐value <.001; RR 0.63, 95%CI [0.57–0.70], p‐value <.001; and RR 0.42, 95%CI [0.31–0.57], p‐value <.001, respectively) (Figures 2(A), 3(A), eFigure 1A‐3A).

Likewise, the risk of all‐cause mortality was significantly higher in underweight versus normal BMI anticoagulated AF patients (RR 3.57, 95%CI [2.50–5.11], p‐value <.001), while significantly lower risks were demonstrated in overweight, obese and morbidly obese anticoagulated AF patients (RR 0.73, 95%CI [0.64–0.83], p‐value <.001; RR 0.61, 95%CI [0.52–0.71], p‐value <.001; RR 0.56, 95%CI [0.47–0.66], p‐value <.001, respectively) (Figures 2(B),3(B), eFigure 1B‐3B).

Moreover, overweight and obese anticoagulated AF patients were associated with significantly lower major bleeding (RR 0.86, 95%CI [0.76–0.99], p‐value .03; and RR 0.88, 95%CI [0.79–0.98], p‐value .02, respectively) and intracranial bleeding risks (RR 0.75, 95%CI [0.58–0.97], p‐value .03; and RR 0.57, 95%CI [0.40–0.80], p‐value .001, respectively) compared to normal BMI AF patients, whereas similar major and intracranial bleeding risks were observed in underweight (RR 1.37, 95%CI [0.65–2.88], p‐value .41; and RR 0.45, 95%CI [0.11–1.84], p‐value .27, respectively) and morbidly obese anticoagulated AF patients (RR 0.73, 95%CI [0.38–1.42], p‐value .36; no data on intracranial bleeding risk) (Figures 2(C), 3(C) and 2(D), 3(D), eFigure 1C‐3C and 1D).

Similar trends were observed in anticoagulated AF patients with Class II obesity (BMI 35‐ < 40 kg/m^2^) (eFigure 2). Moreover, additionally including data from four studies^4^, ^20^, ^21^, ^29^ with non‐anticoagulated AF patients as a sensitivity analysis rendered consistent results (eFigure 4).

No publication bias was suspected based on visual inspection of the funnel plots, although the interpretation may not have been reliable, as less than 10 studies were included in the meta‐analysis (eFigure 7). All included studies scored ≥75% on the quality assessment tool 'QUALSYST'^13^ (eTable 3). For most outcomes, no substantial heterogeneity was detected. However, regarding the risk of mortality in overweight (I^2^ 62%) and Class II obese AF patients (I^2^ 69%), and the risk of major bleeding in overweight (I^2^ 64%) and morbidly obese AF patients (I^2^ 88%), substantial heterogeneity was detected, probably caused by heterogeneous results from the included randomized studies. Indeed, overweight and Class II obese patients included in the ARISTOTLE trial^8^ had lower mortality risks than their peers included in the ENGAGE AF‐TIMI 48 trial,^9^ which is likely the result of the inclusion of older overweight and Class II obese patients in the latter trial (e.g., median age of overweight AF patients in the ENGAGE AF‐TIMI 48 trial^9^ was 73 years (67–79), whereas the mean age of overweight AF patients in the ARISTOTLE trial^8^ was 70.1 years +/− 9.3). Regarding the heterogeneous results of major bleeding in morbidly obese AF patients, the major bleeding risk was higher in morbidly obese AF patients included in the ENGAGE AF‐TIMI 48 trial^9^ than in the ARISTOTLE trial,^8^ despite good INR control in warfarin‐treated patients and no significant difference in the pharmacokinetics and ‐dynamics of edoxaban compared to apixaban. Also, the use of antiplatelets cannot (fully) explain these heterogeneous safety results, as 33.2% of morbidly obese patients in the ENGAGE AF‐TIMI 48 trial^9^ and 33.1% of obese patients in the ARISTOTLE trial^8^ used antiplatelets. Lastly, substantial heterogeneity in the risks of mortality and major bleeding was detected in underweight AF patients (I^2^ 81% and 80%, respectively), probably due to heterogeneous results of the included observational studies.^16^, ^22^, ^24^ Indeed, after one‐by‐one exclusion of these studies, results remained the same, but heterogeneity was generally lower (eFigure 5,6).

## 4 DISCUSSION

**FIGURE 4:**
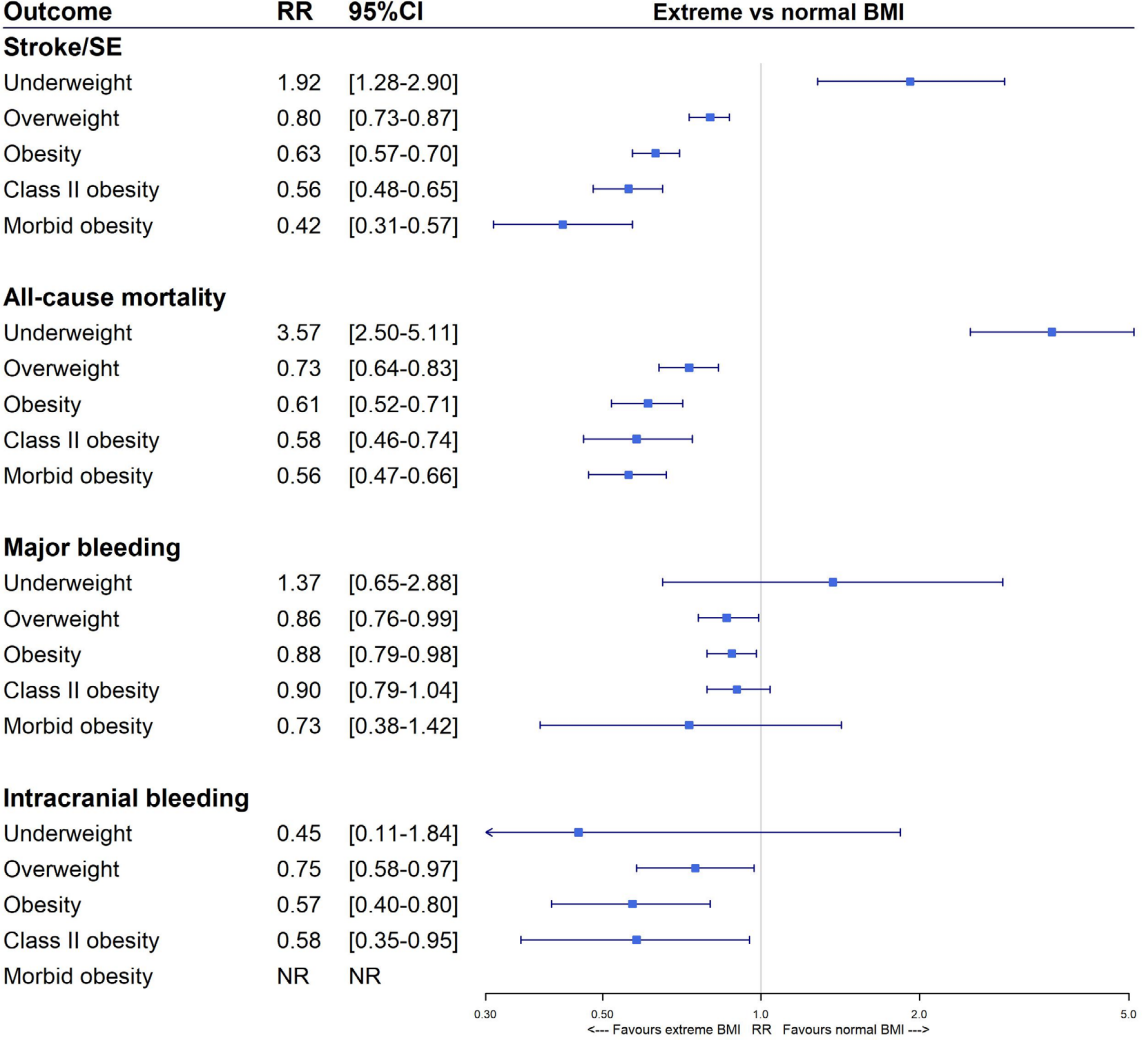
Overview of the meta‐analyzed risk estimates of stroke or systemic embolism, mortality, major bleeding and intracranial bleeding for underweight (BMI <18.5 kg/m^2^), overweight (25 to < 30 kg/m^2^), obese (≥30 kg/m^2^), Class II obese (35 to <40 kg/m²) and morbidly obese (≥40 kg/m^2^) versus normal BMI (18.5 to < 25 kg/m^2^) AF patients receiving anticoagulation, respectively. AF: atrial fibrillation; BMI: body mass index; CI: confidence interval; NR: not reported; RR: risk ratio; SE: systemic embolism

As a vivid debate is still ongoing whether or not (morbid) obesity has a protective effect on AF‐related outcomes, this meta‐analysis based on four randomized^7^, ^8^, ^9^, ^12^ and five observational^16^, ^19^, ^22^, ^23^, ^24^ studies explored the controversial 'obesity paradox' concept (Figure 4). In line with results from the meta‐analyses of Proietti et al.^10^ and Zhou et al.,^15^ we demonstrated lower stroke/SE and mortality risks with increasing BMI, corroborating the 'obesity paradox.' On the contrary, underweight (BMI <18.5 kg/m^2^) was associated with higher thromboembolic and mortality risks, which may be suggestive of a 'lean paradox.' This is in line with results in AF patients with low body weight of ≤60 kg compared to normal weight, illustrated by the meta‐analysis by Boonyawat et al.^34^ Moreover, the impact of BMI on major and intracranial bleeding risks was less evident, although significantly lower bleeding risks were observed in overweight and obese patients with AF compared to those with a normal BMI. Intriguingly, these trends appeared to be mostly driven by results from randomized studies, while subsequent observational studies rendered more conflicting results. However, these findings should not justify maintaining or neglecting a high BMI in AF patients. Clinicians should still direct their efforts on advocating weight control and on intensively tackling other cardiovascular risk factors in (morbidly) obese AF patients, as recommended by guidelines.^9^, ^35^

### 4.1 Hypotheses on the 'obesity paradox'

Several considerations and hypotheses have been suggested in order to elucidate the apparent protective effect of obesity on AF‐related outcomes. First, cardiovascular risk factors (e.g., hypertension, dyslipidaemia and diabetes) may have been tackled earlier in obese patients, resulting in faster and more intensive medical treatment.^8^ For example, in the ARISTOTLE trial, 50%, 68%, and 77% of obese AF patients were treated with statins, beta‐blockers and ACE‐inhibitors, compared to 34%, 56%, and 61% of normal BMI patients, respectively.^8^

Second, the large metabolic reserves present in obese patients may help to cope with chronic diseases and help to survive complications or exacerbations.^4^, ^19^ Conversely, poor nutritional status in frail underweight patients may increase their susceptibility to adverse outcomes such as hospitalizations and mortality.^36^

Third, inflammation (mediated by interleukin‐6, IL‐6) and activation of the renin‐angiotensin‐aldosterone system (expression of angiotensin‐II receptors which activate thromboxane A2 and induce IL‐6) promote a prothrombotic state in AF.^7^, ^37^, ^38^ Obesity, independent from AF, has also been linked to systemic inflammation (elevated levels of C‐reactive protein, IL‐6 and tumor necrosis factor‐α [TNF‐α]), due to IL‐6 and TNF‐α production from (visceral) adipose tissue.^39^, ^40^ In underweight and frail patients, an increase in systemic inflammation and higher levels of renin in response to stress have also been described, potentially leading to more adverse outcomes.^41^, ^42^ On the contrary, other studies have suggested that a decreased renin‐angiotensin response to stress and more production of soluble TNF‐α receptors in adipose tissue of obese patients may reduce inflammation and potentially result in a lower prothrombotic state.^7^, ^8^, ^42^, ^43^ Overall, whether or not differences in systemic inflammation may influence thromboembolic outcomes in obese AF patients remains questioned.

Fourth, selection bias in randomized studies may have influenced results, illustrated by the difference in comorbidities and age between obese and normal BMI AF patients. Indeed, obese patients included in RCTs frequently had a better renal function and less prior stroke/SE, although the prevalence of hypertension and diabetes mellitus was higher.^7^, ^8^ Moreover, obese patients tend to develop cardiovascular diseases such as AF earlier than normal BMI patients^2^ and have therefore a greater proportion of life lived with cardiovascular morbidity.^7^, ^9^ Indeed, obese AF patients in randomized studies were considerably younger than normal BMI patients (e.g., median age of normal BMI, obese and morbidly obese AF patients included in the ENGAGE AF‐TIMI 48 trial was 75, 71, and 64 years, respectively),^9^ which may have resulted in lower all‐cause mortality and thromboembolic risks in these younger obese AF patients. Similarly, underweight AF patients tended to be older than normal BMI patients (e.g., mean age of 78 and 74 years, respectively, in the XAPASS trial^24^). Even though age‐adjusted analyses were performed in most studies, residual confounding due to age‐related cardiovascular deterioration (e.g., systemic atherosclerosis) and other underlying age‐related mechanisms may explain this 'obesity paradox.' Intriguingly, in two observational studies that included normal BMI and obese AF patients of comparable age, a higher mortality^21^ and bleeding^32^ risk were documented in obese versus normal BMI AF patients. Therefore, the 'obesity paradox' may be less or not observed in observational studies, possibly due to the inclusion of older, more comorbid obese AF patients with potential off‐label NOAC dosing and suboptimal adherence, than those included in randomized studies.

Lastly, in line with the fourth hypothesis, the observed worse outcomes in underweight versus normal BMI AF patients may have been the result of older age and a higher comorbidity burden, as underweight has been associated with frailty and chronic diseases in the elderly.^16^, ^33^ Malnutrition and underlying conditions such as malignancies and COPD, may play a role in the increased risk of adverse outcomes, especially mortality.^16^, ^33^

### 4.2 Strengths and limitations

Our systematic review and meta‐analysis have several strengths, such as the inclusion of both Phase III RCTs, characterized by detailed methodologies and well‐defined cohorts, and longitudinal observational cohort studies, which include large real‐world patient subgroups with long follow‐up. By pooling results, we have included large numbers of patients for each outcome, even in the subgroup of patients with underweight and morbid obesity, who were underrepresented in randomized studies. Moreover, we have only included patients based on the BMI, as a body weight of <60 kg or >120 kg does not necessarily correspond with underweight or morbid obesity, respectively (e.g., any person larger than 1.73 meters with a body weight of 120 kg has a BMI of <40 kg/m^2^).

Several limitations should be mentioned complicating the comparability of included studies. First, classification of patients according to BMI differed between studies. For example, some studies categorized their patient cohorts according to the BMI tertiles^31^, ^32^ or quartiles,^26^ or did not use the WHO BMI classification.^30^ Second, BMI was usually measured at baseline, not adjusting for weight changes during follow‐up. However, in the ARISTOTLE trial, only very small weight changes were noted during follow‐up.^44^ Third, four studies^4^, ^20^, ^21^, ^29^ included non‐anticoagulated AF patients, resulting in the exclusion of their results in the meta‐analysis to overcome this shortcoming. However, results were consistent in a sensitivity analysis with inclusion of these studies. Fourth, NOAC dosages varied between studies, as rivaroxaban 15 and 10 mg once daily are the approved standard and reduced dosages in Japan^45^ (as opposed to 20 and 15 mg in Europe)^35^ and dabigatran 75 mg twice daily is the approved reduced dosage in the U.S.^12^ (compared to 110 mg twice daily in Europe).^35^ Fifth, endpoints frequently differed from our outcomes of interest, as some studies^14^, ^16^, ^17^, ^18^, ^24^, ^28^, ^32^, ^33^ examined the risk of ischemic stroke, stroke/SE/mortality, stroke/SE/myocardial infarction, or any (major or minor) bleeding. Lastly, results from observational studies^16^, ^17^, ^18^, ^20^, ^22^, ^24^, ^30^, ^33^ on AF‐related outcomes in underweight versus normal BMI AF patients were all performed in an Asian setting potentially limiting generalizability. Similarly, morbidly obese AF patients were more likely to be of Caucasian ethnicity (especially from North America).^8^, ^9^ These results should not be automatically extrapolated to other populations due to potential ethnic differences, as VKA‐treated Asian AF patients tend to have more major bleeding events (especially intracranial bleeding), higher stroke rates (especially haemorrhagic stroke) and a lower mean time in therapeutic range than VKA‐treated Caucasian AF patients.^46^, ^47^

## 5 CONCLUSION

In conclusion, this meta‐analysis exploring the controversial 'obesity paradox' demonstrated lower thromboembolic and mortality risks with increasing BMI in anticoagulated AF patients. However, as this paradox was driven by results from randomized studies, while subsequent observational studies rendered more conflicting results, these seemingly protective effects should still be interpreted with caution.

## AUTHOR CONTRIBUTIONS

Maxim Grymonprez and Lies Lahousse contributed to the concept and design of the systematic review. Maxim Grymonprez and Andreas Capiau performed the literature search. Maxim Grymonprez performed the statistical analysis, interpretation and writing. Andreas Capiau, Tine L. De Backer, Stephane Steurbaut, Koen Boussery, and Lies Lahousse revised the systematic review critically. All authors contributed to the article and approved the submitted version.

## Supporting information


**Appendix S1**: Supporting information

## Data Availability

The data underlying this article are available in the article and in its online supplemental materials.

## References

[1] World Health Organization (WHO) Body mass index (BMI) classification. http://www.euro.who.int/en/health-topics/disease-prevention/nutrition/a-healthy-lifestyle/body-mass-index-bmi. 2020.

[2] Wanahita N , Messerli FH , Bangalore S , Gami AS , Somers VK , Steinberg JS . Atrial fibrillation and obesity‐results of a meta‐analysis. Am Heart J. 2008;155(2):310‐315. 18215602 10.1016/j.ahj.2007.10.004

[3] Tsang TS , Barnes ME , Miyasaka Y , et al. Obesity as a risk factor for the progression of paroxysmal to permanent atrial fibrillation: a longitudinal cohort study of 21 years. Eur Heart J. 2008;29(18):2227‐2233. 18611964 10.1093/eurheartj/ehn324 PMC2733739

[4] Pandey A , Gersh BJ , McGuire DK , et al. Association of Body Mass Index with care and outcomes in patients with atrial fibrillation: results from the ORBIT‐AF registry. J Am Coll Cardiol EP. 2016;2(3):355‐363. 10.1016/j.jacep.2015.12.001 29766895

[5] Kang SH , Choi EK , Han KD , et al. Underweight is a risk factor for atrial fibrillation: a nationwide population‐based study. Int J Cardiol. 2016;215:449‐456. 27131763 10.1016/j.ijcard.2016.04.036

[6] Deng H , Shantsila A , Guo P , et al. A U‐shaped relationship of body mass index on atrial fibrillation recurrence post ablation: a report from the Guangzhou atrial fibrillation ablation registry. EBioMedicine. 2018;35:40‐45. 30174278 10.1016/j.ebiom.2018.08.034 PMC6156736

[7] Balla SR , Cyr DD , Lokhnygina Y , et al. Relation of risk of stroke in patients with atrial fibrillation to body mass index (from patients treated with rivaroxaban and warfarin in the rivaroxaban once daily Oral direct factor Xa inhibition compared with vitamin K antagonism for prevention of stroke and embolism trial in atrial fibrillation trial). Am J Cardiol. 2017;119(12):1989‐1996. 28477860 10.1016/j.amjcard.2017.03.028

[8] Sandhu RK , Ezekowitz J , Andersson U , et al. The 'obesity paradox' in atrial fibrillation: observations from the ARISTOTLE (Apixaban for reduction in stroke and other thromboembolic events in atrial fibrillation) trial. Eur Heart J. 2016;37(38):2869‐2878. 27071819 10.1093/eurheartj/ehw124

[9] Boriani G , Ruff CT , Kuder JF , et al. Relationship between body mass index and outcomes in patients with atrial fibrillation treated with edoxaban or warfarin in the ENGAGE AF‐TIMI 48 trial. Eur Heart J. 2019;40(19):1541‐1550. 30624719 10.1093/eurheartj/ehy861

[10] Proietti M , Guiducci E , Cheli P , Lip GY . Is there an obesity paradox for outcomes in atrial fibrillation? A systematic review and meta‐analysis of non‐vitamin K antagonist Oral anticoagulant trials. Stroke. 2017;48(4):857‐866. 28265017 10.1161/STROKEAHA.116.015984

[11] Hart RG , Pearce LA , Aguilar MI . Meta‐analysis: antithrombotic therapy to prevent stroke in patients who have nonvalvular atrial fibrillation. Ann Intern Med. 2007;146(12):857‐867. 17577005 10.7326/0003-4819-146-12-200706190-00007

[12] Boehringer Ingelheim Pharmaceuticals . FDA Advisory Committee Briefing Document, Dabigatran Etexilate Mesylate Capsules, for the September 20, 2010 Meeting of the Cardiovascular and Renal Drugs Advisory Committee. https://wayback.archive‐it.org/7993/20170405212218/https://www.fda.gov/downloads/AdvisoryCommittees/CommitteesMeetingMaterials/Drugs/CardiovascularandRenalDrugsAdvisoryCommittee/UCM247244.pdf.

[13] Kmet L , Lee R , Cook L . The Quality Assessment Tool ‘QUALSYST’ from the “Standard Quality Assessment Criteria for Evaluating Primary Research Papers from a Variety of Fields”. 2004. https://www.ihe.ca/advanced‐search/standard‐quality‐assessment‐criteria‐for‐evaluating‐primary‐research‐papers‐from‐a‐variety‐of‐fields.

[14] Senoo K , Lip GY . Body mass index and adverse outcomes in elderly patients with atrial fibrillation: the AMADEUS trial. Stroke. 2016;47(2):523‐526. 26628383 10.1161/STROKEAHA.115.011876

[15] Zhou Y , Ma J , Zhu W . Efficacy and safety of direct Oral anticoagulants versus warfarin in patients with atrial fibrillation across BMI categories: a systematic review and meta‐analysis. Am J Cardiovasc Drugs. 2020;20(1):51‐60. 31342343 10.1007/s40256-019-00362-4

[16] Lee SR , Choi EK , Jung JH , et al. Body mass index and clinical outcomes in Asian patients with atrial fibrillation receiving oral anticoagulation. Stroke. 2021;52(2):521‐530. 33423512 10.1161/STROKEAHA.120.030356

[17] Okumura K , Tomita H , Nakai M , et al. Risk factors associated with ischemic stroke in Japanese patients with Nonvalvular atrial fibrillation. JAMA Netw Open. 2020;3(4):e202881. 32293685 10.1001/jamanetworkopen.2020.2881 PMC7160687

[18] Hamatani Y , Yamashita Y , Esato M , et al. Predictors for stroke and death in non‐Anticoagulated Asian patients with atrial fibrillation: the Fushimi AF registry. PloS One. 2015;10(11):e0142394. 26540107 10.1371/journal.pone.0142394 PMC4634924

[19] Bertomeu‐Gonzalez V , Moreno‐Arribas J , Esteve‐Pastor MA , et al. Association of body mass index with clinical outcomes in patients with atrial fibrillation: a report from the FANTASIIA registry. J Am Heart Assoc. 2020;9(1):e013789. 31870235 10.1161/JAHA.119.013789 PMC6988150

[20] Inoue H , Kodani E , Atarashi H , et al. Impact of body mass index on the prognosis of Japanese patients with non‐Valvular atrial fibrillation. Am J Cardiol. 2016;118(2):215‐221. 27255662 10.1016/j.amjcard.2016.04.036

[21] Overvad TF , Rasmussen LH , Skjøth F , et al. Body mass index and adverse events in patients with incident atrial fibrillation. Am J Med. 2013;126(7):640.e9‐e6.4E17. 10.1016/j.amjmed.2012.11.024 23601271

[22] Park CS , Choi EK , Kim HM , Lee SR , Cha MJ , Oh S . Increased risk of major bleeding in underweight patients with atrial fibrillation who were prescribed non‐vitamin K antagonist oral anticoagulants. Heart Rhythm. 2017;14(4):501‐507. 28042092 10.1016/j.hrthm.2016.12.036

[23] Kaplan RM , Tanaka Y , Passman RS , et al. Efficacy and safety of direct Oral anticoagulants for atrial fibrillation across body mass index categories. J Am Heart Assoc. 2020;9(24):e017383. 33302751 10.1161/JAHA.120.017383 PMC7955357

[24] Murakawa Y , Ikeda T , Ogawa S , et al. Impact of body mass index on real‐world outcomes of rivaroxaban treatment in Japanese patients with non‐valvular atrial fibrillation. Heart Vessels. 2020;35:1125‐1134. 32253531 10.1007/s00380-020-01587-z PMC7332477

[25] Netley J , Howard K , Wilson W . Effects of body mass index on the safety and effectiveness of direct oral anticoagulants: a retrospective review. J Thromb Thrombolysis. 2019;48(3):359‐365. 30963393 10.1007/s11239-019-01857-2

[26] Patti G , Pecen L , Manu MC , et al. Thromboembolic and bleeding risk in obese patients with atrial fibrillation according to different anticoagulation strategies. Int J Cardiol. 2020;318:67‐73. 32574823 10.1016/j.ijcard.2020.06.010

[27] Lucijanic M , Jurin I , Jurin H , et al. Patients with higher body mass index treated with direct/novel oral anticoagulants (DOAC/NOAC) for atrial fibrillation experience worse clinical outcomes. Int J Cardiol. 2020;301:90‐95. 31748190 10.1016/j.ijcard.2019.10.035

[28] Wang H , Wang HJ , Chen YD , et al. Prognostic factors of clinical endpoints in elderly patients with atrial fibrillation during a 2‐year follow‐up in China: an observational cohort study. Medicine (United States). 2017;96(33):e7679. 10.1097/MD.0000000000007679 PMC5571683 28816946

[29] Badheka AO , Rathod A , Kizilbash MA , et al. Influence of obesity on outcomes in atrial fibrillation: yet another obesity paradox. Am J Med. 2010;123(7):646‐651. 20609687 10.1016/j.amjmed.2009.11.026

[30] Wang L , Du X , Dong JZ , et al. Body mass index and all‐cause mortality in patients with atrial fibrillation: insights from the China atrial fibrillation registry study. Clin Res Cardiol. 2019;108(12):1371‐1380. 30953181 10.1007/s00392-019-01473-3

[31] Lee CH , Lin TY , Chang SH , et al. Body mass index is an independent predictor of major bleeding in non‐valvular atrial fibrillation patients taking dabigatran. Int J Cardiol. 2017;228:771‐778. 27888754 10.1016/j.ijcard.2016.11.277

[32] Li MH , Hu LH , Xiong YR , et al. Association between body mass index and the risk of bleeding in elderly patients with non‐valvular atrial fibrillation taking dabigatran: a cohort study. J Geriatr Cardiol. 2020;17(4):193‐201. 32362917 10.11909/j.issn.1671-5411.2020.04.008 PMC7189264

[33] Shinohara M , Fujino T , Yao S , et al. Assessment of the bleeding risk of anticoagulant treatment in non‐severe frail octogenarians with atrial fibrillation. J Cardiol. 2019;73(1):7‐13. 29898863 10.1016/j.jjcc.2018.05.012

[34] Boonyawat K , Caron F , Li A , et al. Association of body weight with efficacy and safety outcomes in phase III randomized controlled trials of direct oral anticoagulants: a systematic review and meta‐analysis. J Thromb Haemost. 2017;15(7):1322‐1333. 28407368 10.1111/jth.13701

[35] Steffel J , Verhamme P , Potpara TS , et al. The 2018 European heart Rhythm association practical guide on the use of non‐vitamin K antagonist oral anticoagulants in patients with atrial fibrillation. Eur Heart J. 2018;39(16):1330‐1393. 29562325 10.1093/eurheartj/ehy136

[36] Buys DR , Roth DL , Ritchie CS , et al. Nutritional risk and body mass index predict hospitalization, nursing home admissions, and mortality in community‐dwelling older adults: results from the UAB study of aging with 8.5 years of follow‐up. J Gerontol A Biol Sci Med Sci. 2014;69(9):1146‐1153. 24589863 10.1093/gerona/glu024 PMC4158410

[37] Watson T , Shantsila E , Lip GYH . Mechanisms of thrombogenesis in atrial fibrillation: Virchow's triad revisited. Lancet (London, England). 2009;373(9658):155‐166. 10.1016/S0140-6736(09)60040-4 19135613

[38] Guo Y , Lip GYH , Apostolakis S . Inflammation in atrial fibrillation. J Am Coll Cardiol. 2012;60(22):2263‐2270. 23194937 10.1016/j.jacc.2012.04.063

[39] Visser M , Bouter LM , McQuillan GM , Wener MH , Harris TB . Elevated C‐reactive protein levels in overweight and obese adults. JAMA. 1999;282(22):2131‐2135. 10591334 10.1001/jama.282.22.2131

[40] Ziccardi P , Nappo F , Giugliano G , et al. Reduction of inflammatory cytokine concentrations and improvement of endothelial functions in obese women after weight loss over one year. Circulation. 2002;105(7):804‐809. 11854119 10.1161/hc0702.104279

[41] Nakajima K , Yamaoka H , Morita K , et al. Elderly people with low body weight may have subtle low‐grade inflammation. Obesity. 2009;17(4):803‐808. 19131938 10.1038/oby.2008.596

[42] Weber MA , Neutel JM , Smith DH . Contrasting clinical properties and exercise responses in obese and lean hypertensive patients. J Am Coll Cardiol. 2001;37(1):169‐174. 11153733 10.1016/s0735-1097(00)01103-7

[43] Mohamed‐Ali V , Goodrick S , Bulmer K , Holly JM , Yudkin JS , Coppack SW . Production of soluble tumor necrosis factor receptors by human subcutaneous adipose tissue in vivo. Am J Physiol. 1999;277(6):E971‐E975. 10600783 10.1152/ajpendo.1999.277.6.E971

[44] Hohnloser SH , Fudim M , Alexander JH , et al. Efficacy and safety of Apixaban versus warfarin in patients with atrial fibrillation and extremes in body weight. Circulation. 2019;139(20):2292‐2300. 30773022 10.1161/CIRCULATIONAHA.118.037955

[45] Group JCSJW . Guidelines for pharmacotherapy of atrial fibrillation (JCS 2013). Circulation J. 2014;78(8):1997‐2021. 10.1253/circj.cj-66-0092 24965079

[46] Hori M , Connolly SJ , Zhu J , et al. Dabigatran versus warfarin: effects on ischemic and hemorrhagic strokes and bleeding in Asians and non‐Asians with atrial fibrillation. Stroke. 2013;44(7):1891‐1896. 23743976 10.1161/STROKEAHA.113.000990

[47] Chao TF , Chen SA , Ruff CT , et al. Clinical outcomes, edoxaban concentration, and anti‐factor Xa activity of Asian patients with atrial fibrillation compared with non‐Asians in the ENGAGE AF‐TIMI 48 trial. Eur Heart J. 2019;40(19):1518‐1527. 30590425 10.1093/eurheartj/ehy807

