# Supplementary material for: The impact of underweight and obesity on outcomes in anticoagulated patients with atrial fibrillation: A systematic review and meta‐analysis on the obesity paradox
Source: Clin Cardiol. 2021 Mar 26;44(5):599–608. doi: 10.1002/clc.23593 (PMC8119828; doi:10.1002/clc.23593)
Supplement: Supplementary file 1 — Appendix S1: Supporting information [file CLC-44-599-s001.pdf]

Supplemental materials

Table of contents

Supplemental systematic review 3

Impact of low body weight 3

Supplemental tables 4

eTable 1: Search strategy 4

eTable 2: Overview of included studies for systematic review 5

eTable 3: Assessment of bias within studies 16

eTable 4: PRISMA 2009 checklist 25

Supplemental figures 27

eFigure 1: Impact of overweight on AF-related outcomes 27

eFigure 2: Impact of class II obesity on AF-related outcomes 29

eFigure 3: Impact of morbid obesity on AF-related outcomes 31

eFigure 4: Sensitivity analysis including non-anticoagulated AF patients 32

eFigure 4.1: Impact of underweight on AF-related outcomes (sensitivity analysis) 32

eFigure 4.2: Impact of overweight on AF-related outcomes (sensitivity analysis) 34

eFigure 4.3: Impact of obesity on AF-related outcomes (sensitivity analysis) 36

eFigure 4.4: Impact of class II obesity on AF-related outcomes (sensitivity analysis) 38

eFigure 4.5: Impact of morbid obesity on AF-related outcomes (sensitivity analysis) 40

eFigure 5: Sensitivity analysis for mortality in underweight versus normal BMI AF patients 41

eFigure 6: Sensitivity analysis for major bleeding in underweight versus normal BMI AF patients 44

eFigure 7: Assessment of publication bias 47

References 52

Supplemental systematic review

Impact of low body weight

Although most phase III RCTs did not provide data in underweight AF patients, unadjusted event rates in NOAC- and VKA-treated AF patients with a low body weight have been described. Indeed, higher unadjusted stroke/SE and major bleeding rates were observed in anticoagulated low body weight AF patients in the RE-LY (<50 versus 50-99 kg[1,2], and ≤60 versus >60 kg[3]), the ARISTOTLE (≤60 versus >60-120 kg)[4], ROCKET AF (≤60 versus >60 kg)[5] and ENGAGE AF-TIMI 48 trial (≤60 versus >60 kg[6] and ≤55 versus 79.8-84 kg[7]).[1-7] Moreover, in the ARISTOTLE trial, lower body weight was also associated with a higher all-cause mortality rate in AF patients.[4] After pooling of the results from the 4 phase III RCTs, the meta-analysis of Boonyawat et al. illustrated that AF patients ≤60 kg had a significantly higher stroke/SE risk, but a similar bleeding risk as compared to normal weight AF patients (60-100 kg).[8]

Besides these randomized results, the Fushimi AF registry also provided results in low body weight AF patients, demonstrating significantly higher stroke/SE and all-cause mortality risks but similar major bleeding risks in AF patients ≤50 kg (46% OAC use) as compared to AF patients >50 kg (56% OAC use) after multivariable adjustment.[9] Even in the subset of OAC-treated AF patients ≤50 kg, trends remained consistent. In line, the odds of cardio-embolic stroke among AF patients included in the Fushimi AF registry, was significantly higher per 10 kg decrease in body weight.[10] Moreover, in the SAKURA AF Registry, a low body weight of <50 kg was an independent predictor of all-cause mortality, driven by a significantly higher risk of non-vascular death but not vascular death.[11]

Overall, both randomized and observational studies do observe potentially poorer outcomes in AF patients with a low body weight, mostly in terms of mortality and thromboembolic risks.

Supplemental tables

eTable 1: Search strategy

The following search terms were used in Medline:

**P**atient	“Atrial Fibrillation"[Mesh] OR "Atrial Fibrillation"[TIAB]
**I**ntervention and **C**ontrol	“Apixaban"[TIAB] OR "Apixaban"[Supplementary Concept] OR "Rivaroxaban"[Mesh] OR "Rivaroxaban"[TIAB] OR "Edoxaban"[TIAB] OR “Edoxaban"[Supplementary Concept] OR "Dabigatran"[Mesh] OR "Dabigatran"[TIAB] OR "Antithrombins"[Mesh] OR "Factor Xa Inhibitors"[Mesh] OR "New oral anticoagulants"[TIAB] OR "NOAC"[TIAB] OR "Direct oral anticoagulants"[TIAB] OR "DOAC"[TIAB] OR "Non-vitamin K antagonist oral anticoagulants"[TIAB]
**O**utcome	"Thromboembolism"[Mesh] OR "Thromboembolism"[TIAB] OR "Thrombosis"[TIAB] OR "Stroke"[TIAB] OR "Hemorrhage"[TIAB] OR "Hemorrhage"[Mesh] OR "Bleeding"[TIAB] OR "Mortality"[Mesh] OR "Mortality"[TIAB] OR "Death"[TIAB] OR "Dead"[TIAB]

The following search terms were used in Embase:

**P**atient	('atrial fibrillation':ab,ti OR 'atrial fibrillation'/exp) AND ('body mass'/exp OR ‘BMI’:ab,ti OR ‘body mass’:ab,ti OR 'body weight'/exp OR ‘weight’:ab,ti OR ‘underweight’:ab,ti OR ‘lean’:ab,ti OR ‘overweight’:ab,ti OR 'obesity'/exp OR ‘obesity’:ab,ti OR ‘obese’:ab,ti)
**I**ntervention and **C**ontrol	apixaban:ab,ti OR 'apixaban'/exp OR rivaroxaban:ab,ti OR 'rivaroxaban'/exp OR edoxaban:ab,ti OR 'edoxaban'/exp OR dabigatran:ab,ti OR 'dabigatran'/exp OR 'new oral anticoagulant':ab,ti OR 'new oral anticoagulant'/exp OR ‘NOAC’:ab,ti OR ‘DOAC’:ab,ti OR 'direct oral anticoagulant':ab,ti OR 'direct oral anticoagulant'/exp OR 'non vitamin k antagonist oral anticoagulant':ab,ti OR 'non-vitamin k antagonist oral anticoagulant':ab,ti OR 'non vitamin k antagonist oral anticoagulant'/exp OR 'non vitamin k oral anticoagulant':ab,ti OR 'non-vitamin k oral anticoagulant':ab,ti
**O**utcome	‘thromboembolism’:ab,ti OR 'thromboembolism'/exp OR ‘thrombosis’:ab,ti OR 'thrombosis'/exp OR ‘stroke’:ab,ti OR 'stroke'/exp OR ‘hemorrhage’:ab,ti OR ‘haemorrhage’:ab,ti OR ‘bleeding’:ab,ti OR 'bleeding'/exp OR 'mortality'/exp OR 'death':ab,ti OR 'dead':ab,ti OR 'mortality':ab,ti

eTable 1: Search strategy.

eTable 2: Overview of included studies for systematic review

Author	Study design	Study cohort	n	Mean/median age (years +/- SD; [IQR])	Mean/median follow-up (+/- SD; [IQR])	Stroke/SE (HR [95% CI])	Major bleeding (HR [95% CI])	Intracranial bleeding (HR [95% CI])	Gastrointestinal bleeding (HR [95% CI])	All-cause mortality (HR [95% CI])
Sandhu et al. 2016[12]*	Phase III RCT (worldwide)	AF patients included in the ARISTOTLE trial (api vs warf), categorized according to BMI 18.5-<25, ≥25-<30, ≥30 kg/m². Exploratory analysis according to class I (BMI 30-<35), II (35-<40) and III obesity (≥40 kg/m²). Industry-sponsored.	**BMI 18.5-<25:** 4052; **25-<30:** 6702; **≥30:** 7159; **30-<35:** 4364; **35-<40:** 1764; **≥40:** 1003	**BMI 18.5-<25:** 71.3y +/- 10.0; **25-<30:** 70.1y +/- 9.3; **≥30:** 66.8y +/- 9.2; (NR for other BMI subgroups)	1.8 years (overall, NR for BMI subgroups)	BMI 25-<30 vs <25:<br>0.86 [0.68-1.08]<br>BMI ≥30 vs <25:<br>0.79 [0.61-1.02]<br>BMI ≥30-<35 vs <25:<br>0.86 [0.65-1.13]<br>BMI ≥35-<40 vs <25:<br>**0.60 [0.39-0.92]**<br>BMI ≥40 vs <25:<br>0.74 [0.44-1.24]	BMI 25-<30 vs <25:<br>**0.82 [0.68-0.99]**<br>BMI ≥30 vs <25:<br>0.91 [0.74-1.10]<br>BMI ≥30-<35 vs <25:<br>0.94 [0.76-1.16]<br>BMI ≥35-<40 vs <25:<br>0.88 [0.66-1.18]<br>BMI ≥40 vs <25:<br>0.70 [0.46-1.06]	NR	NR	BMI 25-<30 vs <25:<br>**0.67 [0.59-0.78]**<br>BMI ≥30 vs <25:<br>**0.63 [0.54-0.74]**<br>BMI ≥30-<35 vs <25:<br>**0.64 [0.54-0.76]**<br>BMI ≥35-<40 vs <25:<br>**0.57 [0.45-0.73]**<br>BMI ≥40 vs <25:<br>**0.70 [0.52-0.94]**
Hohnloser et al. 2019[4]	Phase III RCT (worldwide)	AF patients included in the ARISTOTLE trial (api vs warf), categorized according to body weight ≤60, >60-120 and >120 kg. Industry-sponsored.	**≤60 kg:** 1985; **>60-120 kg:** 15 172; **>120 kg:** 982	**≤60 kg:** 74y [66-79]; **>60-120 kg:** 70y [63-76]; **>120 kg:** 62y [56-67]	21.8 months [16.3-28.1] (overall, NR for BMI subgroups)	***Incidence rate:***<br>≤60 vs >60-120 kg:<br>**Api:** 2.01%/y vs 1.23%/y<br>**Warf:** 3.20%/y vs 1.44%/y	***Incidence rate:***<br>≤60 vs >60-120 kg:<br>**Api:** 2.33%/y vs 2.15%/y<br>**Warf:** 4.28%/y vs 3.02%/y	NR	NR	***Incidence rate:***<br>≤60 vs >60-120 kg:<br>**Api:** 7.00%/y vs 3.14%/y<br>**Warf:** 6.33%/y vs 3.75%/y
Balla et al. 2017[13]*	Phase III RCT (worldwide)	AF patients included in the ROCKET AF trial (riva vs warf), categorized according to BMI 18.5-<25, ≥25-<30, ≥30 kg/m². Exploratory analysis according to class I (BMI 30-<35) and II-III (≥35 kg/m²). Industry-sponsored.	**BMI 18.5-<25:** 3289; **25-<30:** 5535; **≥30:** 5206; **30-<35:** 3308; **≥35:** 1898	**BMI 18.5-<25:** 75.0y [68.0-80.0]; **25-<30:** 74.0y [67.0-78.0]; **≥30:** 70.0y [62.0-76.0]; (NR for other subgroups)	2 years (overall, NR for BMI subgroups)	BMI 25-<30 vs <25:<br>**0.81 [0.66-0.99]**<br>BMI ≥30 vs <25:<br>**0.69 [0.55-0.86]**<br>BMI ≥30-<35 vs <25:<br>**0.71 [0.56-0.89]**<br>BMI ≥35 vs <25:<br>**0.54 [0.40-0.73]**	BMI 25-<30 vs <25:<br>1.09 [0.91-1.31]<br>BMI ≥30 vs <25:<br>1.06 [0.87-1.29]<br>BMI ≥30-<35 vs <25:<br>0.89 [0.72-1.10]<br>BMI ≥35 vs <25:<br>0.94 [0.74-1.20]	BMI 25-<30 vs <25:<br>1.05 [0.61-1.80]<br>BMI ≥30 vs <25:<br>0.75 [0.41-1.38]<br>BMI ≥30-<35 vs <25:<br>0.56 [0.29-1.10]<br>BMI ≥35 vs <25:<br>0.56 [0.25-1.25]	NR	NR
Bayer AG, 2011[5]	Phase III RCT (worldwide)	Rivaroxaban FDA drug label (SmPC), results from ROCKET AF trial, categorized according to weight ≤60 kg and >60 kg. Industry-sponsored.	**≤60 kg:** 777 riva, 775 warf; **>60 kg:** 6302 riva, 6314 warf	73y [65-78] riva, 73y [65-78] warf (overall, NR for weight subgroups)	2 years (overall, NR for weight subgroups)	***Incidence rate:***<br>≤60 vs >60 kg:<br>**Riva:** 2.7%/y vs 2.1%/y<br>**Warf:** 3.8%/y vs 2.3%/y	***Incidence rate:***<br>≤60 vs >60 kg:<br>**Riva:** 2.9%/y vs 3.7%/y<br>**Warf:** 4.1%/y vs 3.4%/y			

Boriani et al. 2019[14]*	Phase III RCT (worldwide)	AF patients included in the ENGAGE AF-TIMI 48 trial (edo vs warf), categorized according to BMI <18.5, 18.5-<25, ≥25-<30, 30-<35, 35-<40 and ≥40 kg/m². Industry-sponsored.	**BMI <18.5:** 177;<br>**18.5-<25:** 4491;<br>**25-<30:** 7903;<br>**30-<35:** 5209;<br>**35-<40:** 2099;<br>**≥40:** 1149	**BMI <18.5:** NR;<br>**18.5-<25:** 75y [70-81];<br>**25-<30:** 73y [67-79];<br>**30-<35:** 71y [65-78];<br>**35-<40:** 68y [61-75];<br>**≥40:** 64y [58-70]	2.8 years (overall, NR for BMI subgroups)	BMI <18.5 vs 18.5-<25:<br>*1.96 [1.18-3.28]*<br>BMI 25-<30 vs 18.5-<25:<br>0.91 [0.78-1.07]<br>BMI ≥30-<35 vs 18.5-<25:<br>0.82 [0.68-1.00]<br>BMI ≥35-<40 vs 18.5-<25:<br>**0.68 [0.52-0.89]**<br>BMI ≥40 vs 18.5-<25:<br>**0.54 [0.35-0.83]**	BMI <18.5 vs 18.5-<25:<br>1.55 [0.88-2.74]<br>BMI 25-<30 vs 18.5-<25:<br>1.03 [0.88-1.20]<br>BMI ≥30-<35 vs 18.5-<25:<br>1.12 [0.94-1.34]<br>BMI ≥35-<40 vs 18.5-<25:<br>1.18 [0.94-1.48]<br>BMI ≥40 vs 18.5-<25:<br>1.28 [0.96-1.70]	NR	NR	BMI <18.5 vs 18.5-<25:<br>*2.68 [1.98-3.63]*<br>BMI 25-<30 vs 18.5-<25:<br>**0.79 [0.71-0.87]**<br>BMI ≥30-<35 vs 18.5-<25:<br>**0.77 [0.68-0.88]**<br>BMI ≥35-<40 vs 18.5-<25:<br>**0.75 [0.63-0.90]**<br>BMI ≥40 vs 18.5-<25:<br>**0.78 [0.62-0.98]**
Boriani et al. 2020[7]	Phase III RCT (worldwide)	AF patients included in the ENGAGE AF-TIMI 48 trial (edo 60/30 vs warf), categorized according to ≤55 kg, 79.8-84 kg, and ≥120 kg. Industry-sponsored.	**≤55 kg:** 344 edo 60/30, 368 warf;<br>**79.8-84 kg:** 707 edo, 717 warf;<br>**≥120 kg:** 381 edo, 370 warf	**≤55 kg:** 76 [69-80] edo, 76 [69-80] warf;<br>**79.8-84 kg:** 73 [67-78] edo, 73 [66-78] warf;<br>**≥120 kg:** 62 [57-68] edo, 62 [56-68] edo,	2.8 years (overall, NR for weight subgroups)	***Incidence:***<br>≤55 vs 79.8-84 kg:<br>**Edo:** 7.3% vs 3.5%<br>**Warf:** 6.5% vs 4.7%<br>≥120 vs 79.8-84 kg:<br>**Edo:** 2.1% vs 3.5%<br>**Warf:** 1.6% vs 4.7%	***Incidence:***<br>≤55 vs 79.8-84 kg:<br>**Edo:** 5.3% vs 5.8%<br>**Warf:** 9.3% vs 7.7%<br>≥120 vs 79.8-84 kg:<br>**Edo:** 6.3% vs 5.8%<br>**Warf:** 6.5% vs 7.7%	NR	NR	***Number of events:***<br>≤55 vs 79.8-84 kg:<br>**Edo:** 55 vs 71<br>**Warf:** 78 vs 75<br>≥120 vs 79.8-84 kg:<br>**Edo:** 36 vs 71<br>**Warf:** 31 vs 75
Daiichi Sankyo, 2015[6]	Phase III RCT (worldwide)	Savaysa (edoxaban) FDA drug label (SmPC), results from ENGAGE AF-TIMI 48 trial, high-dose edo regimen (edo 60/30; use of reduced dose edo 30 mg if weight ≤60 kg) vs warf, categorized according to weight ≤60 kg and >60 kg. Industry-sponsored.	**≤60 kg:** 684 edo, 701 warf;<br>**>60 kg:** 6351 edo, 6335 warf	72y [64-78] edo 60/30, 72y [64-78] warf (overall, NR for ≤60 kg subgroup)	2.8 years (overall, NR for ≤60 kg subgroup)	***Incidence rate:***<br>≤60 vs >60 kg:<br>**Edo:** 2.53%/y vs 1.47%/y<br>**Warf:** 2.91%/y vs 1.69%/y	***Incidence rate:***<br>≤60 vs >60 kg:<br>**Edo:** 2.95%/y vs 2.65%/y<br>**Warf:** 4.44%/y vs 3.26%/y	NR	NR	NR
Connolly et al. 2009[1]	Phase III RCT (worldwide)	AF patients included in the RE-LY trial (dabi 150/110 vs warf), categorized according to weight <50 kg, 50-99 kg, ≥100 kg. Industry-sponsored.	**<50 kg:** 376;<br>**50-99 kg:** 14 629;<br>**≥100 kg:** 3099	71.5y +/- 8.8 dabi 150, 71.4y +/- 8.6 dabi 110, 71.6y +/- 8.6 warf	2.0 years (11 899 PY dabi 110, 12 033 PY dabi 150, 11 794 PY warf)	***Incidence rate:***<br><50 kg vs 50-99 kg:<br>**Dabi 150:** 2.24%/y vs 1.14%/y<br>**Dabi 110:** 2.58%/y vs 1.16%/y	NR	NR	NR	NR

				(overall, NR for weight subgroups)	(overall, NR for weight subgroups)	**Warf:** 5.04%/y vs 1.77%/y				
Eikelboom et al. 2011[2]	Phase III RCT (worldwide)	AF patients included in the RE-LY trial (dabi 150/110 vs warf), categorized according to weight <50 kg, 50-99 kg, ≥100 kg. Industry-sponsored.	**<50 kg:** 376; **50-99 kg:** 14 629; **≥100 kg:** 3099	71.5y +/- 8.8 dabi 150, 71.4y +/- 8.6 dabi 110, 71.6y +/- 8.6 warf (overall, NR for weight subgroups)	2.0 years (11 899 PY dabi 110, 12 033 PY dabi 150, 11 794 PY warf) (overall, NR for weight subgroups)	NR	***Incidence rate:*** <u><50 kg vs 50-99 kg:</u> **Dabi 150:** 4.92%/y vs 3.13%/y **Dabi 110:** 4.30%/y vs 2.91%/y **Warf:** 5.95%/y vs 3.57%/y	NR	NR	NR
Boehringer Ingelheim, 2010[15]*	Phase III RCT (worldwide)	Dabigatran FDA Advisory Committee Briefing Document, subgroup analysis in the RE-LY trial, categorized according to BMI 18.5-<25, ≥25-<30, ≥30-<35 and ≥35 kg/m². Industry-sponsored.	**BMI 18.5-<25:** 4697; **≥25-<30;** 7111; **≥30:** 6279; **≥30-<35:** 4038; **≥35:** 2241	71.5y +/- 8.8 dabi 150, 71.4y +/- 8.6 dabi 110, 71.6y +/- 8.6 warf (overall, NR for BMI subgroups)	2.0 years (11 899 PY dabi 110, 12 033 PY dabi 150, 11 794 PY warf) (overall, NR for BMI subgroups)	***Number of events:*** <u>BMI 18.5-<25:</u> 181 <u>BMI 25-<30:</u> 196 <u>BMI ≥30:</u> 144 <u>BMI 30-<35:</u> 91 <u>BMI ≥35:</u> 53 ***Incidence rate:*** <u>BMI 25-<30 vs <25:</u> **Dabi 150:** 1.16%/y vs 1.33%/y **Dabi 110:** 1.54%/y vs 1.89%/y **Warf:** 1.49%/y vs 2.65%/y <u>BMI ≥30-<35 vs <25:</u> **Dabi 150:** 0.99%/y vs 1.33%/y **Dabi 110:** 1.28%/y vs 1.89%/y **Warf:** 1.16%/y vs 2.65%/y <u>BMI ≥35 vs <25:</u> **Dabi 150:** 0.76%/y vs 1.33%/y **Dabi 110:** 1.32%/y vs 1.89%/y **Warf:** 1.49%/y vs 2.65%/y	***Number of events:*** <u>BMI 18.5-<25:</u> 344 <u>BMI 25-<30:</u> 424 <u>BMI ≥30:</u> 394 <u>BMI 30-<35:</u> 247 <u>BMI ≥35:</u> 147 ***Incidence rate:*** NR	NR	NR	NR
Boehringer Ingelheim, 2010[3]	Phase III RCT (worldwide)	Dabigatran FDA drug label (SmPC), results from RE-LY trial, categorized according	**≤60 kg:** 1331 (647 dabi 150, 684 warf);	71.5y +/- 8.8 dabi 150, 71.6y +/- 8.6 warf	2.0 years (12 033 PY dabi 150,	***Incidence rate:*** <u>≤60 kg vs >60 kg:</u> **Dabi 150:** 1.68%/y vs 1.06%/y	***Incidence rate:*** <u>≤60 kg vs >60 kg:</u> **Dabi 150:** 4.59%/y vs 3.35%/y			

		to weight ≤60 kg and >60 kg. Industry-sponsored.	**>60 kg:**<br>10 762 (5428 dabi 150, 5334 warf)	(overall, NR for BMI subgroups)	11 794 PY warf) (overall, NR for BMI subgroups)	**Warf:** 3.32%/y vs 1.53%/y	**Warf:** 4.78%/y vs 3.45%/y			
Proietti et al. 2017[16]	Meta-analysis	Pooled data of 3 phase III RCTs in AF (RE-LY, ROCKET AF, ARISTOTLE), categorized according to BMI 18.5-<25, ≥25-<30, ≥30 kg/m².	**BMI 18.5-<25:**<br>12 063;<br>**25-<30:**<br>19 336;<br>**≥30:**<br>18 632	NR	NR	BMI 25-<30 vs <25:<br>**OR 0.75 [0.66-0.84]**<br>BMI ≥30 vs <25:<br>**OR 0.62 [0.54-0.70]**<br>BMI ≥30 vs 25-<30:<br>**OR 0.83 [0.73-0.94]**	BMI 25-<30 vs <25:<br>OR 0.84 [0.70-1.01]<br>BMI ≥30 vs <25:<br>**OR 0.84 [0.72-0.98]**<br>BMI ≥30 vs 25-<30:<br>OR 1.00 [0.92-1.10]	NR	NR	NR
Boonyawat et al. 2017[8]	Meta-analysis	Pooled data of 11 phase III RCTs (5 in AF, 6 in VTE, separate data for AF reported based on RE-LY, ROCKET AF, ARISTOTLE and/or ENGAGE AF-TIMI 48 trial), categorized according to body weight ≤60 kg, 60-100 kg and ≥100 kg; obesity (BMI ≥30 kg/m²) vs non-obesity (BMI <30 kg/m²)	**≤60 kg:**<br>6003<br>**60-100 kg:**<br>66 657<br>**≥100 kg:**<br>4116;<br><br>**BMI <30:**<br>31 704<br>**≥30**:<br>18 626	NR	NR	≤60 kg vs 60-100:<br>*RR 1.72 [1.49-1.98]*	***Major or CRNMB:***<br>≤60 kg vs 60-100:<br>RR 1.00 [0.83-1.19]	NR	NR	NR
Zhou et al. 2020[17]	Meta-analysis	Pooled data of 5 phase III RCTs in AF (RE-LY, ROCKET AF, ARISTOTLE, ENGAGE AF-TIMI 48, SPORTIF) and one observational cohort study (in underweight patients), categorized according to underweight (BMI <18.5 of body weight <60 kg), normal BMI (BMI 18.5-<25), overweight (BMI ≥25-<30), obesity (BMI ≥30), class I obesity (BMI ≥30-<35), class II obesity (≥35-<40) and	NR	NR	NR	BMI <18.5 vs 18.5-25:<br>*RR 1.98 [1.19-3.28]*<br>BMI ≥25-<30 vs 18.5-25:<br>**RR 0.81 [0.71-0.91]**<br>BMI ≥30 vs 18.5-25:<br>**RR 0.69 [0.61-0.78]**<br>BMI ≥30-<35 vs 18.5-25:<br>**RR 0.79 [0.69-0.89]**<br>BMI ≥35-<40 vs 18.5-25:<br>**RR 0.66 [0.52-0.82]**<br>BMI ≥40 vs 18.5-25:<br>**RR 0.61 [0.44-0.86]**	BMI <18.5 vs 18.5-25:<br>RR 2.10 [0.89-4.92]<br>BMI ≥25-<30 vs 18.5-25:<br>RR 0.93 [0.79-1.08]<br>BMI ≥30 vs 18.5-25:<br>RR 1.04 [0.91-1.18]<br>BMI ≥30-<35 vs 18.5-25:<br>RR 0.99 [0.86-1.14]<br>BMI ≥35-<40 vs 18.5-25:<br>RR 1.03 [0.78-1.38]<br>BMI ≥40 vs 18.5-25:<br>RR 0.97 [0.53-1.74]	NR	NR	BMI <18.5 vs 18.5-25:<br>RR 4.34 [0.57-32.83]<br>BMI ≥25-<30 vs 18.5-25:<br>**RR 0.73 [0.64-0.83]**<br>BMI ≥30 vs 18.5-25:<br>**RR 0.72 [0.66-0.79]**<br>BMI ≥30-<35 vs 18.5-25:<br>**RR 0.71 [0.59-0.85]**<br>BMI ≥35-<40 vs 18.5-25:<br>**RR 0.66 [0.51-0.86]**<br>BMI ≥40 vs 18.5-25:<br>**RR 0.75 [0.62-0.90]**

		class III obesity (≥40kg/m²).								
Badheka et al. 2010[18]	Observational prospective multicentre cohort study (USA)	AF patients included in the AFFIRM study, 87% VKA-treated, categorized according to BMI 18.5-<25, ≥25-<30 and ≥30 kg/m².	**BMI 18.5-<25:** 637;<br>**25-<30:** 965;<br>**≥30:** 890	**BMI 18.5-<25:** 72.4y +/- 7;<br>**25-<30:** 70.7y +/- 7.5;<br>**≥30:** 66.4 +/- 8.4	3 years +/- 0.9 (overall, NR for BMI subgroups)	NR	NR	NR	NR	BMI 25-<30 vs <25:<br>**0.64 [0.48-0.84]**<br>BMI ≥30 vs <25:<br>**0.80 [0.68-0.93]**<br><br>***Number of events:***<br>BMI 18.5-<25: 103<br>BMI 25-<30: 108 evets<br>BMI ≥30: 93
Overvad et al. 2013[19]	Observational prospective multicentre cohort study (Denmark)	Patients included in the Diet, Cancer and Health study who developed incident AF during follow-up, categorized according to BMI 18.5-<25 (19% VKA-treated), ≥25-<30 (23% VKA-treated) and ≥30 kg/m² (24% VKA-treated).	**BMI 18.5-<25:** 954;<br>**25-<30:** 1414;<br>**≥30:** 767	**BMI 18.5-<25:** 67.2y [59.5-74.4] (P10-P90);<br>**25-<30:** 67.0y [59.5-74.3];<br>**≥30:** 66.9y [58.7-73.5]	**BMI 18.5-<25:** 4.7y [0.8-10.8] (P10-P90);<br>**25-<30:** 4.9y [1.0-11.4];<br>**≥30:** 5.0y [1.1-11.1]	BMI 25-<30 vs <25:<br>1.14 [0.83-1.57]<br>BMI ≥30 vs <25:<br>0.98 [0.67-1.42]	NR	NR	NR	BMI 25-<30 vs <25:<br>*1.31 [1.07-1.59]*<br>BMI ≥30 vs <25:<br>*1.41 [1.13-1.75]*
Hamatani et al. 2015[9]	Observational prospective multicentre cohort study (Japan)	AF patients included in the Fushimi AF Registry, stratified according to weight ≤50 kg and >50 kg (OAC use in 46% and 56% respectively, almost only VKAs)	**≤50 kg:** 788 (364 OAC use);<br>**>50 kg:** 2157 (1206 OAC use)	**≤50 kg:** 79.6y +/- 9.8;<br>**>50 kg:** 71.8y +/- 10.2	746 days [404-1109] (overall, NR for weight subgroups)	***Overall:***<br>≤50 vs >50 kg:<br>*2.19 [1.57-3.04]*<br><br>***OAC-treated:***<br>≤50 vs >50 kg:<br>*1.94 [1.23-2.98]*	***Overall:***<br>≤50 vs >50 kg:<br>1.05 [0.64-1.68]<br><br>***OAC-treated:***<br>≤50 vs >50 kg:<br>0.68 [0.30-1.37]	NR	NR	***Overall:***<br>≤50 vs >50 kg:<br>*2.74 [2.25-3.32]*<br><br>***OAC-treated:***<br>≤50 vs >50 kg:<br>NR
Hamatani et al. 2015[20]	Observational prospective multicentre cohort study (Japan)	Non-anticoagulated AF patients included in the Fushimi AF Registry, identifying predictors for composite stroke/SE and mortality	**Overall:** 1245;<br>**BMI <18.5:** 180	73.1y +/- 12.5 (overall)	748 days [458-1112] (overall, NR for BMI subgroups)	***Stroke/SE/mortality:***<br>BMI <18.5 vs ≥18.5:<br>*1.71 [1.25-2.32]*	NR	NR	NR	NR
Pandey et al. 2016[21]	Observational prospective multicentre cohort study (USA)	AF patients included in the ORBIT-AF Registry, categorized according to BMI 18.5-<25 (69% VKA-treated), ≥25-<30 (71% VKA-treated), 30-<35 (72% VKA-treated), 35-<40 (74% VKA-	**BMI 18.5-<25:** 2076;<br>**25-<30:** 3164;<br>**30-<35:** 2173;<br>**35-<40:**	**BMI 18.5-<25:** 80y [73-85];<br>**25-<30:** 77y [69-83];<br>**30-<35:** 73y [66-79];<br>**35-<40:** 70y [62-76];	26.8 months +/- 9.6 (overall, NR for BMI subgroups)	BMI 25-<30 vs <25:<br>0.92 [0.68-1.24]<br>BMI ≥30-<35 vs <25:<br>0.94 [0.63-1.40]<br>BMI ≥35-<40 vs <25:<br>0.73 [0.40-1.31]<br>BMI ≥40 vs <25:<br>1.00 [0.48-2.07]	***Bleeding-related hospitalization:***<br>BMI 25-<30 vs <25:<br>1.15 [0.93-1.42]<br>BMI ≥30-<35 vs <25:<br>1.05 [0.82-1.33]	NR	NR	BMI 25-<30 vs <25:<br>**0.81 [0.70-0.95]**<br>BMI ≥30-<35 vs <25:<br>**0.65 [0.54-0.78]**<br>BMI ≥35-<40 vs <25:<br>0.85 [0.68-1.06]

		treated) and ≥40 kg/m² (75% VKA-treated).	1158;<br>**≥40:** 942	**≥40:**<br>67y [60-73]			BMI ≥35-<40 vs <25:<br>1.17 [0.87-1.58]<br>BMI ≥40 vs <25:<br>1.25 [0.90-1.74]			BMI ≥40 vs <25:<br>**0.73 [0.56-0.97]**
Inoue et al. 2016[22]	Observational prospective multicentre cohort study (Japan)	AF patients included in the J-RHYTHM Registry, categorized according to BMI <18.5 (86% VKA-treated), 18.5-<25 (87% VKA-treated), ≥25-<30 (88% VKA-treated), and ≥30 kg/m² (91% VKA-treated). Industry-sponsored.	**BMI <18.5:**<br>386;<br>**18.5-<25:**<br>3979;<br>**25-<30:**<br>1739;<br>**≥30:**<br>275	**BMI <18.5:**<br>75y +/- 10;<br>**18.5-<25:**<br>70y +/- 10;<br>**25-<30:**<br>68 +/- 10;<br>**≥30:**<br>65y +/- 11	2 year (overall, NR for BMI subgroups)	BMI <18.5 vs 18.5-<25:<br>1.22 [0.63-2.38]<br>BMI 25-<30 vs 18.5-<25:<br>0.94 [0.60-1.46]<br>BMI ≥30 vs 18.5-<25:<br>0.71 [0.22-2.27]	BMI <18.5 vs 18.5-<25:<br>1.71 [0.96-3.05]<br>BMI 25-<30 vs 18.5-<25:<br>0.74 [0.47-1.16]<br>BMI ≥30 vs 18.5-<25:<br>0.87 [0.35-2.19]	NR	NR	BMI <18.5 vs 18.5-<25:<br>*2.40 [1.59-3.63]*<br>BMI 25-<30 vs 18.5-<25:<br>**0.60 [0.37-0.95]**<br>BMI ≥30 vs 18.5-<25:<br>1.70 [0.84-3.44]
Senoo et al. 2016[23]	Phase III RCT (worldwide)	AF patients ≥75 years old included in the AMADEUS trial (unapproved idraparinux vs warf), stopped earlier due to excessive (intracranial) bleeding with idraparinux. However, separate results in warfarin only arm provided, categorized according to BMI 18.5-<25, ≥25-<30, 30-<35, 35-<40 and ≥40 kg/m². Industry-sponsored.	***Warfarin arm:***<br>814 (NR for BMI subgroups)<br><br>***Overall:***<br>**BMI 18.5-<25:**<br>515;<br>**25-<30:**<br>711;<br>**≥30:**<br>362	***Overall:***<br>**BMI 18.5-<25:**<br>79.7y +/- 3.7;<br>**25-<30:**<br>79.1y +/- 3.3;<br>**≥30:**<br>78.6y +/- 3.0<br><br>*(NR for warfarin arm)*	NR	***Warfarin arm:***<br>***Stroke/SE/cardio-vascular mortality:***<br>BMI ≥30 vs 18.5-<25:<br>0.53 [0.18-1.15]	NR	NR	NR	NR
Lee et al. 2017[24]	Observational retrospective single centre cohort study (Taiwan)	AF patients using dabigatran 110 mg identified using the electronic health care database of a medical centre, categorized according to the BMI tertiles (≤23.9, 23.9-26.5, >26.5 kg/m²).	**BMI ≤23.9:**<br>273;<br>**23.9-26.5:**<br>290;<br>**>26.5:**<br>279	**BMI ≤23.9:**<br>86.1% ≥65y;<br>**23.9-26.5:**<br>88.6% ≥65y;<br>**>26.5:**<br>84.2% ≥65y<br>(mean/median age NR)	20 months (overall, NR for BMI subgroups)	NR	Per 1 kg/m² increase in BMI:<br>**0.84 [0.75-0.95]**	NR	NR	NR

Wang et al. 2017[25]	Observational prospective single centre cohort study (China)	AF patients >65 years old included at the General Hospital of the People's Liberation Army, China, between 2014-2015 (19% OAC treated, 96% male). Age- and sex-matched patients >65 years old without AF selected from same hospital as control group (separate results in AF subgroup provided).	**AF patients >65y:** 194 (overall, NR for BMI subgroups)	**AF patients >65y:** 86.6y +/- 8.4 (overall, NR for BMI subgroups)	2 years (overall, NR for BMI subgroups)	***Stroke/SE/MI:*** Per 1 kg/m² increase in BMI: *1.09 [1.03-1.14]*	NR	NR	NR	NR
Park et al. 2017[26*]	Observational retrospective single centre cohort study (Korea)	AF patients treated with NOACs (dabi, riva, api; 64% reduced dose), recruited between 2012 and 2016 at Seoul National University Hospital, retrospective review of electronic medical records, categorized according to BMI <18.5, 18.5-<25 and ≥25 kg/m², multivariable adjusted.	**BMI <18.5:** 62; **18.5-<25:** 753; **≥25:** 538	**BMI <18.5:** 77.4y +/- 7.1; **18.5-<25:** 72.9y +/- 8.9; **≥25:** 71.7y +/- 8.9	7 months	BMI <18.5 vs 18.5-<25: 1.13 [0.13-9.80] BMI ≥25 vs 18.5-<25: 0.17 [0.021-1.34]	BMI <18.5 vs 18.5-<25: *4.14 [1.44-11.85]* BMI ≥25 vs 18.5-<25: 0.77 [0.31-1.96]	NR	NR	BMI <18.5 vs 18.5-<25: *10.52 [2.95-37.56]* BMI ≥25 vs 18.5-<25: 0.92 [0.22-3.93]
Yasuda et al. 2018[10]	Observational prospective multicentre cohort study (Japan)	AF patients included in the Fushimi AF Registry, stratified according to patients with and without cardio-embolic stroke (OAC use in 59% and 53% respectively, almost only VKAs), predictors for stroke after multivariable adjustment.	**AF patients with stroke:** 91; **AF patients without stroke:** 3658 (overall, NR for weight subgroups)	**AF patients with stroke:** 78.9y +/- 8.4; **AF patients without stroke:** 73.5y +/- 11.0	979 days +/- 7.7	***Cardio-embolic stroke:*** Per 10 kg decrease in body weight: *OR 1.30 [1.03-1.65]*	NR	NR	NR	NR

Shinohara et al. 2019[27]	Observational retrospective single centre cohort study (Japan)	AF patients ≥80 years (266 NOAC, 80 warf users; 75% appropriately on-label dosed), predictors for bleeding after multivariable adjustment.	**Overall:** 346 (256 on-label dosed); **BMI <18.5:** 79	83.7y [81.0-85.0] (overall, NR for BMI subgroups)	32.7 months [14.0-51.0]	NR	***Any (major or minor) bleeding:***<br>BMI <18.5 vs ≥18.5:<br>*3.26 [1.65-6.50]*<br><br>***On-label dosing:***<br>BMI <18.5 vs ≥18.5:<br>*2.17 [1.01-4.70]*	NR	NR	NR
Kuronuma et al. 2019[11]	Observational prospective multicentre cohort study (Japan)	AF patients included in the SAKURA AF Registry, OAC-naïve and –experienced (48% warf, 52% NOAC), predictors of (non-) vascular and all-cause death after multivariable adjustment. Industry-sponsored.	**Overall:** 3237 (20% OAC-naïve) (NR for weight subgroups)	72.0y +/- 9.4 (overall, NR for weight subgroups)	39.3 months (range: 28.5-43.6 months)	NR	NR	NR	NR	***All-cause mortality:***<br><50 kg vs ≥50 kg:<br>*1.57 [1.06-2.33]*<br><br>***Vascular mortality:***<br><50 kg vs ≥50 kg:<br>1.63 [0.87-3.08]<br><br>***Non-vascular mortality:***<br><50 kg vs ≥50 kg:<br>*2.09 [1.21-3.59]*
Wang et al. 2019[28]	Observational prospective multicentre cohort study (China)	AF patients included in the China-AF study, categorized according to BMI <18.5 (36% warf, 6% NOAC), 18.5-<24.0 (36% warf, 6% NOAC), ≥24-<28 (38% warf, 7% NOAC) and ≥28 kg/m² (42% warf, 7% NOAC).	**BMI <18.5:** 251; **18.5-<24:** 3650; **≥24-<28:** 4856; **≥28:** 2185	**BMI <18.5:** 73.7y +/- 10.9; **18.5-<24:** 68.6y +/- 11.9; **≥24-<28:** 66.1y +/- 11.3; **≥28:** 64.1y +/- 11.9	30 months [18-48]	NR	NR	NR	NR	BMI <18.5 vs 18.5-<24:<br>*2.23 [1.67-2.97]*<br>BMI ≥24-<28 vs 18.5-<24:<br>**0.70 [0.61-0.81]**<br>BMI ≥28 vs 18.5-<24:<br>**0.54 [0.44-0.67]**
Netley et al. 2019[29]	Observational retrospective multicentre cohort study (USA)	AF patients treated NOACs, admitted to one of nine hospitals within a Midwestern community health system, categorized according to BMI <30, 30-40 and >40 kg/m². Charts were retrospectively reviewed for effectiveness and safety outcomes.	**BMI <30:** 1575; **30-40:** 1288; **>40:** 595	**BMI <30:** 65.9y; **30-40:** 57.0y; **>40:** 49.8y	NR	***Stroke/SE/VTE: Events:***<br>BMI <30: 1.3% (21/1575)<br>BMI 30-40: 1.0% (13/1288)<br>BMI >40: 1.5% (9/595)<br>(p-value for trend 0.598)	***Major or clinically-relevant non-major bleeding: Events:***<br>BMI <30: 2.6% (41/1575)<br>BMI 30-40: 1.7% (22/1288)<br>BMI >40: 1.2% (7/595)<br>(p-value for trend 0.065)	NR	NR	NR

Murakawa et al. 2020[30]*	Observational prospective multicentre cohort study (Japan)	AF patients treated with rivaroxaban (15/10 mg) included in the XAPASS trial, categorized according to BMI <18.5 kg/m², 18.5-<25, ≥25-<30 and ≥30 kg/m².	**BMI <18.5:** 542;<br>**18.5-<25:** 4410;<br>**≥25-<30:** 2167;<br>**≥30:** 499	**BMI <18.5:** 78.1y +/- 9.0<br>**18.5-<25:** 73.6y +/- 9.6<br>**≥25-<30:** 71.3y +/- 9.6<br>**≥30:** 69.5y +/- 11.0	1 year (overall, NR for BMI subgroups)	***Stroke/SE/MI:***<br>BMI <18.5 vs 18.5-<25:<br>1.64 [0.90-2.99]<br>BMI 25-<30 vs 18.5-<25:<br>1.19 [0.76-1.84]<br>BMI ≥30 vs 18.5-<25:<br>1.15 [0.52-2.57]	BMI <18.5 vs 18.5-<25:<br>0.97 [0.48-1.98]<br>BMI 25-<30 vs 18.5-<25:<br>0.97 [0.63-1.50]<br>BMI ≥30 vs 18.5-<25:<br>0.89 [0.40-1.97]	NR	NR	BMI <18.5 vs 18.5-<25:<br>*3.56 [2.40-5.26]*<br>BMI 25-<30 vs 18.5-<25:<br>1.19 [0.77-1.83]<br>BMI ≥30 vs 18.5-<25:<br>0.74 [0.27-2.05]
Bertomeu-Gonzalez et al. 2020[31]*	Observational prospective multicentre cohort study (Spain)	VKA- and NOAC-treated AF patients included in the FANTASIIA Registry, categorized according to BMI 18.5-<25, ≥25-<30 and ≥30 kg/m². Industry-sponsored.	**BMI 18.5-<25:** 358;<br>**≥25-<30:** 871;<br>**≥30:** 727	**BMI 18.5-<25:** 75.3y +/- 9.5<br>**≥25-<30:** 74.5y +/- 9.5<br>**≥30:** 72.5y +/- 9.2	1070 days [750-1110] (overall, NR for BMI subgroups)	***Ischemic stroke:***<br>BMI ≥30 vs <30:<br>1.17 [0.62-2.22]<br><br>**Incidence rate:**<br>***Stroke:***<br>BMI <25: 0.85%/y<br>BMI 25-<30: 0.74%/y<br>BMI ≥30: 0.79%/y<br>***Systemic embolism:***<br>BMI <25: 0.85%/y<br>BMI 25-<30: 0.84%/y<br>BMI ≥30: 0.79%/y	BMI ≥30 vs <30:<br>0.88 [0.61-1.28]<br><br>**Incidence rate:**<br>BMI <25: 2.18%/y<br>BMI 25-<30: 2.91%/y<br>BMI ≥30: 2.24%/y	NR	NR	BMI ≥30 vs <30:<br>0.94 [0.71-1.25]<br><br>**Incidence rate:**<br>BMI <25: 4.84%/y<br>BMI 25-<30: 4.55%/y<br>BMI ≥30: 3.85%/y
Lucijanic et al. 2020[32]	Observational retrospective single centre cohort study (Croatia)	NOAC-treated AF patients (55% dabi, 22% riva, 23% api), categorized according to BMI <30, 30-35 and ≥35 kg/m².	**BMI <30:** 233;<br>**30-35:** 71;<br>**≥35:** 21	**BMI <30:** 71y [63-77.3];<br>**30-35:** 67y [60.3-75];<br>**≥35:** 69y [63.5-72.8]	33 months (overall, NR for BMI subgroups)	***Time to stroke/SE:***<br>BMI ≥30 vs <30:<br>*2.19 [1.00-4.76], p-value 0.048*	***Time to major bleeding:***<br>BMI ≥30 vs <30:<br>*2.78 [1.31-5.91], p-value 0.008*	NR	NR	BMI ≥30 vs <30:<br>1.49 [0.69-3.2]
Li et al. 2020[33]	Observational retrospective multicentre cohort study (China)	AF patients ≥60 years old using dabigatran 110 mg included in the MISSION-AF study, stratified according to the BMI tertiles (<22.5, 22.5-25.3, >25.3 kg/m²) and BMI classification (<25, ≥25-<30, ≥30 kg/m²).	**BMI <22.5:** 161;<br>**22.5-25.3:** 172;<br>**>25.3:** 176;<br>**<25:** 315;<br>**≥25-<30:** 168;<br>**≥30:** 26	**BMI <22.5:** 70.33y +/- 6.71;<br>**22.5-25.3:** 70.42y +/- 6.69;<br>**>25.3:** 69.32y +/- 6.59	6 months (overall, NR for BMI subgroups)	NR	***Any (major or minor) bleeding:***<br>BMI 22.5-25.3 vs <22.5:<br>*2.71 [1.02-7.07]*<br>BMI >25.3 vs <22.5:<br>*3.25 [1.21-8.70]*<br>BMI 25-<30 vs 18.5-<25:<br>*2.12 [1.04-4.31]*<br>BMI ≥30 vs 18.5-<25:<br>*3.67 [1.20-11.26]*	NR	NR	NR

Okumura et al. 2020[34]	Observational prospective multicentre cohort study (Japan)	Pooled data of AF patients from 5 major Japanese AF registries (J-RHYTHM Registry, Fushimi AF registry, Shinken Database, Keio Study and Hokuriku-Plus AF Registry); 26% no OAC, 64% warf, 10% NOAC; identifying predictors for ischemic stroke	**Overall:** 12 289 (3197 no OAC, 7886 warf, 1206 NOAC); **BMI <18.5:** 895	70.2y +/- 11.0	649 +/- 181 days	***Ischemic stroke:***<br>BMI <18.5 vs ≥18.5:<br>*1.55 [1.05-2.29]*	NR	NR	NR	NR
Patti et al. 2020[35]	Two observational prospective multicentre cohort studies (9 European countries)	OAC-naïve AF patients included in two prospective European registries (PREFER in AF and PREFER in AF PROLONGATION), categorized into quartiles of BMI at baseline (Q1: 14.0-24.6; Q2: 24.7-27.2; Q3: 27.3-30.5; Q4: 30.6-71.4 kg/m²) and with (n = 8288, 88.8%) vs without (n = 1042, 11.2%) OAC. Industry-sponsored.	***OAC-treated:*** **BMI 14.0-24.6:** 2026; **24.7-27.2:** 2011; **27.3-30.5:** 2130; **30.6-71.4 kg/m²:** 2120	**BMI 14.0-24.6:** 74.1y +/- 10.5; **24.7-27.2:** 72.4y +/- 10.2; **27.3-30.5:** 71.8y +/- 9.5; **30.6-71.4 kg/m²:** 69.3y +/- 9.7 (overall, NR for OAC-treated subgroup)	12 months +/- 2	***OAC-treated:***<br>***Stroke/TIA/SE/VTE: Event rate:***<br>BMI 14.0-24.6:<br>2.12%/y;<br>BMI 24.7-27.2:<br>2.54%/y;<br>BMI 27.3-30.5:<br>2.11%/y;<br>BMI 30.6-71.4:<br>1.70%/y<br><br>(p-value trend 0.32)	***OAC-treated:***<br>***Event rate:***<br>BMI 14.0-24.6:<br>2.76%/y;<br>BMI 24.7-27.2:<br>2.64%/y;<br>BMI 27.3-30.5:<br>1.83%/y;<br>BMI 30.6-71.4:<br>2.55%/y<br><br>(p-value trend 0.20)	NR	NR	NR
Kaplan et al. 2020[36*]	Observational retrospective nationwide cohort study (USA)	AF patients included from the Northwestern Medicine Enterprise Data Warehouse (Illinois) between 2010-2017, treated with NOACs (44% api, 36% riva, 0.1% edo, 20% dabi), categorized according to BMI 18.5-<25, ≥25-<30, ≥30-<35 and ≥35 kg/m²	**BMI 18.5-<25:** 1720; **≥25-<30:** 2804; **≥30-<35:** 1697; **≥35:** 1421	**BMI 18.5-<25:** 72y +/- 13; **≥25-<30:** 69y +/- 12; **≥30-<35:** 67y +/- 12; **≥35:** 64y +/- 11	3.8 years [2.2-6.0] (overall, NR for BMI subgroups)	BMI 25-<30 vs 18.5-<25:<br>1.25 [0.58-2.70]<br>BMI ≥30-<35 vs 18.5-<25:<br>1.22 [0.52-2.87]<br>BMI ≥35 vs 18.5-<25:<br>0.68 [0.23-2.01]	NR	BMI 25-<30 vs 18.5-<25:<br>0.69 [0.42-1.15]<br>BMI ≥30-<35 vs 18.5-<25:<br>0.64 [0.35-1.16]<br>BMI ≥35 vs 18.5-<25:<br>0.66 [0.35-1.24]	NR	NR
Lee et al. 2021[37*]	Observational retrospective nationwide cohort study (Korea)	AF patients included from the Korean National Health Insurance Service (NHIS) database	**BMI <18.5:** 1154; **18.5-<23:** 11918; **≥23-<25:**	**BMI <18.5:** 76.6y +/- 10.1; **18.5-<23:** 73.3y +/- 9.9; **≥23-<25:**	0.6 years [0.2-1.2]	***Ischemic stroke***<br>BMI <18.5 vs 18.5-<23:<br>1.05 [0.69-1.59];	BMI <18.5 vs 18.5-<23:<br>0.50 [0.24-1.01];<br>BMI ≥23-<25 vs 18.5-<23:	BMI <18.5 vs 18.5-<23:<br>0.46 [0.11-1.89];<br>BMI ≥23-<25 vs 18.5-<23:	BMI <18.5 vs 18.5-<23:<br>0.50 [0.22-1.14];<br>BMI ≥23-<25 vs 18.5-<23:	BMI <18.5 vs 18.5-<23:<br>*1.67 [1.36-2.05];*<br>BMI ≥23-<25 vs 18.5-<23:

		between 2015-2017, newly treated with OACs (22% warf, 78% NOAC, 36% riva, 21% dabi, 25% api, 18% edo, 54% reduced dose NOAC), categorized according to BMI <18.5, 18.5-<23, ≥23-<25, ≥25-<30 and ≥30 kg/m². Events and incidence rate described, allowing reclassification of BMI subgroups.	10589;<br>**≥25-<30:** 16779;<br>**≥30 kg/m²:** 2733;<br><br>**Reclassified BMI subgroup: 18.5-<25: 22507**	71.5y +/- 9.5;<br>**≥25-<30:** 70.1y +/- 9.7;<br>**≥30 kg/m²:** 67.6y +/- 10.8		BMI ≥23-<25 vs 18.5-<23:<br>1.14 [0.95-1.37];<br>BMI ≥25-<30 vs 18.5-<23:<br>0.95 [0.79-1.23];<br>BMI ≥30 vs 18.5-<23:<br>**0.68 [0.47-0.98]**<br><br>Per 5 kg/m² increase in BMI:<br>**0.89 [0.80-0.99]**<br><br>***Number of events:***<br>**BMI <18.5:** 25<br>**BMI 18.5-<23:** 237<br>**BMI ≥23-<25:** 229<br>**BMI 18.5-<25: 466**<br>**BMI ≥25-<30:** 292<br>**BMI ≥30:** 33	**0.78 [0.62-0.995];**<br>BMI ≥25-<30 vs 18.5-<23:<br>**0.64 [0.51-0.80];**<br>BMI ≥30 vs 18.5-<23:<br>0.70 [0.46-1.09]<br><br>Per 5 kg/m² increase in BMI:<br>**0.79 [0.69-0.92]**<br><br>***Number of events:***<br>**BMI <18.5:** 8<br>**BMI 18.5-<23:** 169<br>**BMI ≥23-<25:** 114<br>**BMI 18.5-<25: 283**<br>**BMI ≥25-<30:** 141<br>**BMI ≥30:** 24	0.90 [0.59-1.38];<br>BMI ≥25-<30 vs 18.5-<23:<br>0.75 [0.50-1.13];<br>BMI ≥30 vs 18.5-<23:<br>0.49 [0.19-1.25]<br><br>Per 5 kg/m² increase in BMI:<br>0.82 [0.63-1.06]<br><br>***Number of events:***<br>**BMI <18.5:** 2<br>**BMI 18.5-<23:** 48<br>**BMI ≥23-<25:** 38<br>**BMI 18.5-<25: 86**<br>**BMI ≥25-<30:** 49<br>**BMI ≥30:** 5	**0.74 [0.56-0.99];**<br>BMI ≥25-<30 vs 18.5-<23:<br>**0.60 [0.45-0.79];**<br>BMI ≥30 vs 18.5-<23:<br>0.81 [0.49-1.33]<br><br>Per 5 kg/m² increase in BMI:<br>**0.79 [0.66-0.94]**<br><br>***Number of events:***<br>**BMI <18.5:** 6<br>**BMI 18.5-<23:** 121<br>**BMI ≥23-<25:** 76<br>**BMI 18.5-<25: 197**<br>**BMI ≥25-<30:** 92<br>**BMI ≥30:** 19	**0.73 [0.64-0.84];**<br>BMI ≥25-<30 vs 18.5-<23:<br>**0.65 [0.57-0.75];**<br>BMI ≥30 vs 18.5-<23:<br>**0.55 [0.41-0.75]**<br><br>Per 5 kg/m² increase in BMI:<br>**0.66 [0.61-0.72]**<br><br>***Number of events:***<br>**BMI <18.5:** 114<br>**BMI 18.5-<23:** 513<br>**BMI ≥23-<25:** 309<br>**BMI 18.5-<25: 822**<br>**BMI ≥25-<30:** 389<br>**BMI ≥30:** 47

eTable 2: Overview of included studies investigating the impact of underweight (BMI <18.5 kg/m²) and (morbid) obesity (BMI ≥30/40 kg/m²) on outcomes of anticoagulated atrial fibrillation (AF) patients compared to normal BMI (18.5-<25 kg/m²) AF patients.

Bold: significantly lower risk; *Italic*: significantly higher risk.

*: included in meta-analysis.

AF: atrial fibrillation; Api: apixaban; BMI: body mass index; CI: confidence interval; CRNMB: clinically relevant non-major bleeding; Dabi: dabigatran; Dabi 110: dabigatran 110 mg (reduced dose); Dabi 150: dabigatran 150 mg (standard dose); Edo 60/30: edoxaban 60 mg (standard dose) and 30 mg (reduced dose); Edo: edoxaban; FDA: the Food and Drug Administration (USA); HR: hazard ratio; IQR: interquartile range; NOAC: non-vitamin K antagonist oral anticoagulant; NR: not reported; OAC: oral anticoagulant; OR: odds ratio; RCT: randomized controlled trial; Riva: rivaroxaban; RR: risk ratio; SD: standard deviation; SmPC: Summary of Product Characteristics; Stroke/SE: stroke/systemic embolism; Stroke/SE/MI: stroke/systemic embolism/myocardial infarction; USA: United States of America; VKA: vitamin K antagonist; Vs: versus; Warf: warfarin; y: year.

eTable 3: Assessment of bias within studies

A)

Reference: Sandhu et al. 2016[12]					
**Criteria**		**Yes (2)**	**Partial (1)**	**No (0)**	**N/A**
**1**	Question / objective sufficiently described?	2			
**2**	Study design evident and appropriate?	2			
**3**	Method of subject/comparison group selection or source of information/input variables described and appropriate?	2			
**4**	Subject and comparison group (if applicable) characteristics sufficiently described?		1 (randomized study with extensive description of baseline characteristics in normal BMI, overweight and obese AF patients, but not reported for morbidly obese AF patients)		
**5**	If interventional and random allocation was possible, was it reported?	2			
**6**	If interventional and blinding of investigators was possible, was it reported?	2			
**7**	If interventional and blinding of subjects was possible, was it reported?	2			
**8**	Outcome and (if applicable) exposure measure(s) well defined and robust to measurement / misclassification bias? Means of assessment reported?	2			
**9**	Sample size appropriate?	2			
**10**	Analytic methods described/justified and appropriate?	2			
**11**	Some estimate of variance is reported for the main results?	2			
**12**	Controlling for confounding?	2			
**13**	Results reported in sufficient detail?	2			
**14**	Conclusion supported by the results?	2			
Total score: 27/28 (96.4%)					

B)

Reference: Balla et al. 2017[13]					
**Criteria**		**Yes (2)**	**Partial (1)**	**No (0)**	**N/A**
**1**	Question / objective sufficiently described?	2			
**2**	Study design evident and appropriate?	2			
**3**	Method of subject/comparison group selection or source of information/input variables described and appropriate?	2			
**4**	Subject and comparison group (if applicable) characteristics sufficiently described?	2			
**5**	If interventional and random allocation was possible, was it reported?	2			
**6**	If interventional and blinding of investigators was possible, was it reported?	2			
**7**	If interventional and blinding of subjects was possible, was it reported?	2			
**8**	Outcome and (if applicable) exposure measure(s) well defined and robust to measurement / misclassification bias? Means of assessment reported?	2			
**9**	Sample size appropriate?	2			
**10**	Analytic methods described/justified and appropriate?	2			
**11**	Some estimate of variance is reported for the main results?	2			
**12**	Controlling for confounding?	2			
**13**	Results reported in sufficient detail?	2			
**14**	Conclusion supported by the results?	2			
Total score: 28/28 (100%)					

C)

Reference: Boriani et al. 2019[14]					
**Criteria**		**Yes (2)**	**Partial (1)**	**No (0)**	**N/A**
**1**	Question / objective sufficiently described?	2			
**2**	Study design evident and appropriate?	2			
**3**	Method of subject/comparison group selection or source of information/input variables described and appropriate?	2			
**4**	Subject and comparison group (if applicable) characteristics sufficiently described?	2			
**5**	If interventional and random allocation was possible, was it reported?	2			
**6**	If interventional and blinding of investigators was possible, was it reported?	2			
**7**	If interventional and blinding of subjects was possible, was it reported?	2			
**8**	Outcome and (if applicable) exposure measure(s) well defined and robust to measurement / misclassification bias? Means of assessment reported?	2			
**9**	Sample size appropriate?	2			
**10**	Analytic methods described/justified and appropriate?	2			
**11**	Some estimate of variance is reported for the main results?	2			
**12**	Controlling for confounding?	2			
**13**	Results reported in sufficient detail?	2			
**14**	Conclusion supported by the results?	2			
Total score: 28/28 (100%)					

D)

Reference: Boehringer Ingelheim, 2010[15] (regulatory submission for drug approval to the FDA, based on results from the RE-LY trial, described by Connolly et al. 2009[1])					
**Criteria**		**Yes (2)**	**Partial (1)**	**No (0)**	**N/A**
**1**	Question / objective sufficiently described?	2			
**2**	Study design evident and appropriate?	2			
**3**	Method of subject/comparison group selection or source of information/input variables described and appropriate?	2			
**4**	Subject and comparison group (if applicable) characteristics sufficiently described?			0 (pre-specified subgroup analysis, no description of baseline characteristics in BMI subgroups)	
**5**	If interventional and random allocation was possible, was it reported?	2			
**6**	If interventional and blinding of investigators was possible, was it reported?	2			
**7**	If interventional and blinding of subjects was possible, was it reported?	2			
**8**	Outcome and (if applicable) exposure measure(s) well defined and robust to measurement / misclassification bias? Means of assessment reported?	2			
**9**	Sample size appropriate?	2			
**10**	Analytic methods described/justified and appropriate?	2			
**11**	Some estimate of variance is reported for the main results?		1 (HR and 95%CI reported for major bleeding, but not stroke/SE (only incidence rates))		
**12**	Controlling for confounding?		1 (randomized study, but no specific report of any adjustments for potential confounders in subgroup analysis)		
**13**	Results reported in sufficient detail?		1 (pre-specified subgroup analysis with event rates in BMI subgroups, but no extra details supplied)		
**14**	Conclusion supported by the results?	2			
Total score: 23/28 (82.1%)					

E)

Reference: Park et al. 2017[26]						
**Criteria**		**Yes (2)**	**Partial (1)**	**No (0)**	**N/A**
**1**	Question / objective sufficiently described?	2			
**2**	Study design evident and appropriate?	2			
**3**	Method of subject/comparison group selection or source of information/input variables described and appropriate?	2			
**4**	Subject and comparison group (if applicable) characteristics sufficiently described?	2			
**5**	If interventional and random allocation was possible, was it reported?				N/A
**6**	If interventional and blinding of investigators was possible, was it reported?				N/A
**7**	If interventional and blinding of subjects was possible, was it reported?				N/A
**8**	Outcome and (if applicable) exposure measure(s) well defined and robust to measurement / misclassification bias? Means of assessment reported?		1 (outcome measures retrospectively retrieved from the electronic medical records, which are prone to misclassification bias)		
**9**	Sample size appropriate?		1 (smaller sample size in underweight subgroup (n = 62) as compared to normal BMI (n = 753) and overweight (n = 538) subgroups)		
**10**	Analytic methods described/justified and appropriate?	2			
**11**	Some estimate of variance is reported for the main results?	2			
**12**	Controlling for confounding?		1 (Cox proportional hazard model adjusted for age, sex, comorbidities and NOAC dosage; however, due to retrospective review of electronic medical records, unmeasured confounders and biases may still be present)		
**13**	Results reported in sufficient detail?	2			
**14**	Conclusion supported by the results?	2			
Total score: 19/22 (86.4%)					

F)

Reference: Murakawa et al. 2020[30]					
**Criteria**		**Yes (2)**	**Partial (1)**	**No (0)**	**N/A**
**1**	Question / objective sufficiently described?	2			
**2**	Study design evident and appropriate?	2			
**3**	Method of subject/comparison group selection or source of information/input variables described and appropriate?	2			
**4**	Subject and comparison group (if applicable) characteristics sufficiently described?	2			
**5**	If interventional and random allocation was possible, was it reported?				N/A
**6**	If interventional and blinding of investigators was possible, was it reported?				N/A
**7**	If interventional and blinding of subjects was possible, was it reported?				N/A
**8**	Outcome and (if applicable) exposure measure(s) well defined and robust to measurement / misclassification bias? Means of assessment reported?	2			
**9**	Sample size appropriate?		1 (smaller sample size in obese subgroup (n = 499) as compared to normal BMI (n = 4410) and overweight (n = 2167) subgroups)		
**10**	Analytic methods described/justified and appropriate?	2			
**11**	Some estimate of variance is reported for the main results?	2			
**12**	Controlling for confounding?	2			
**13**	Results reported in sufficient detail?	2			
**14**	Conclusion supported by the results?	2			
Total score: 21/22 (95.5%)					

G)

Reference: Bertomeu-Gonzalez et al. 2020[31]					
**Criteria**		**Yes (2)**	**Partial (1)**	**No (0)**	**N/A**
**1**	Question / objective sufficiently described?	2			
**2**	Study design evident and appropriate?	2			
**3**	Method of subject/comparison group selection or source of information/input variables described and appropriate?	2			
**4**	Subject and comparison group (if applicable) characteristics sufficiently described?	2			
**5**	If interventional and random allocation was possible, was it reported?				N/A
**6**	If interventional and blinding of investigators was possible, was it reported?				N/A
**7**	If interventional and blinding of subjects was possible, was it reported?				N/A
**8**	Outcome and (if applicable) exposure measure(s) well defined and robust to measurement / misclassification bias? Means of assessment reported?	2			
**9**	Sample size appropriate?		1 (limited sample sizes: 358 normal BMI AF patients, 871 overweight patients, 727 obese patients).		
**10**	Analytic methods described/justified and appropriate?	2			
**11**	Some estimate of variance is reported for the main results?	2			
**12**	Controlling for confounding?	2			
**13**	Results reported in sufficient detail?	2			
**14**	Conclusion supported by the results?	2			
Total score: 21/22 (95.5%)					

H)

Reference: Kaplan et al. 2020[36]					
**Criteria**		**Yes (2)**	**Partial (1)**	**No (0)**	**N/A**
**1**	Question / objective sufficiently described?	2			
**2**	Study design evident and appropriate?	2			
**3**	Method of subject/comparison group selection or source of information/input variables described and appropriate?	2			
**4**	Subject and comparison group (if applicable) characteristics sufficiently described?	2			
**5**	If interventional and random allocation was possible, was it reported?				N/A
**6**	If interventional and blinding of investigators was possible, was it reported?				N/A
**7**	If interventional and blinding of subjects was possible, was it reported?				N/A
**8**	Outcome and (if applicable) exposure measure(s) well defined and robust to measurement / misclassification bias? Means of assessment reported?		1 (outcome measures retrospectively assessed in administrative healthcare claims database using ICD-codes, which are prone to misclassification bias)		
**9**	Sample size appropriate?	2			
**10**	Analytic methods described/justified and appropriate?	2			
**11**	Some estimate of variance is reported for the main results?	2			
**12**	Controlling for confounding?		1 (Cox proportional hazard model only adjusted for CHA_2DS_2-VASc score, additional inverse probability of treatment weighting analysis adjusted for age, sex, race, ethnicity and CHA_2DS_2-VASc score; however, due to retrospective use of administrative healthcare claims database, unmeasured confounders and biases may still be present)		
**13**	Results reported in sufficient detail?	2			
**14**	Conclusion supported by the results?	2			
Total score: 20/22 (90.9%)					

I)

Reference: Lee et al. 2021[37]					
**Criteria**		**Yes (2)**	**Partial (1)**	**No (0)**	**N/A**
**1**	Question / objective sufficiently described?	2			
**2**	Study design evident and appropriate?	2			
**3**	Method of subject/comparison group selection or source of information/input variables described and appropriate?	2			
**4**	Subject and comparison group (if applicable) characteristics sufficiently described?	2			
**5**	If interventional and random allocation was possible, was it reported?				N/A
**6**	If interventional and blinding of investigators was possible, was it reported?				N/A
**7**	If interventional and blinding of subjects was possible, was it reported?				N/A
**8**	Outcome and (if applicable) exposure measure(s) well defined and robust to measurement / misclassification bias? Means of assessment reported?		1 (outcome measures retrospectively assessed in administrative healthcare claims database using ICD-codes, which are prone to misclassification bias)		
**9**	Sample size appropriate?	2			
**10**	Analytic methods described/justified and appropriate?	2			
**11**	Some estimate of variance is reported for the main results?	2			
**12**	Controlling for confounding?		1 (Cox proportional hazard model adequately adjusted for age, sex, CHA_2DS_2-VASc score, comorbidities, renal function, antiplatelet use and OAC treatment; however, due to retrospective use of administrative healthcare claims database, unmeasured confounders and biases may still be present)		
**13**	Results reported in sufficient detail?	2			
**14**	Conclusion supported by the results?	2			
Total score: 20/22 (90.9%)					

<u>eTable 3:</u> Assessment of bias within studies included in the meta-analysis regarding the impact of body mass index on outcomes (stroke or systemic embolism, major bleeding, all-cause mortality) in atrial fibrillation patients (**A-D:** 4 post hoc analyses or regulatory submission for drug approval of phase III randomized controlled trials; **E-I:** 5 longitudinal observational cohort studies), using the quality assessment tool 'QUALSYST' from the "Standard Quality Assessment Criteria for Evaluating Primary Research Papers from a Variety of Fields".[38] With this tool, 14 items of each quantitative study were scored on the study and outcome levels depending on the degree to which the specific criteria were met or reported ("yes" = 2, "partial" = 1, "no" = 0). Items not applicable to a particular study design were marked "n/a" and were excluded from the calculation of the summary score. A percentage was calculated for each paper by dividing the total sum score obtained across rated items by the total possible score. Studies were included if scoring ≥75%.

AF: atrial fibrillation; CI: confidence interval; FDA: the U.S. Food and Drug Administration; HR: hazard ratio; Stroke/SE: stroke or systemic embolism.

eTable 4: PRISMA 2009 checklist

Section/topic	#	Checklist item	Reported on page #
**TITLE**			
Title	1	Identify the report as a systematic review, meta-analysis, or both.	1
**ABSTRACT**			
Structured summary	2	Provide a structured summary including, as applicable: background; objectives; data sources; study eligibility criteria, participants, and interventions; study appraisal and synthesis methods; results; limitations; conclusions and implications of key findings; systematic review registration number.	2
**INTRODUCTION**			
Rationale	3	Describe the rationale for the review in the context of what is already known.	3
Objectives	4	Provide an explicit statement of questions being addressed with reference to participants, interventions, comparisons, outcomes, and study design (PICOS).	3,4
**METHODS**			
Protocol and registration	5	Indicate if a review protocol exists, if and where it can be accessed (e.g., Web address), and, if available, provide registration information including registration number.	Not applicable
Eligibility criteria	6	Specify study characteristics (e.g., PICOS, length of follow-up) and report characteristics (e.g., years considered, language, publication status) used as criteria for eligibility, giving rationale.	4
Information sources	7	Describe all information sources (e.g., databases with dates of coverage, contact with study authors to identify additional studies) in the search and date last searched.	4
Search	8	Present full electronic search strategy for at least one database, including any limits used, such that it could be repeated.	eTable 1
Study selection	9	State the process for selecting studies (i.e., screening, eligibility, included in systematic review, and, if applicable, included in the meta-analysis).	4, 5
Data collection process	10	Describe method of data extraction from reports (e.g., piloted forms, independently, in duplicate) and any processes for obtaining and confirming data from investigators.	4, 5
Data items	11	List and define all variables for which data were sought (e.g., PICOS, funding sources) and any assumptions and simplifications made.	4, 5
Risk of bias in individual studies	12	Describe methods used for assessing risk of bias of individual studies (including specification of whether this was done at the study or outcome level), and how this information is to be used in any data synthesis.	5
Summary measures	13	State the principal summary measures (e.g., risk ratio, difference in means).	5
Synthesis of results	14	Describe the methods of handling data and combining results of studies, if done, including measures of consistency (e.g., I^2) for each meta-analysis.	5

Section/topic	#	Checklist item	Reported on page #
Risk of bias across studies	15	Specify any assessment of risk of bias that may affect the cumulative evidence (e.g., publication bias, selective reporting within studies).	5
Additional analyses	16	Describe methods of additional analyses (e.g., sensitivity or subgroup analyses, meta-regression), if done, indicating which were pre-specified.	4, 5, 10, 11
**RESULTS**			
Study selection	17	Give numbers of studies screened, assessed for eligibility, and included in the review, with reasons for exclusions at each stage, ideally with a flow diagram.	5 + Figure 1
Study characteristics	18	For each study, present characteristics for which data were extracted (e.g., study size, PICOS, follow-up period) and provide the citations.	eTable 2
Risk of bias within studies	19	Present data on risk of bias of each study and, if available, any outcome level assessment (see item 12).	eTable 3
Results of individual studies	20	For all outcomes considered (benefits or harms), present, for each study: (a) simple summary data for each intervention group (b) effect estimates and confidence intervals, ideally with a forest plot.	eTable 2
Synthesis of results	21	Present results of each meta-analysis done, including confidence intervals and measures of consistency.	10-11; Figure 2-4 + eFigure 1-3
Risk of bias across studies	22	Present results of any assessment of risk of bias across studies (see Item 15).	11; eFigure 7
Additional analysis	23	Give results of additional analyses, if done (e.g., sensitivity or subgroup analyses, meta-regression [see Item 16]).	10, 11; eFigure 4-6
**DISCUSSION**			
Summary of evidence	24	Summarize the main findings including the strength of evidence for each main outcome; consider their relevance to key groups (e.g., healthcare providers, users, and policy makers).	12
Limitations	25	Discuss limitations at study and outcome level (e.g., risk of bias), and at review-level (e.g., incomplete retrieval of identified research, reporting bias).	14-15
Conclusions	26	Provide a general interpretation of the results in the context of other evidence, and implications for future research.	12
**FUNDING**			
Funding	27	Describe sources of funding for the systematic review and other support (e.g., supply of data); role of funders for the systematic review.	1

From: Moher D, Liberati A, Tetzlaff J, Altman DG, The PRISMA Group (2009). Preferred Reporting Items for Systematic Reviews and Meta-Analyses: The PRISMA Statement. PLoS Med 6(7): e1000097. doi:10.1371/journal.pmed1000097

For more information, visit: **www.prisma-statement.org**.

Supplemental figures

eFigure 1: Impact of overweight on AF-related outcomes

A)

Study or Subgroup	Overweight Events	Overweight Total	Normal BMI Events	Normal BMI Total	Weight	Risk Ratio M-H, Random, 95% CI	Year
**2.1.1 RCTs**							
Boehringer Ingelheim (RE-LY)	196	7111	181	4697	20.6%	0.72 [0.59, 0.87]	2010
Sandhu et al. (ARISTOTLE)	174	6671	142	4038	17.1%	0.74 [0.60, 0.92]	2016
Balla et al. (ROCKET AF)	226	5535	166	3289	21.2%	0.81 [0.67, 0.98]	2017
Boriani et al. (ENGAGE AF-TIMI 48)	407	7903	273	4491	36.7%	0.85 [0.73, 0.98]	2019
**Subtotal (95% CI)**		**27220**		**16515**	**95.6%**	**0.79 [0.72, 0.87]**	
Total events	1003		762				
Heterogeneity: Tau² = 0.00; Chi² = 2.19, df = 3 (P = 0.53); I² = 0%							
Test for overall effect: Z = 5.02 (P < 0.00001)							
**2.1.2 Observational studies**							
Park et al.	1	538	9	753	0.2%	0.16 [0.02, 1.22]	2017
Bertomeu-Gonzalez et al.	41	871	18	358	2.8%	0.94 [0.55, 1.61]	2020
Kaplan et al.	19	2804	10	1720	1.4%	1.17 [0.54, 2.50]	2020
**Subtotal (95% CI)**		**4213**		**2831**	**4.4%**	**0.87 [0.46, 1.66]**	
Total events	61		37				
Heterogeneity: Tau² = 0.13; Chi² = 3.31, df = 2 (P = 0.19); I² = 40%							
Test for overall effect: Z = 0.42 (P = 0.67)							
**Total (95% CI)**		**31433**		**19346**	**100.0%**	**0.80 [0.73, 0.87]**	
Total events	1064		799				
Heterogeneity: Tau² = 0.00; Chi² = 5.93, df = 6 (P = 0.43); I² = 0%							
Test for overall effect: Z = 4.98 (P < 0.00001)							
Test for subgroup differences: Chi² = 0.09, df = 1 (P = 0.77), I² = 0%							

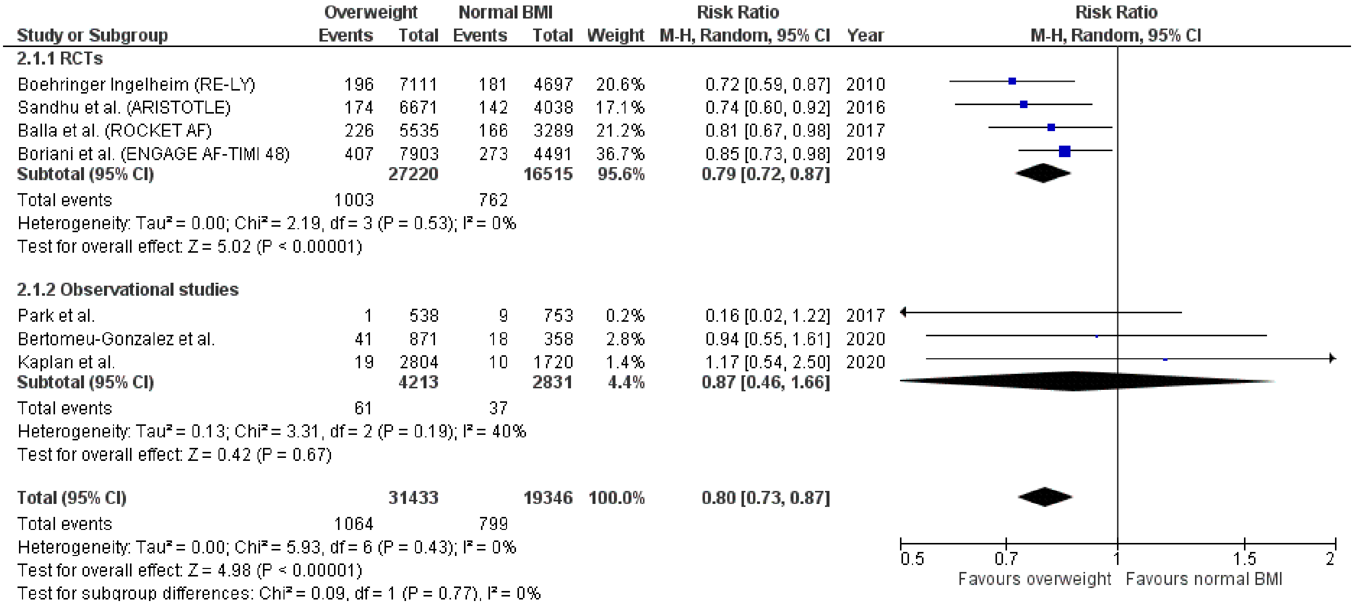

B)

Study or Subgroup	Overweight Events	Overweight Total	Normal BMI Events	Normal BMI Total	Weight	Risk Ratio M-H, Random, 95% CI	Year
**2.2.1 RCTs**							
Sandhu et al. (ARISTOTLE)	429	6671	397	4038	25.1%	0.65 [0.57, 0.75]	2016
Boriani et al. (ENGAGE AF-TIMI 48)	858	7903	629	4491	28.6%	0.78 [0.70, 0.85]	2019
**Subtotal (95% CI)**		**14574**		**8529**	**53.7%**	**0.72 [0.61, 0.85]**	
Total events	1287		1026				
Heterogeneity: Tau² = 0.01; Chi² = 4.21, df = 1 (P = 0.04); I² = 76%							
Test for overall effect: Z = 3.94 (P < 0.0001)							
**2.2.2 Observational studies**							
Park et al.	3	538	6	753	0.8%	0.70 [0.18, 2.79]	2017
Murakawa et al.	33	2167	72	4410	7.5%	0.93 [0.62, 1.40]	2020
Bertomeu-Gonzalez et al.	119	871	52	358	11.6%	0.94 [0.70, 1.27]	2020
Lee et al.	389	16779	822	22507	26.3%	0.63 [0.56, 0.71]	2021
**Subtotal (95% CI)**		**20355**		**28028**	**46.3%**	**0.78 [0.59, 1.04]**	
Total events	544		952				
Heterogeneity: Tau² = 0.04; Chi² = 8.03, df = 3 (P = 0.05); I² = 63%							
Test for overall effect: Z = 1.70 (P = 0.09)							
**Total (95% CI)**		**34929**		**36557**	**100.0%**	**0.73 [0.64, 0.83]**	
Total events	1831		1978				
Heterogeneity: Tau² = 0.01; Chi² = 13.17, df = 5 (P = 0.02); I² = 62%							
Test for overall effect: Z = 4.87 (P < 0.00001)							
Test for subgroup differences: Chi² = 0.30, df = 1 (P = 0.58), I² = 0%							

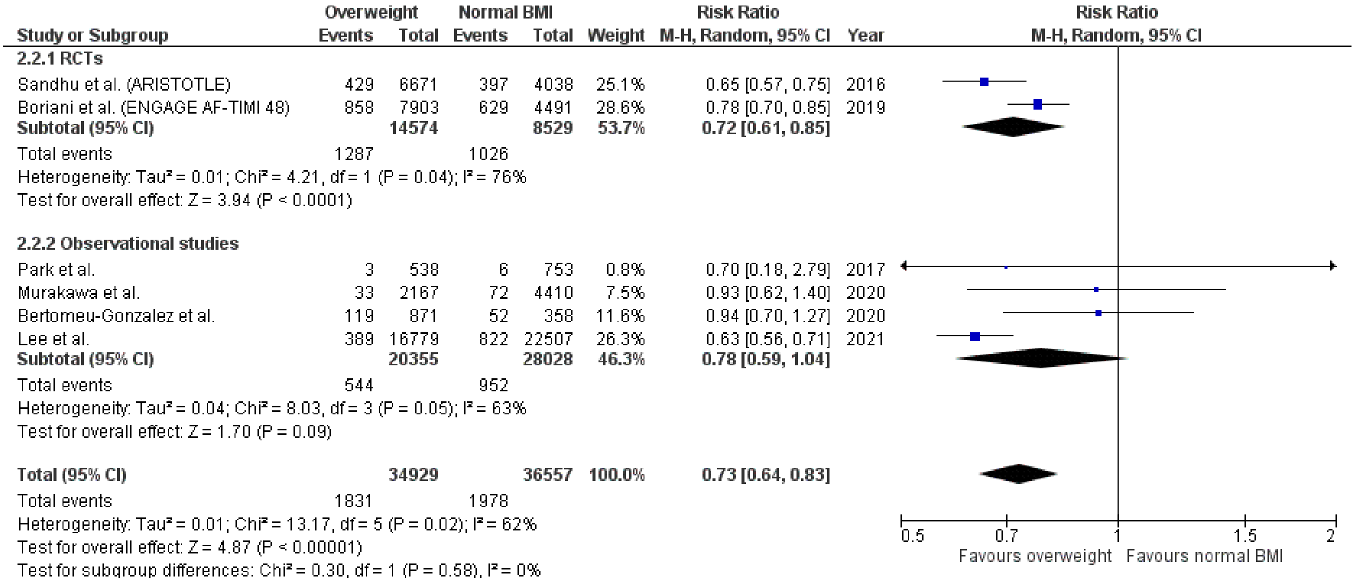

C)

Study or Subgroup	Overweight Events	Overweight Total	Normal BMI Events	Normal BMI Total	Weight	Risk Ratio M-H, Random, 95% CI	Year
**2.3.1 RCTs**							
Boehringer Ingelheim (RE-LY)	424	7111	344	4697	18.5%	0.81 [0.71, 0.93]	2010
Sandhu et al. (ARISTOTLE)	270	6618	217	3984	16.5%	0.75 [0.63, 0.89]	2016
Balla et al. (ROCKET AF)	314	5529	179	3274	16.3%	1.04 [0.87, 1.24]	2017
Boriani et al. (ENGAGE AF-TIMI 48)	472	7903	283	4491	18.2%	0.95 [0.82, 1.09]	2019
**Subtotal (95% CI)**		**27161**		**16446**	**69.4%**	**0.88 [0.77, 1.01]**	
Total events	1480		1023				
Heterogeneity: Tau² = 0.01; Chi² = 8.87, df = 3 (P = 0.03); I² = 66%							
Test for overall effect: Z = 1.87 (P = 0.06)							
**2.3.2 Observational studies**							
Park et al.	7	538	16	753	2.1%	0.61 [0.25, 1.48]	2017
Murakawa et al.	32	2167	70	4410	7.1%	0.93 [0.61, 1.41]	2020
Bertomeu-Gonzalez et al.	75	871	23	358	6.3%	1.34 [0.85, 2.10]	2020
Lee et al.	141	16779	283	22507	15.1%	0.67 [0.55, 0.82]	2021
**Subtotal (95% CI)**		**20355**		**28028**	**30.6%**	**0.86 [0.60, 1.23]**	
Total events	255		392				
Heterogeneity: Tau² = 0.08; Chi² = 8.78, df = 3 (P = 0.03); I² = 66%							
Test for overall effect: Z = 0.83 (P = 0.41)							
**Total (95% CI)**		**47516**		**44474**	**100.0%**	**0.86 [0.76, 0.99]**	
Total events	1735		1415				
Heterogeneity: Tau² = 0.02; Chi² = 19.58, df = 7 (P = 0.007); I² = 64%							
Test for overall effect: Z = 2.17 (P = 0.03)							
Test for subgroup differences: Chi² = 0.01, df = 1 (P = 0.91), I² = 0%							

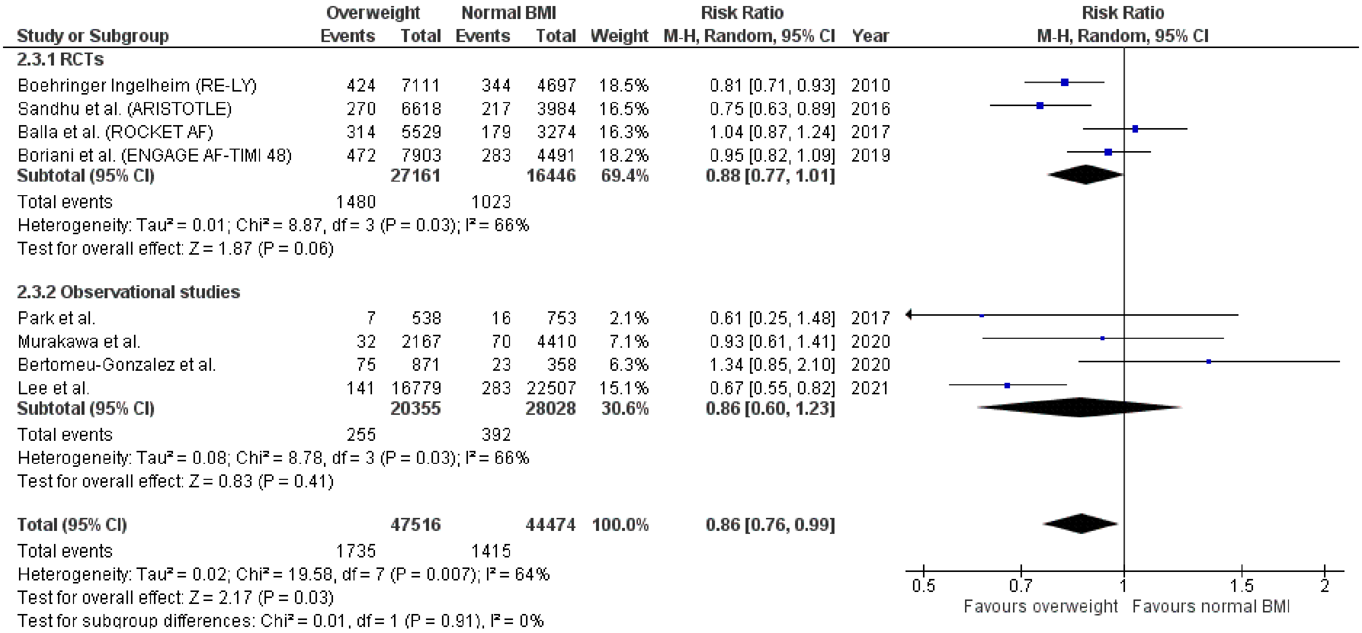

D)

Study or Subgroup	Overweight Events	Overweight Total	Normal BMI Events	Normal BMI Total	Weight	Risk Ratio M-H, Random, 95% CI	Year
**2.4.1 RCTs**							
Balla et al. (ROCKET AF)	34	5529	23	3274	22.8%	0.88 [0.52, 1.48]	2017
**Subtotal (95% CI)**		**5529**		**3274**	**22.8%**	**0.88 [0.52, 1.48]**	
Total events	34		23				
Heterogeneity: Not applicable							
Test for overall effect: Z = 0.49 (P = 0.62)							
**2.4.2 Observational studies**							
Kaplan et al.	31	2804	30	1720	25.5%	0.63 [0.39, 1.04]	2020
Lee et al.	49	16779	86	22507	51.7%	0.76 [0.54, 1.08]	2021
**Subtotal (95% CI)**		**19583**		**24227**	**77.2%**	**0.72 [0.54, 0.96]**	
Total events	80		116				
Heterogeneity: Tau² = 0.00; Chi² = 0.36, df = 1 (P = 0.55); I² = 0%							
Test for overall effect: Z = 2.26 (P = 0.02)							
**Total (95% CI)**		**25112**		**27501**	**100.0%**	**0.75 [0.58, 0.97]**	
Total events	114		139				
Heterogeneity: Tau² = 0.00; Chi² = 0.78, df = 2 (P = 0.68); I² = 0%							
Test for overall effect: Z = 2.22 (P = 0.03)							
Test for subgroup differences: Chi² = 0.42, df = 1 (P = 0.52), I² = 0%							

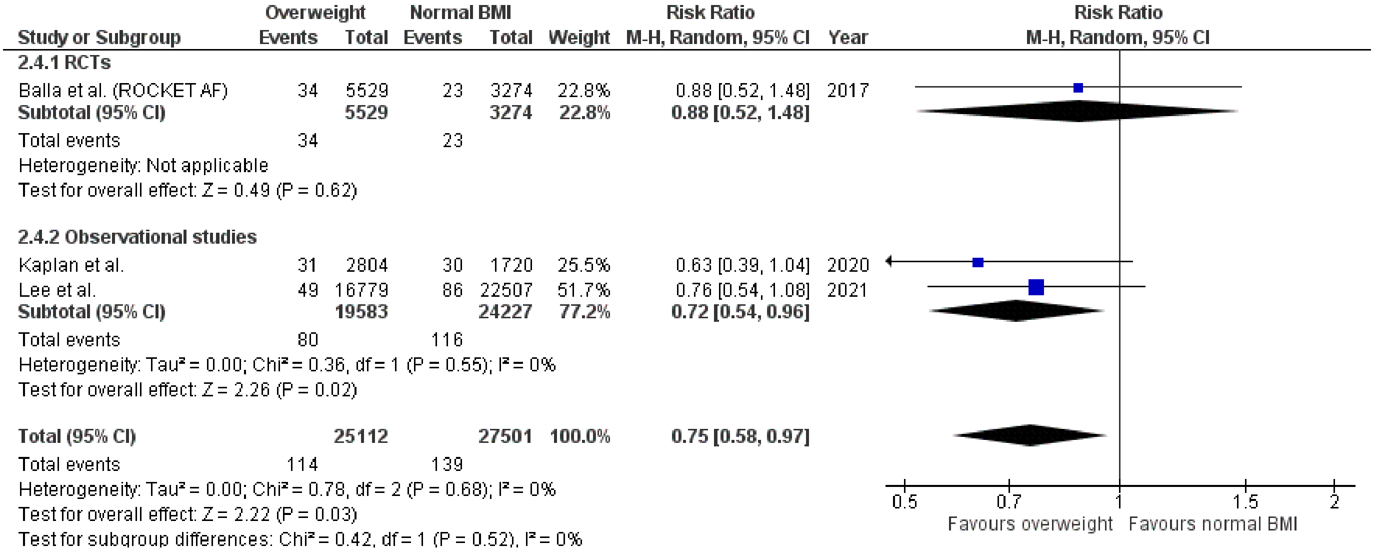

eFigure 1: Forest plot of the risk of **A)** stroke or systemic embolism, **B)** all-cause mortality, **C)** major bleeding, and **D)** intracranial bleeding for overweight (BMI 25-<30 kg/m²) versus normal BMI (18.5-<25 kg/m²) AF patients receiving anticoagulation, categorized according to randomized and observational studies.

AF: atrial fibrillation; ARISTOTLE: the Apixaban for Reduction in Stroke and Other Thromboembolic Events in Atrial Fibrillation trial; BMI: body mass index; CI: confidence interval; ENGAGE AF-TIMI 48: the Effective Anticoagulation with Factor Xa Next Generation in Atrial Fibrillation–Thrombolysis in Myocardial Infarction 48 trial; M-H: Mantel-Haenszel (statistical method); RCT: randomized controlled trial; RE-LY: the Randomized Evaluation of Long-Term Anticoagulation Therapy; ROCKET AF: the Rivaroxaban Once Daily Oral Direct Factor Xa Inhibition Compared with Vitamin K Antagonism for Prevention of Stroke and Embolism Trial in Atrial Fibrillation.

eFigure 2: Impact of class II obesity on AF-related outcomes

A)

Study or Subgroup	Class II obesity Events	Class II obesity Total	Normal BMI Events	Normal BMI Total	Weight	Risk Ratio M-H, Random, 95% CI	Year
**4.1.1 RCTs**							
Boehringer Ingelheim (RE-LY)	53	2241	181	4697	24.8%	0.61 [0.45, 0.83]	2010
Sandhu et al. (ARISTOTLE)	28	1764	142	4038	14.0%	0.45 [0.30, 0.67]	2016
Balla et al. (ROCKET AF)	55	1898	166	3289	25.2%	0.57 [0.43, 0.77]	2017
Boriani et al. (ENGAGE AF-TIMI 48)	70	2099	273	4491	34.1%	0.55 [0.42, 0.71]	2019
**Subtotal (95% CI)**		**8002**		**16515**	**98.0%**	**0.56 [0.48, 0.65]**	
Total events	206		762				
Heterogeneity: Tau² = 0.00; Chi² = 1.50, df = 3 (P = 0.68); I² = 0%							
Test for overall effect: Z = 7.60 (P < 0.00001)							
**4.1.2 Observational studies**							
Kaplan et al.	5	1421	10	1720	2.0%	0.61 [0.21, 1.77]	2020
**Subtotal (95% CI)**		**1421**		**1720**	**2.0%**	**0.61 [0.21, 1.77]**	
Total events	5		10				
Heterogeneity: Not applicable							
Test for overall effect: Z = 0.92 (P = 0.36)							
**Total (95% CI)**		**9423**		**18235**	**100.0%**	**0.56 [0.48, 0.65]**	
Total events	211		772				
Heterogeneity: Tau² = 0.00; Chi² = 1.53, df = 4 (P = 0.82); I² = 0%							
Test for overall effect: Z = 7.65 (P < 0.00001)							
Test for subgroup differences: Chi² = 0.02, df = 1 (P = 0.88), I² = 0%							

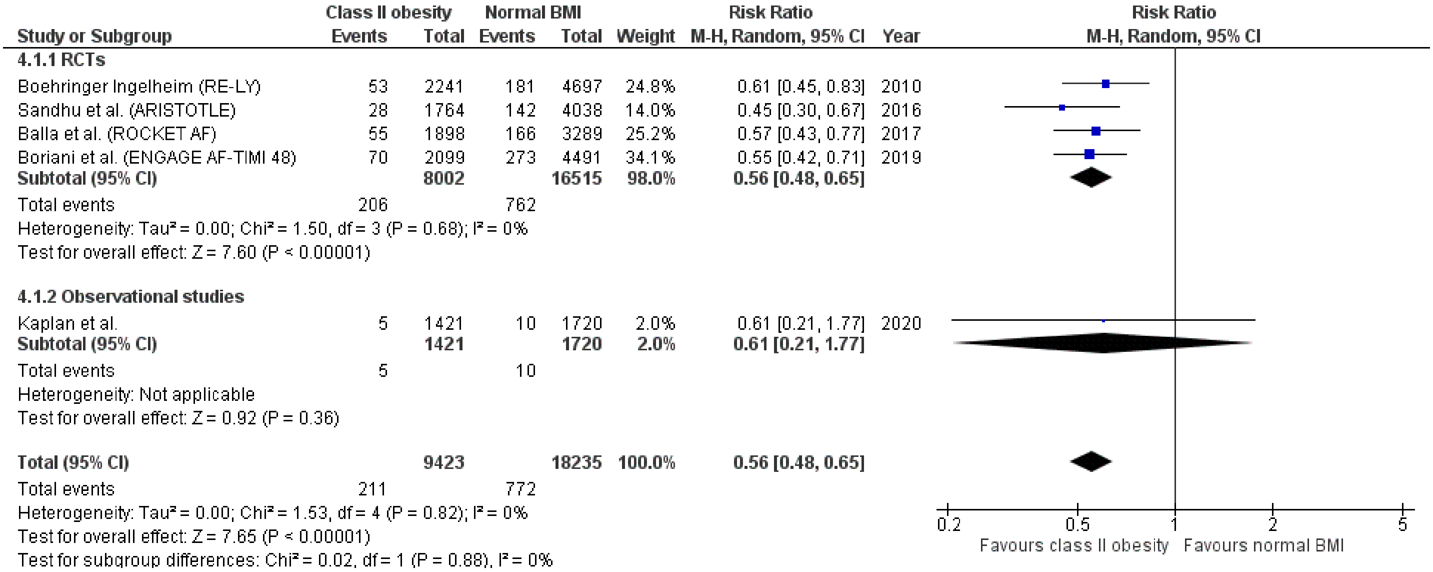

B)

Study or Subgroup	Class II obesity Events	Class II obesity Total	Normal BMI Events	Normal BMI Total	Weight	Risk Ratio M-H, Random, 95% CI	Year
Sandhu et al. (ARISTOTLE)	88	1764	397	4038	44.3%	0.51 [0.41, 0.63]	2016
Boriani et al. (ENGAGE AF-TIMI 48)	191	2099	629	4491	55.7%	0.65 [0.56, 0.76]	2019
**Total (95% CI)**		**3863**		**8529**	**100.0%**	**0.58 [0.46, 0.74]**	
Total events	279		1026				
Heterogeneity: Tau² = 0.02; Chi² = 3.20, df = 1 (P = 0.07); I² = 69%							
Test for overall effect: Z = 4.40 (P < 0.0001)							

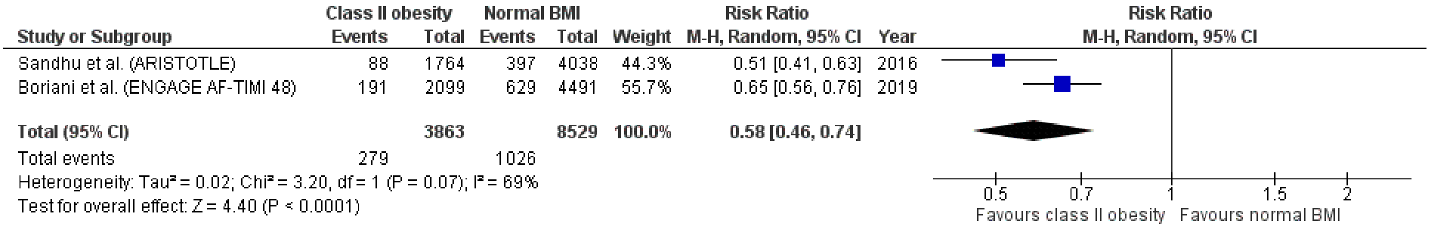

C)

Study or Subgroup	Class II obesity Events	Class II obesity Total	Normal BMI Events	Normal BMI Total	Weight	Risk Ratio M-H, Random, 95% CI	Year
Boehringer Ingelheim (RE-LY)	147	2241	344	4697	29.9%	0.90 [0.74, 1.08]	2010
Sandhu et al. (ARISTOTLE)	67	1745	217	3984	19.3%	0.70 [0.54, 0.92]	2016
Balla et al. (ROCKET AF)	106	1896	179	3274	23.1%	1.02 [0.81, 1.29]	2017
Boriani et al. (ENGAGE AF-TIMI 48)	130	2099	283	4491	27.6%	0.98 [0.80, 1.20]	2019
**Total (95% CI)**		**7981**		**16446**	**100.0%**	**0.90 [0.79, 1.04]**	
Total events	450		1023				
Heterogeneity: Tau² = 0.01; Chi² = 5.04, df = 3 (P = 0.17); I² = 40%							
Test for overall effect: Z = 1.38 (P = 0.17)							

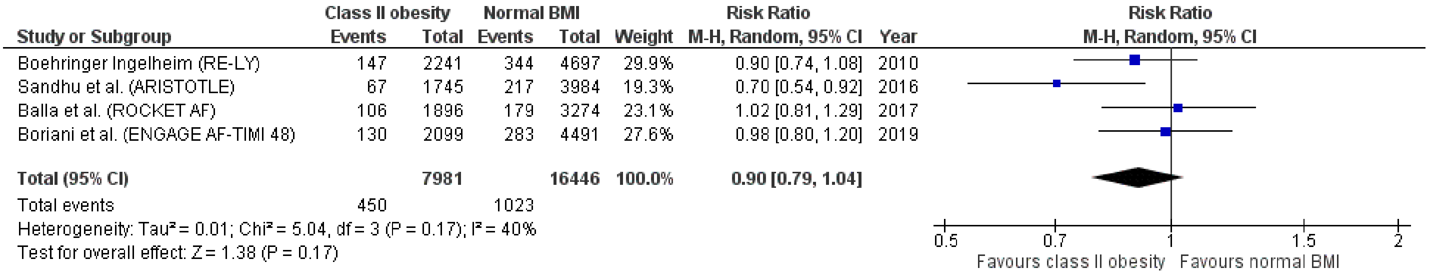

D)

Study or Subgroup	Class II obesity Events	Class II obesity Total	Normal BMI Events	Normal BMI Total	Weight	Risk Ratio M-H, Random, 95% CI	Year
**4.4.1 RCTs**							
Balla et al. (ROCKET AF)	8	1896	23	3274	38.2%	0.60 [0.27, 1.34]	2017
**Subtotal (95% CI)**		**1896**		**3274**	**38.2%**	**0.60 [0.27, 1.34]**	
Total events	8		23				
Heterogeneity: Not applicable							
Test for overall effect: Z = 1.25 (P = 0.21)							
**4.4.2 Observational studies**							
Kaplan et al.	14	1421	30	1720	61.8%	0.56 [0.30, 1.06]	2020
**Subtotal (95% CI)**		**1421**		**1720**	**61.8%**	**0.56 [0.30, 1.06]**	
Total events	14		30				
Heterogeneity: Not applicable							
Test for overall effect: Z = 1.78 (P = 0.08)							
**Total (95% CI)**		**3317**		**4994**	**100.0%**	**0.58 [0.35, 0.95]**	
Total events	22		53				
Heterogeneity: Tau² = 0.00; Chi² = 0.01, df = 1 (P = 0.91); I² = 0%							
Test for overall effect: Z = 2.17 (P = 0.03)							
Test for subgroup differences: Chi² = 0.01, df = 1 (P = 0.91), I² = 0%							

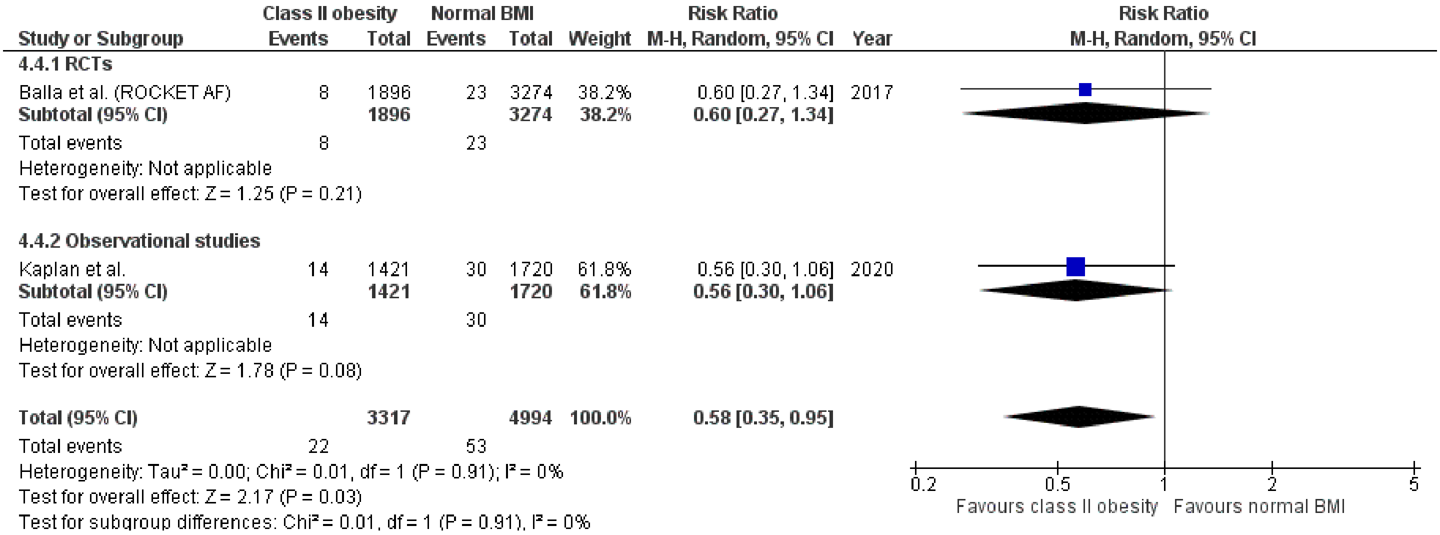

<u>**eFigure 2:**</u> Forest plot of the risk of **A)** stroke or systemic embolism, **B)** all-cause mortality, **C)** major bleeding, and **D)** intracranial bleeding for class II obese (BMI 35-<40 kg/m²) versus normal BMI (18.5-<25 kg/m²) AF patients receiving anticoagulation, categorized according to randomized and observational studies.

AF: atrial fibrillation; ARISTOTLE: the Apixaban for Reduction in Stroke and Other Thromboembolic Events in Atrial Fibrillation trial; BMI: body mass index; CI: confidence interval; ENGAGE AF-TIMI 48: the Effective Anticoagulation with Factor Xa Next Generation in Atrial Fibrillation–Thrombolysis in Myocardial Infarction 48 trial; M-H: Mantel-Haenszel (statistical method); RCT: randomized controlled trial; RE-LY: the Randomized Evaluation of Long-Term Anticoagulation Therapy; ROCKET AF: the Rivaroxaban Once Daily Oral Direct Factor Xa Inhibition Compared with Vitamin K Antagonism for Prevention of Stroke and Embolism Trial in Atrial Fibrillation.

eFigure 3: Impact of morbid obesity on AF-related outcomes

A)

Study or Subgroup	Class III obesity Events	Class III obesity Total	Normal BMI Events	Normal BMI Total	Weight	Risk Ratio M-H, Random, 95% CI	Year
Sandhu et al. (ARISTOTLE)	18	1003	142	4038	40.1%	0.51 [0.31, 0.83]	2016
Boriani et al. (ENGAGE AF-TIMI 48)	26	1149	273	4491	59.9%	0.37 [0.25, 0.55]	2019
**Total (95% CI)**		**2152**		**8529**	**100.0%**	**0.42 [0.31, 0.57]**	
Total events	44		415				

Heterogeneity: Tau² = 0.00; Chi² = 0.98, df = 1 (P = 0.32); I² = 0%
Test for overall effect: Z = 5.50 (P < 0.00001)

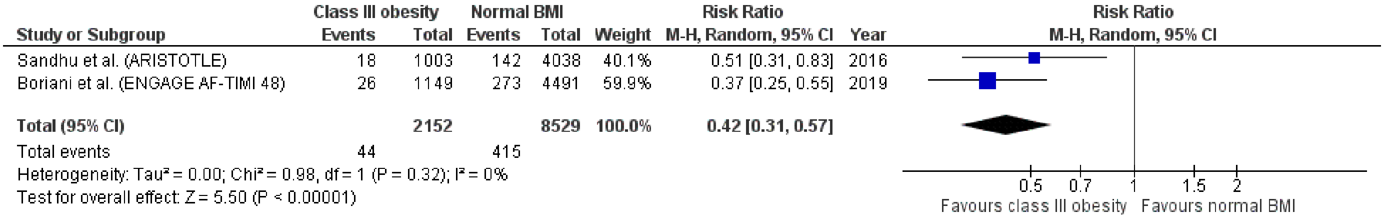

B)

Study or Subgroup	Class III obesity Events	Class III obesity Total	Normal BMI Events	Normal BMI Total	Weight	Risk Ratio M-H, Random, 95% CI	Year
Sandhu et al. (ARISTOTLE)	55	1003	397	4038	37.4%	0.56 [0.42, 0.73]	2016
Boriani et al. (ENGAGE AF-TIMI 48)	90	1149	629	4491	62.6%	0.56 [0.45, 0.69]	2019
**Total (95% CI)**		**2152**		**8529**	**100.0%**	**0.56 [0.47, 0.66]**	
Total events	145		1026				

Heterogeneity: Tau² = 0.00; Chi² = 0.00, df = 1 (P = 0.99); I² = 0%
Test for overall effect: Z = 6.83 (P < 0.00001)

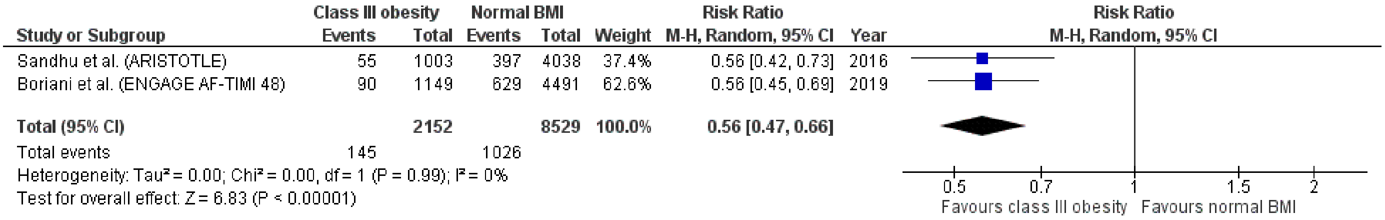

C)

Study or Subgroup	Class III obesity Events	Class III obesity Total	Normal BMI Events	Normal BMI Total	Weight	Risk Ratio M-H, Random, 95% CI	Year
Sandhu et al. (ARISTOTLE)	28	997	217	3984	47.5%	0.52 [0.35, 0.76]	2016
Boriani et al. (ENGAGE AF-TIMI 48)	73	1149	283	4491	52.5%	1.01 [0.79, 1.29]	2019
**Total (95% CI)**		**2146**		**8475**	**100.0%**	**0.73 [0.38, 1.42]**	
Total events	101		500				

Heterogeneity: Tau² = 0.20; Chi² = 8.28, df = 1 (P = 0.004); I² = 88%
Test for overall effect: Z = 0.92 (P = 0.36)

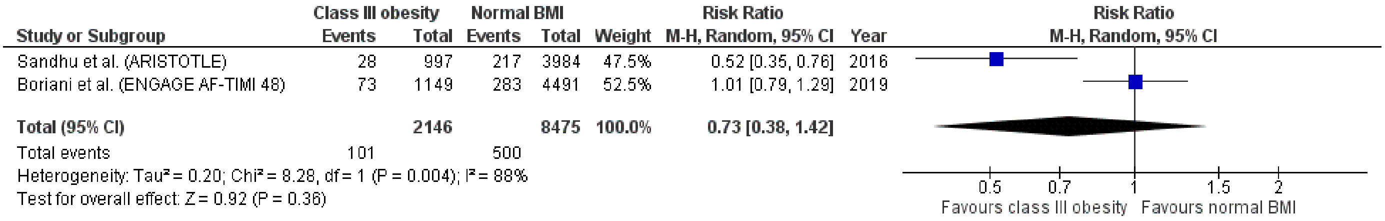

eFigure 3: Forest plot of the risk of **A)** stroke or systemic embolism, **B)** all-cause mortality and **C)** major bleeding for anticoagulated morbidly obese (BMI ≥40 kg/m²) versus normal BMI (18.5-<25 kg/m²) AF patients receiving anticoagulation, based on results from randomized studies (no data regarding intracranial bleeding risk).

AF: atrial fibrillation; ARISTOTLE: the Apixaban for Reduction in Stroke and Other Thromboembolic Events in Atrial Fibrillation trial; BMI: body mass index; CI: confidence interval; ENGAGE AF-TIMI 48: the Effective Anticoagulation with Factor Xa Next Generation in Atrial Fibrillation–Thrombolysis in Myocardial Infarction 48 trial; M-H: Mantel-Haenszel (statistical method); RCT: randomized controlled trial.

eFigure 4: Sensitivity analysis including non-anticoagulated AF patients

eFigure 4.1: Impact of underweight on AF-related outcomes (sensitivity analysis)

A)

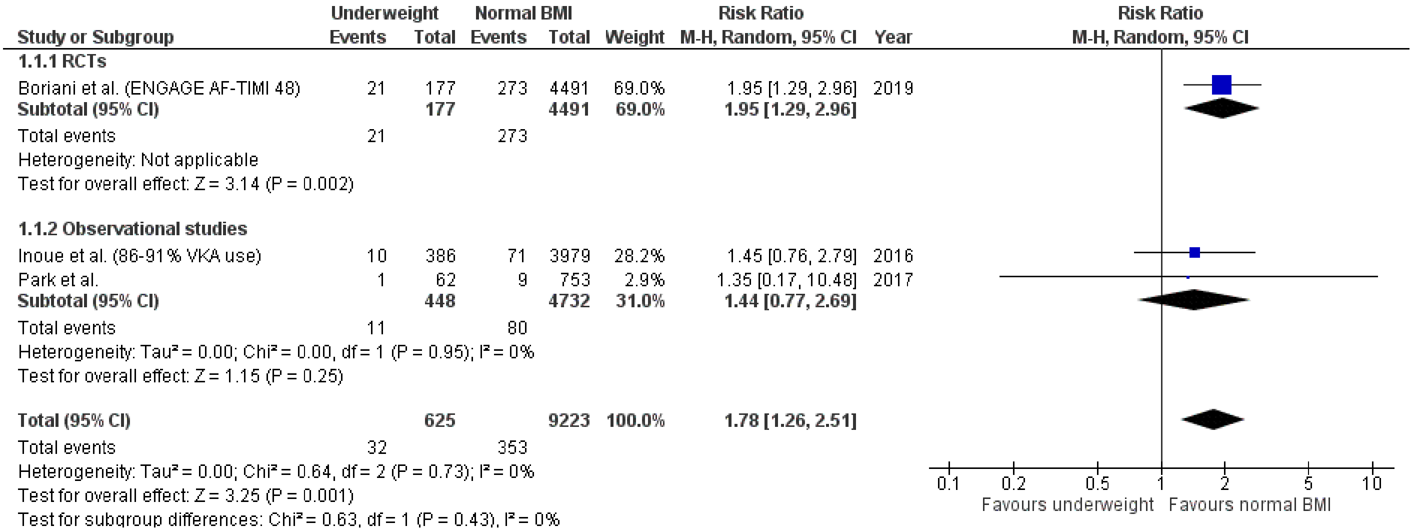

B)

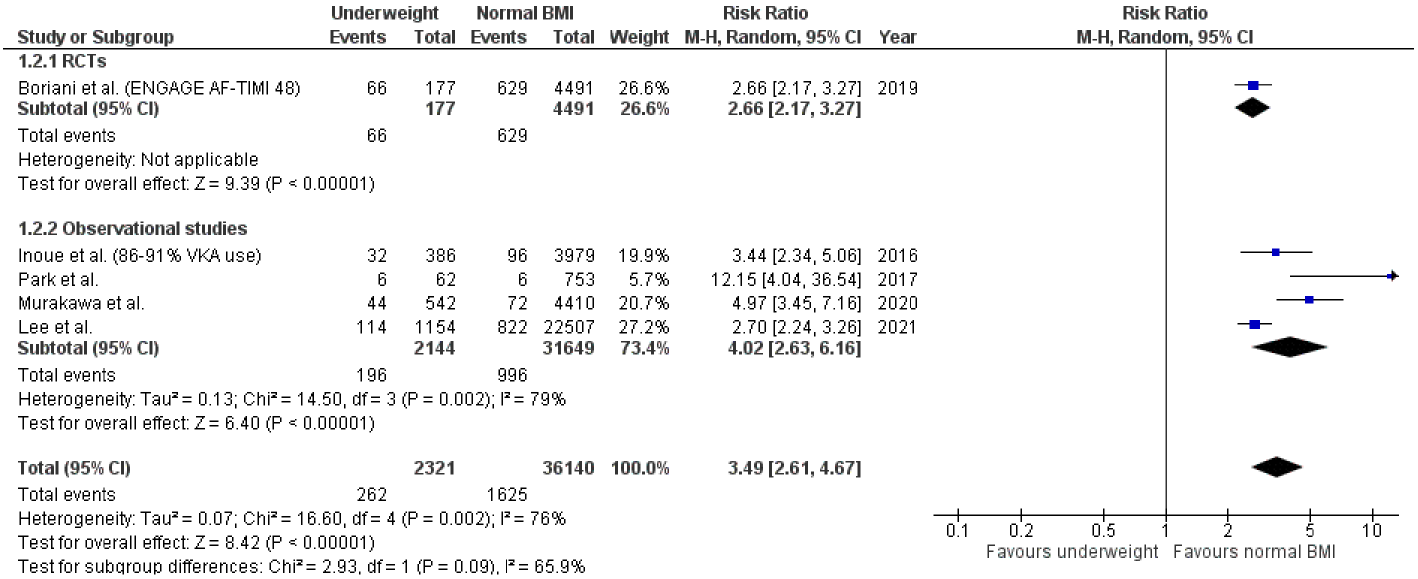

C)

Study or Subgroup	Underweight Events	Underweight Total	Normal BMI Events	Normal BMI Total	Weight	Risk Ratio M-H, Random, 95% CI	Year
**1.3.1 RCTs**							
Boriani et al. (ENGAGE AF-TIMI 48)	17	177	283	4491	23.1%	1.52 [0.96, 2.43]	2019
**Subtotal (95% CI)**		**177**		**4491**	**23.1%**	**1.52 [0.96, 2.43]**	
Total events	17		283				
Heterogeneity: Not applicable							
Test for overall effect: Z = 1.77 (P = 0.08)							
**1.3.2 Observational studies**							
Inoue et al. (86-91% VKA use)	14	386	79	3979	21.7%	1.83 [1.04, 3.19]	2016
Park et al.	6	62	16	753	16.2%	4.55 [1.85, 11.22]	2017
Murakawa et al.	9	542	70	4410	19.6%	1.05 [0.53, 2.08]	2020
Lee et al.	8	1154	283	22507	19.4%	0.55 [0.27, 1.11]	2021
**Subtotal (95% CI)**		**2144**		**31649**	**76.9%**	**1.44 [0.64, 3.22]**	
Total events	37		448				
Heterogeneity: Tau² = 0.54; Chi² = 15.95, df = 3 (P = 0.001); I² = 81%							
Test for overall effect: Z = 0.89 (P = 0.38)							
**Total (95% CI)**		**2321**		**36140**	**100.0%**	**1.44 [0.82, 2.56]**	
Total events	54		731				
Heterogeneity: Tau² = 0.31; Chi² = 15.93, df = 4 (P = 0.003); I² = 75%							
Test for overall effect: Z = 1.26 (P = 0.21)							
Test for subgroup differences: Chi² = 0.01, df = 1 (P = 0.90), I² = 0%							

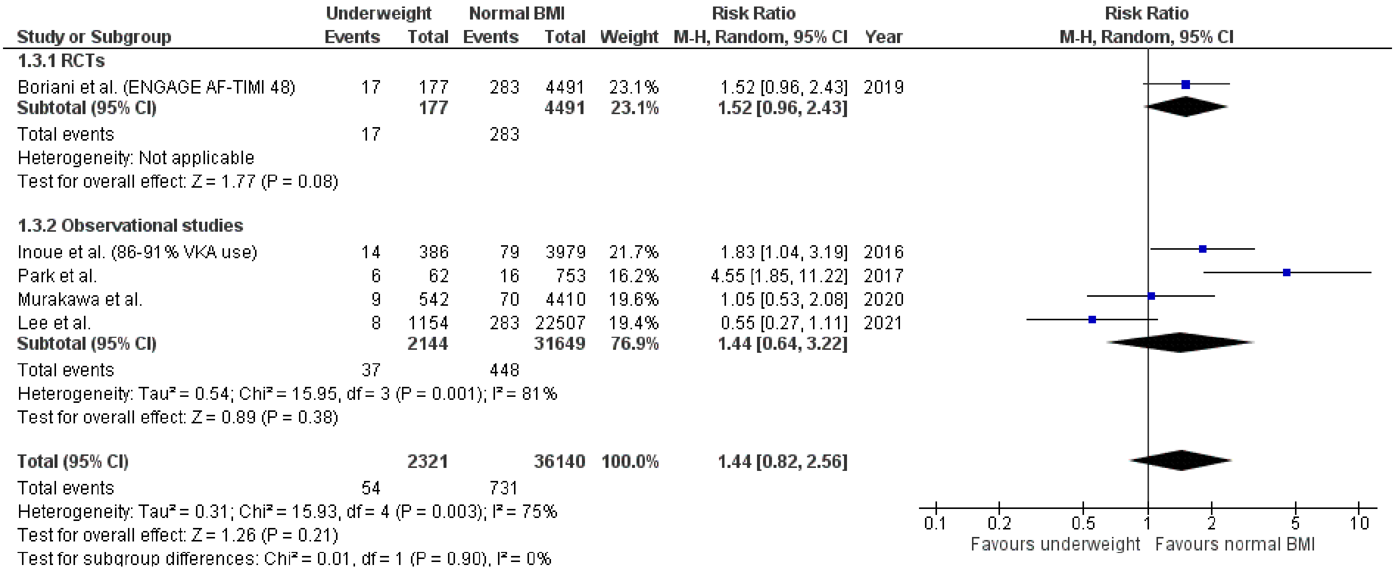

D)

Study or Subgroup	Underweight Events	Underweight Total	Normal BMI Events	Normal BMI Total	Weight	Risk Ratio M-H, Random, 95% CI	Year
Lee et al.	2	1154	86	22507	100.0%	0.45 [0.11, 1.84]	2021
**Total (95% CI)**		**1154**		**22507**	**100.0%**	**0.45 [0.11, 1.84]**	
Total events	2		86				
Heterogeneity: Not applicable							
Test for overall effect: Z = 1.11 (P = 0.27)							

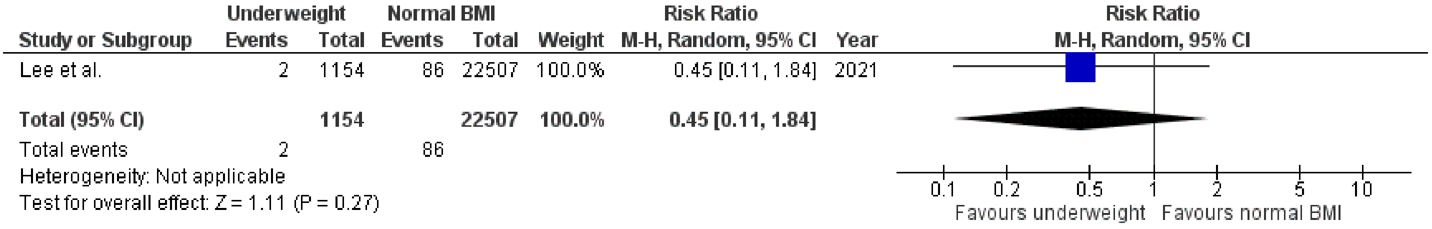

<u>eFigure 4.1:</u> Forest plot of the risk of **A)** stroke or systemic embolism, **B)** all-cause mortality, **C)** major bleeding, and **D)** intracranial bleeding for underweight (BMI <18.5 kg/m²) versus normal BMI (18.5-<25 kg/m²) AF patients with and without anticoagulation, categorized according to randomized and observational studies.

AF: atrial fibrillation; BMI: body mass index; CI: confidence interval; ENGAGE AF-TIMI 48: the Effective Anticoagulation with Factor Xa Next Generation in Atrial Fibrillation–Thrombolysis in Myocardial Infarction 48 trial; M-H: Mantel-Haenszel (statistical method); RCT: randomized controlled trial.

eFigure 4.2: Impact of overweight on AF-related outcomes (sensitivity analysis)

A)

Study or Subgroup	Overweight Events	Overweight Total	Normal BMI Events	Normal BMI Total	Weight	Risk Ratio M-H, Random, 95% CI	Year
**2.1.1 RCTs**							
Boehringer Ingelheim (RE-LY)	196	7111	181	4697	16.6%	0.72 [0.59, 0.87]	2010
Sandhu et al. (ARISTOTLE)	174	6671	142	4038	14.6%	0.74 [0.60, 0.92]	2016
Balla et al. (ROCKET AF)	226	5535	166	3289	16.9%	0.81 [0.67, 0.98]	2017
Boriani et al. (ENGAGE AF-TIMI 48)	407	7903	273	4491	23.6%	0.85 [0.73, 0.98]	2019
**Subtotal (95% CI)**		**27220**		**16515**	**71.7%**	**0.79 [0.72, 0.87]**	
Total events	1003		762				
Heterogeneity: Tau² = 0.00; Chi² = 2.19, df = 3 (P = 0.53); I² = 0%							
Test for overall effect: Z = 5.02 (P < 0.00001)							
**2.1.2 Observational studies**							
Overvad et al. (18-24% VKA use)	94	1414	55	954	7.9%	1.15 [0.84, 1.59]	2013
Inoue et al. (86-91% VKA use)	27	1739	71	3979	4.5%	0.87 [0.56, 1.35]	2016
Pandey et al. (69-75% VKA use)	112	3164	100	2076	10.9%	0.73 [0.56, 0.96]	2016
Park et al.	1	538	9	753	0.2%	0.16 [0.02, 1.22]	2017
Kaplan et al.	19	2804	10	1720	1.6%	1.17 [0.54, 2.50]	2020
Bertomeu-Gonzalez et al.	41	871	18	358	3.1%	0.94 [0.55, 1.61]	2020
**Subtotal (95% CI)**		**10530**		**9840**	**28.3%**	**0.90 [0.71, 1.14]**	
Total events	294		263				
Heterogeneity: Tau² = 0.03; Chi² = 7.78, df = 5 (P = 0.17); I² = 36%							
Test for overall effect: Z = 0.87 (P = 0.38)							
**Total (95% CI)**		**37750**		**26355**	**100.0%**	**0.81 [0.74, 0.90]**	
Total events	1297		1025				
Heterogeneity: Tau² = 0.00; Chi² = 11.32, df = 9 (P = 0.25); I² = 21%							
Test for overall effect: Z = 4.13 (P < 0.0001)							
Test for subgroup differences: Chi² = 0.99, df = 1 (P = 0.32), I² = 0%							

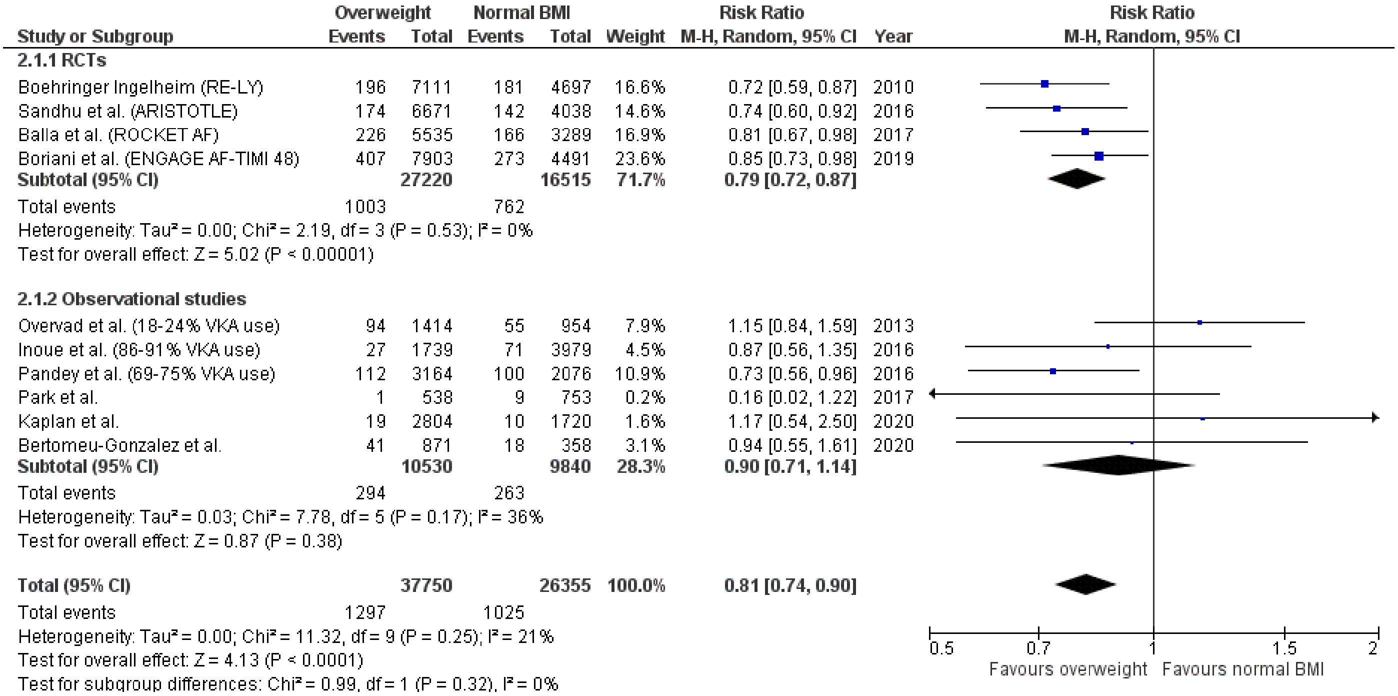

B)

Study or Subgroup	Overweight Events	Overweight Total	Normal BMI Events	Normal BMI Total	Weight	Risk Ratio M-H, Random, 95% CI	Year
**2.2.1 RCTs**							
Sandhu et al. (ARISTOTLE)	429	6671	397	4038	13.5%	0.65 [0.57, 0.75]	2016
Boriani et al. (ENGAGE AF-TIMI 48)	858	7903	629	4491	14.2%	0.78 [0.70, 0.85]	2019
**Subtotal (95% CI)**		**14574**		**8529**	**27.7%**	**0.72 [0.61, 0.85]**	
Total events	1287		1026				
Heterogeneity: Tau² = 0.01; Chi² = 4.21, df = 1 (P = 0.04); I² = 76%							
Test for overall effect: Z = 3.94 (P < 0.0001)							
**2.2.2 Observational studies**							
Badheka et al. (87% VKA use)	108	965	103	637	10.5%	0.69 [0.54, 0.89]	2010
Overvad et al. (18-24% VKA use)	233	1414	121	954	11.7%	1.30 [1.06, 1.59]	2013
Pandey et al. (69-75% VKA use)	340	3164	339	2076	13.3%	0.66 [0.57, 0.76]	2016
Inoue et al. (86-91% VKA use)	22	1739	96	3979	6.0%	0.52 [0.33, 0.83]	2016
Park et al.	3	538	6	753	1.0%	0.70 [0.18, 2.79]	2017
Murakawa et al.	33	2167	72	4410	6.9%	0.93 [0.62, 1.40]	2020
Bertomeu-Gonzalez et al.	119	871	52	358	9.2%	0.94 [0.70, 1.27]	2020
Lee et al.	389	16779	822	22507	13.7%	0.63 [0.56, 0.71]	2021
**Subtotal (95% CI)**		**27637**		**35674**	**72.3%**	**0.78 [0.63, 0.97]**	
Total events	1247		1611				
Heterogeneity: Tau² = 0.07; Chi² = 44.48, df = 7 (P < 0.00001); I² = 84%							
Test for overall effect: Z = 2.20 (P = 0.03)							
**Total (95% CI)**		**42211**		**44203**	**100.0%**	**0.76 [0.66, 0.88]**	
Total events	2534		2637				
Heterogeneity: Tau² = 0.04; Chi² = 48.67, df = 9 (P < 0.00001); I² = 82%							
Test for overall effect: Z = 3.67 (P = 0.0002)							
Test for subgroup differences: Chi² = 0.37, df = 1 (P = 0.54), I² = 0%							

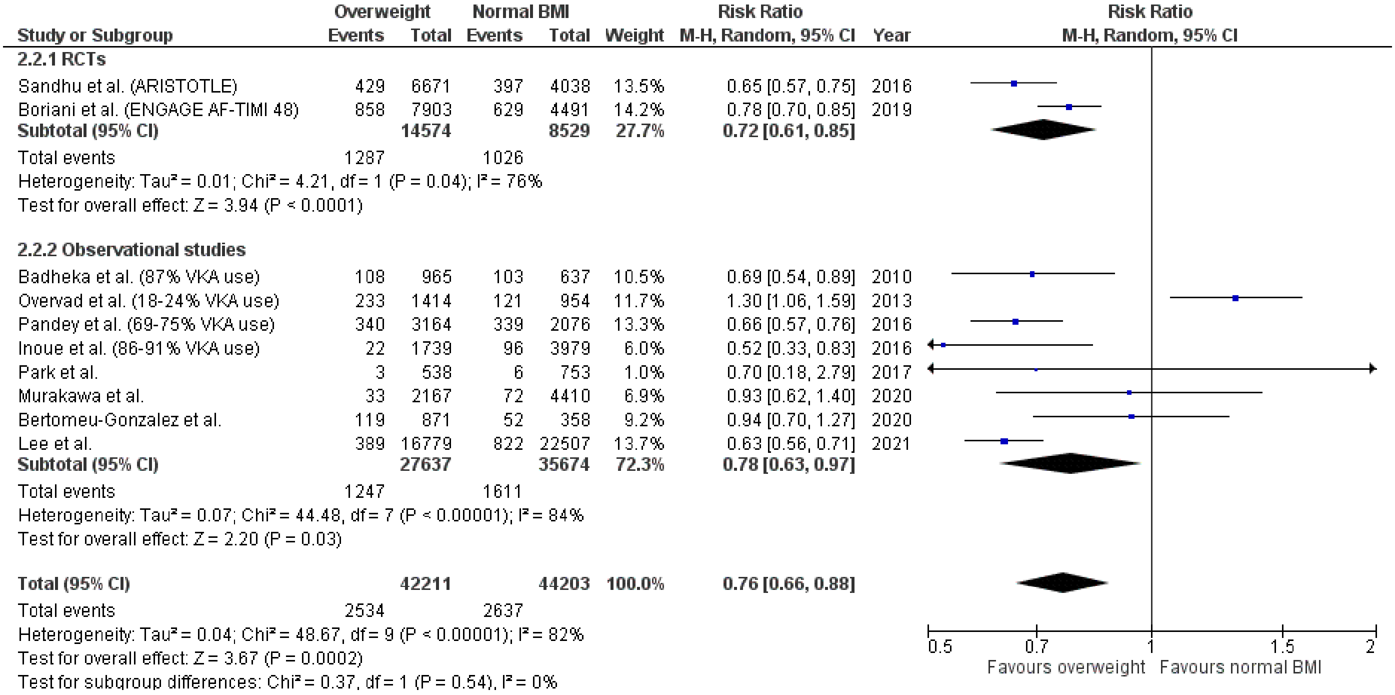

C)

Study or Subgroup	Overweight Events	Overweight Total	Normal BMI Events	Normal BMI Total	Weight	Risk Ratio M-H, Random, 95% CI	Year
**2.3.1 RCTs**							
Boehringer Ingelheim (RE-LY)	424	7111	344	4697	15.8%	0.81 [0.71, 0.93]	2010
Sandhu et al. (ARISTOTLE)	270	6618	217	3984	13.8%	0.75 [0.63, 0.89]	2016
Balla et al. (ROCKET AF)	314	5529	179	3274	13.6%	1.04 [0.87, 1.24]	2017
Boriani et al. (ENGAGE AF-TIMI 48)	472	7903	283	4491	15.5%	0.95 [0.82, 1.09]	2019
**Subtotal (95% CI)**		**27161**		**16446**	**58.6%**	**0.88 [0.77, 1.01]**	
Total events	1480		1023				
Heterogeneity: Tau² = 0.01; Chi² = 8.87, df = 3 (P = 0.03); I² = 66%							
Test for overall effect: Z = 1.87 (P = 0.06)							
**2.3.2 Observational studies**							
Pandey et al. (69-75% VKA use)	209	3164	143	2076	12.2%	0.96 [0.78, 1.18]	2016
Inoue et al. (86-91% VKA use)	26	1739	79	3979	5.0%	0.75 [0.49, 1.17]	2016
Park et al.	7	538	16	753	1.5%	0.61 [0.25, 1.48]	2017
Murakawa et al.	32	2167	70	4410	5.4%	0.93 [0.61, 1.41]	2020
Bertomeu-Gonzalez et al.	75	871	23	358	4.8%	1.34 [0.85, 2.10]	2020
Lee et al.	141	16779	283	22507	12.4%	0.67 [0.55, 0.82]	2021
**Subtotal (95% CI)**		**25258**		**34083**	**41.4%**	**0.86 [0.69, 1.07]**	
Total events	490		614				
Heterogeneity: Tau² = 0.04; Chi² = 11.65, df = 5 (P = 0.04); I² = 57%							
Test for overall effect: Z = 1.36 (P = 0.17)							
**Total (95% CI)**		**52419**		**50529**	**100.0%**	**0.87 [0.78, 0.97]**	
Total events	1970		1637				
Heterogeneity: Tau² = 0.02; Chi² = 21.00, df = 9 (P = 0.01); I² = 57%							
Test for overall effect: Z = 2.47 (P = 0.01)							
Test for subgroup differences: Chi² = 0.03, df = 1 (P = 0.86), I² = 0%							

Risk Ratio M-H, Random, 95% CI

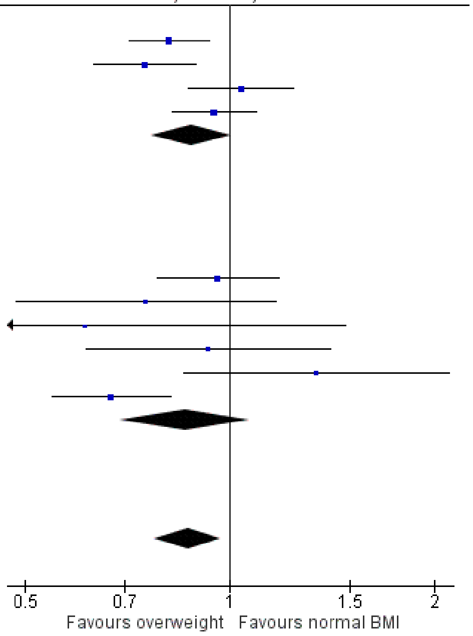

D)

Study or Subgroup	Overweight Events	Overweight Total	Normal BMI Events	Normal BMI Total	Weight	Risk Ratio M-H, Random, 95% CI	Year
**2.4.1 RCTs**							
Balla et al. (ROCKET AF)	34	5529	23	3274	22.8%	0.88 [0.52, 1.48]	2017
**Subtotal (95% CI)**		**5529**		**3274**	**22.8%**	**0.88 [0.52, 1.48]**	
Total events	34		23				
Heterogeneity: Not applicable							
Test for overall effect: Z = 0.49 (P = 0.62)							
**2.4.2 Observational studies**							
Kaplan et al.	31	2804	30	1720	25.5%	0.63 [0.39, 1.04]	2020
Lee et al.	49	16779	86	22507	51.7%	0.76 [0.54, 1.08]	2021
**Subtotal (95% CI)**		**19583**		**24227**	**77.2%**	**0.72 [0.54, 0.96]**	
Total events	80		116				
Heterogeneity: Tau² = 0.00; Chi² = 0.36, df = 1 (P = 0.55); I² = 0%							
Test for overall effect: Z = 2.26 (P = 0.02)							
**Total (95% CI)**		**25112**		**27501**	**100.0%**	**0.75 [0.58, 0.97]**	
Total events	114		139				
Heterogeneity: Tau² = 0.00; Chi² = 0.78, df = 2 (P = 0.68); I² = 0%							
Test for overall effect: Z = 2.22 (P = 0.03)							
Test for subgroup differences: Chi² = 0.42, df = 1 (P = 0.52), I² = 0%							

Risk Ratio M-H, Random, 95% CI

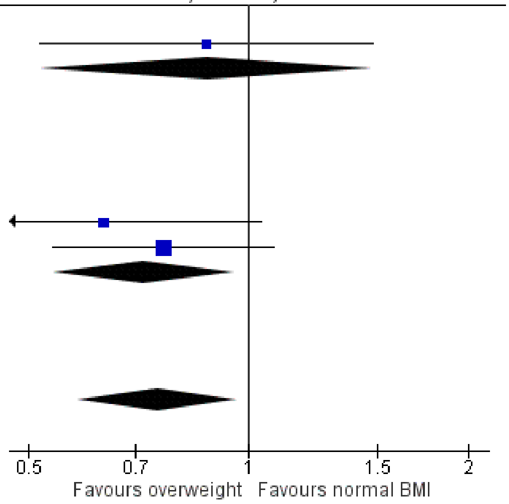

eFigure 4.2: Forest plot of the risk of **A)** stroke or systemic embolism, **B)** all-cause mortality, **C)** major bleeding, and **D)** intracranial bleeding for overweight (BMI 25-<30 kg/m²) versus normal BMI (18.5-<25 kg/m²) AF patients with and without anticoagulation, categorized according to randomized and observational studies.

AF: atrial fibrillation; ARISTOTLE: the Apixaban for Reduction in Stroke and Other Thromboembolic Events in Atrial Fibrillation trial; BMI: body mass index; CI: confidence interval; ENGAGE AF-TIMI 48: the Effective Anticoagulation with Factor Xa Next Generation in Atrial Fibrillation–Thrombolysis in Myocardial Infarction 48 trial; M-H: Mantel-Haenszel (statistical method); RCT: randomized controlled trial; RE-LY: the Randomized Evaluation of Long-Term Anticoagulation Therapy; ROCKET AF: the Rivaroxaban Once Daily Oral Direct Factor Xa Inhibition Compared with Vitamin K Antagonism for Prevention of Stroke and Embolism Trial in Atrial Fibrillation.

eFigure 4.3: Impact of obesity on AF-related outcomes (sensitivity analysis)

A)

Study or Subgroup	Obesity Events	Obesity Total	Normal BMI Events	Normal BMI Total	Weight	Risk Ratio M-H, Random, 95% CI	Year
**3.1.1 RCTs**							
Boehringer Ingelheim (RE-LY)	144	6279	181	4697	16.1%	0.60 [0.48, 0.74]	2010
Sandhu et al. (ARISTOTLE)	150	7131	142	4038	15.5%	0.60 [0.48, 0.75]	2016
Balla et al. (ROCKET AF)	179	5206	166	3289	16.6%	0.68 [0.55, 0.84]	2017
Boriani et al. (ENGAGE AF-TIMI 48)	316	8457	273	4491	19.3%	0.61 [0.52, 0.72]	2019
**Subtotal (95% CI)**		**27073**		**16515**	**67.5%**	**0.62 [0.56, 0.69]**	
Total events	789		762				
Heterogeneity: Tau² = 0.00; Chi² = 1.04, df = 3 (P = 0.79); I² = 0%							
Test for overall effect: Z = 9.53 (P < 0.00001)							
**3.1.2 Observational studies**							
Overvad et al. (18-24% VKA use)	51	767	55	954	9.5%	1.15 [0.80, 1.67]	2013
Pandey et al. (69-75% VKA use)	103	4273	100	2076	13.3%	0.50 [0.38, 0.66]	2016
Inoue et al. (86-91% VKA use)	3	275	71	3979	1.5%	0.61 [0.19, 1.93]	2016
Bertomeu-Gonzalez et al.	34	727	18	358	5.3%	0.93 [0.53, 1.62]	2020
Kaplan et al.	16	3118	10	1720	2.9%	0.88 [0.40, 1.94]	2020
**Subtotal (95% CI)**		**9160**		**9087**	**32.5%**	**0.79 [0.51, 1.22]**	
Total events	207		254				
Heterogeneity: Tau² = 0.16; Chi² = 14.35, df = 4 (P = 0.006); I² = 72%							
Test for overall effect: Z = 1.08 (P = 0.28)							
**Total (95% CI)**		**36233**		**25602**	**100.0%**	**0.66 [0.57, 0.76]**	
Total events	996		1016				
Heterogeneity: Tau² = 0.02; Chi² = 16.73, df = 8 (P = 0.03); I² = 52%							
Test for overall effect: Z = 5.65 (P < 0.00001)							
Test for subgroup differences: Chi² = 1.05, df = 1 (P = 0.30), I² = 5.2%							

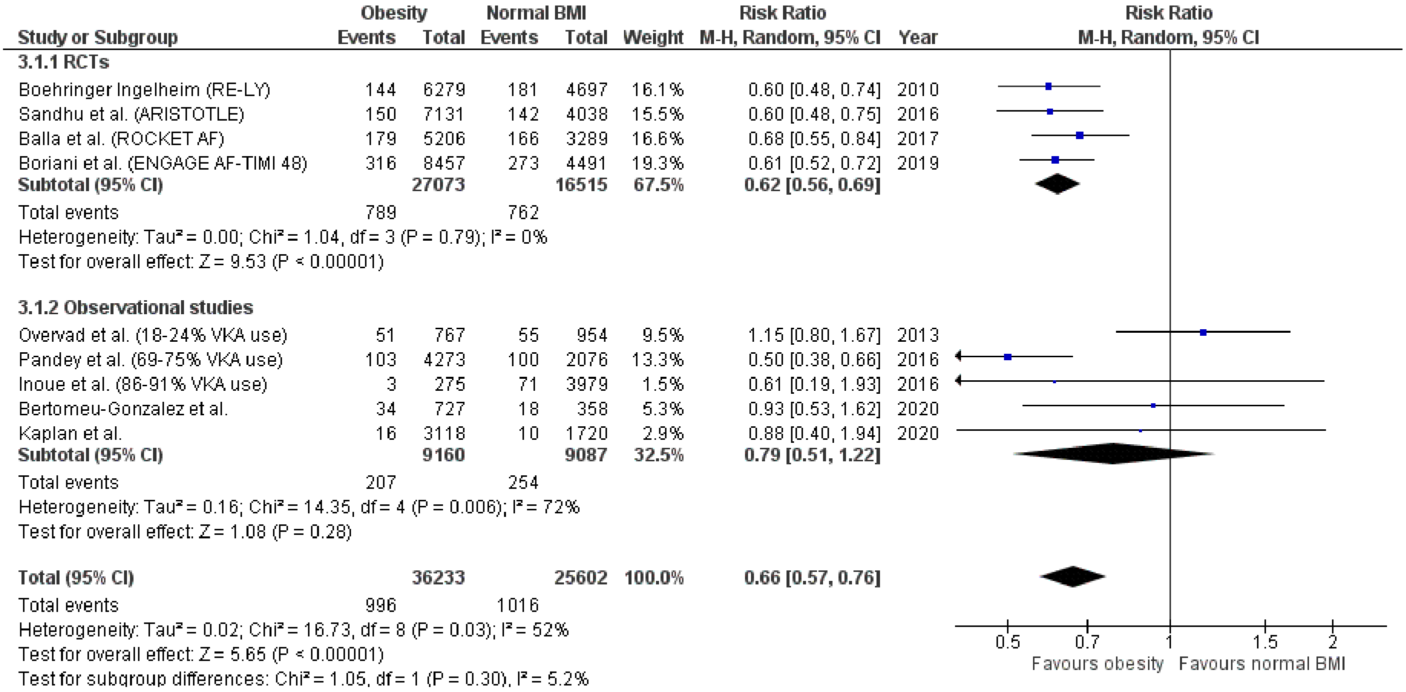

B)

Study or Subgroup	Obesity Events	Obesity Total	Normal BMI Events	Normal BMI Total	Weight	Risk Ratio M-H, Random, 95% CI	Year
**3.2.1 RCTs**							
Sandhu et al. (ARISTOTLE)	398	7131	397	4038	13.6%	0.57 [0.50, 0.65]	2016
Boriani et al. (ENGAGE AF-TIMI 48)	793	8457	629	4491	13.8%	0.67 [0.61, 0.74]	2019
**Subtotal (95% CI)**		**15588**		**8529**	**27.4%**	**0.62 [0.53, 0.73]**	
Total events	1191		1026				
Heterogeneity: Tau² = 0.01; Chi² = 3.81, df = 1 (P = 0.05); I² = 74%							
Test for overall effect: Z = 5.80 (P < 0.00001)							
**3.2.2 Observational studies**							
Badheka et al. (87% VKA use)	93	890	103	637	12.2%	0.65 [0.50, 0.84]	2010
Overvad et al. (18-24% VKA use)	156	767	121	954	12.7%	1.60 [1.29, 1.99]	2013
Pandey et al. (69-75% VKA use)	346	4273	339	2076	13.5%	0.50 [0.43, 0.57]	2016
Inoue et al. (86-91% VKA use)	9	275	96	3979	6.9%	1.36 [0.69, 2.66]	2016
Murakawa et al.	4	499	72	4410	4.2%	0.49 [0.18, 1.34]	2020
Bertomeu-Gonzalez et al.	84	727	52	358	11.4%	0.80 [0.58, 1.10]	2020
Lee et al.	47	2733	822	22507	11.8%	0.47 [0.35, 0.63]	2021
**Subtotal (95% CI)**		**10164**		**34921**	**72.6%**	**0.75 [0.49, 1.15]**	
Total events	739		1605				
Heterogeneity: Tau² = 0.28; Chi² = 90.93, df = 6 (P < 0.00001); I² = 93%							
Test for overall effect: Z = 1.33 (P = 0.18)							
**Total (95% CI)**		**25752**		**43450**	**100.0%**	**0.71 [0.55, 0.91]**	
Total events	1930		2631				
Heterogeneity: Tau² = 0.11; Chi² = 95.94, df = 8 (P < 0.00001); I² = 92%							
Test for overall effect: Z = 2.75 (P = 0.006)							
Test for subgroup differences: Chi² = 0.68, df = 1 (P = 0.41), I² = 0%							

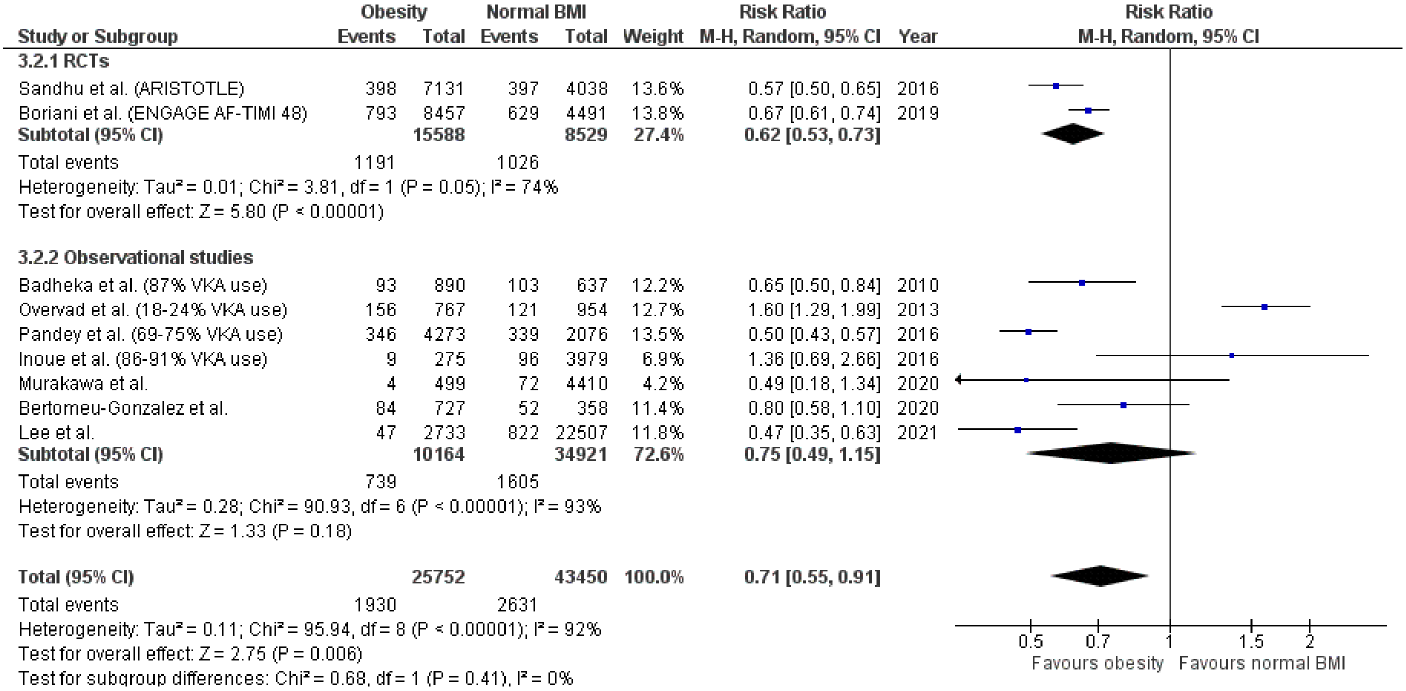

C)

Study or Subgroup	Obesity Events	Obesity Total	Normal BMI Events	Normal BMI Total	Weight	Risk Ratio M-H, Random, 95% CI	Year
**3.3.1 RCTs**							
Boehringer Ingelheim (RE-LY)	394	6279	344	4697	22.6%	0.86 [0.75, 0.99]	2010
Sandhu et al. (ARISTOTLE)	281	7074	217	3984	16.9%	0.73 [0.61, 0.87]	2016
Balla et al. (ROCKET AF)	279	5199	179	3274	15.6%	0.98 [0.82, 1.18]	2017
Boriani et al. (ENGAGE AF-TIMI 48)	521	8457	283	4491	22.5%	0.98 [0.85, 1.12]	2019
**Subtotal (95% CI)**		**27009**		**16446**	**77.6%**	**0.88 [0.77, 1.00]**	
Total events	1475		1023				
Heterogeneity: Tau² = 0.01; Chi² = 8.20, df = 3 (P = 0.04); I² = 63%							
Test for overall effect: Z = 1.90 (P = 0.06)							
**3.3.2 Observational studies**							
Pandey et al. (69-75% VKA use)	254	4273	143	2076	13.8%	0.86 [0.71, 1.05]	2016
Inoue et al. (86-91% VKA use)	5	275	79	3979	0.9%	0.92 [0.37, 2.24]	2016
Murakawa et al.	7	499	70	4410	1.1%	0.88 [0.41, 1.91]	2020
Bertomeu-Gonzalez et al.	48	727	23	358	2.9%	1.03 [0.64, 1.66]	2020
Lee et al.	24	2733	283	22507	3.8%	0.70 [0.46, 1.06]	2021
**Subtotal (95% CI)**		**8507**		**33330**	**22.4%**	**0.85 [0.73, 1.00]**	
Total events	338		598				
Heterogeneity: Tau² = 0.00; Chi² = 1.52, df = 4 (P = 0.82); I² = 0%							
Test for overall effect: Z = 1.91 (P = 0.06)							
**Total (95% CI)**		**35516**		**49776**	**100.0%**	**0.88 [0.81, 0.95]**	
Total events	1813		1621				
Heterogeneity: Tau² = 0.00; Chi² = 9.86, df = 8 (P = 0.28); I² = 19%							
Test for overall effect: Z = 3.09 (P = 0.002)							
Test for subgroup differences: Chi² = 0.08, df = 1 (P = 0.77), I² = 0%							

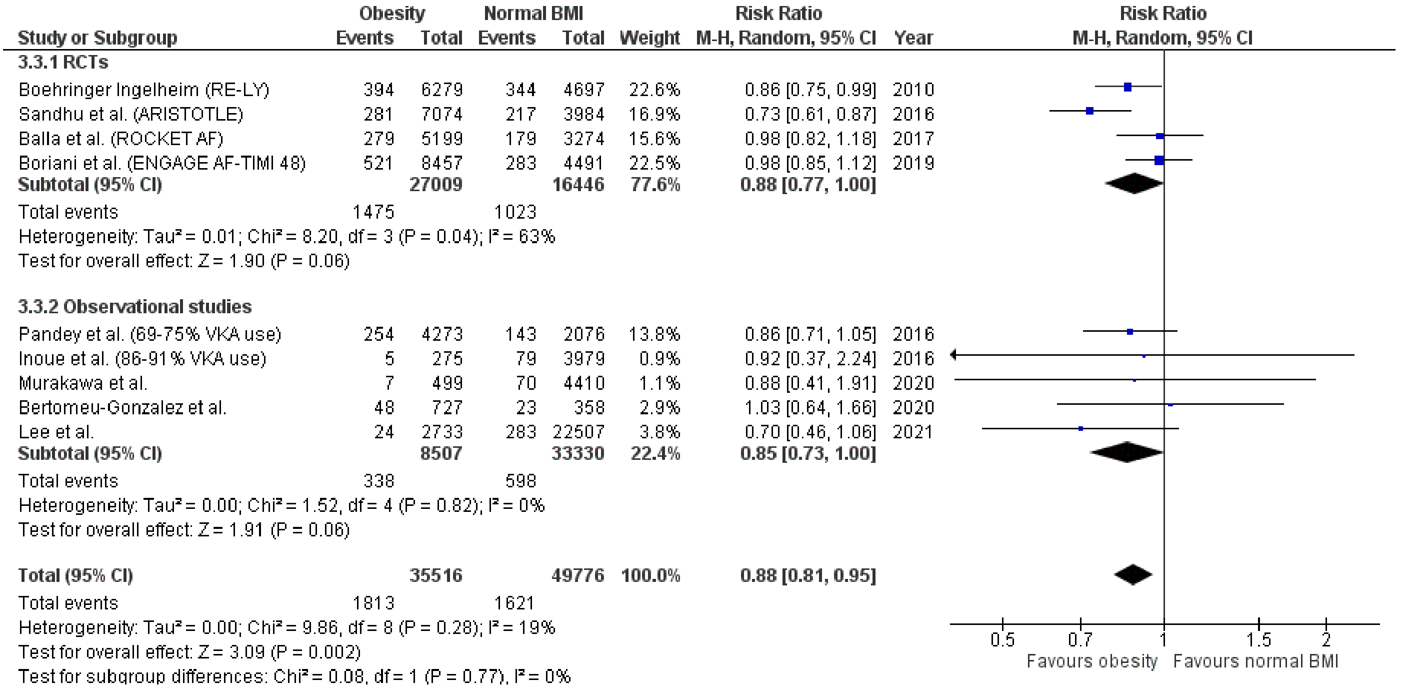

D)

Study or Subgroup	Obesity Events	Obesity Total	Normal BMI Events	Normal BMI Total	Weight	Risk Ratio M-H, Random, 95% CI	Year
**3.4.1 RCTs**							
Balla et al. (ROCKET AF)	22	5199	23	3274	35.9%	0.60 [0.34, 1.08]	2017
**Subtotal (95% CI)**		**5199**		**3274**	**35.9%**	**0.60 [0.34, 1.08]**	
Total events	22		23				
Heterogeneity: Not applicable							
Test for overall effect: Z = 1.70 (P = 0.09)							
**3.4.2 Observational studies**							
Kaplan et al.	31	3118	30	1720	49.1%	0.57 [0.35, 0.94]	2020
Lee et al.	5	2733	86	22507	15.0%	0.48 [0.19, 1.18]	2021
**Subtotal (95% CI)**		**5851**		**24227**	**64.1%**	**0.55 [0.35, 0.85]**	
Total events	36		116				
Heterogeneity: Tau² = 0.00; Chi² = 0.11, df = 1 (P = 0.74); I² = 0%							
Test for overall effect: Z = 2.71 (P = 0.007)							
**Total (95% CI)**		**11050**		**27501**	**100.0%**	**0.57 [0.40, 0.80]**	
Total events	58		139				
Heterogeneity: Tau² = 0.00; Chi² = 0.18, df = 2 (P = 0.91); I² = 0%							
Test for overall effect: Z = 3.19 (P = 0.001)							
Test for subgroup differences: Chi² = 0.07, df = 1 (P = 0.80), I² = 0%							

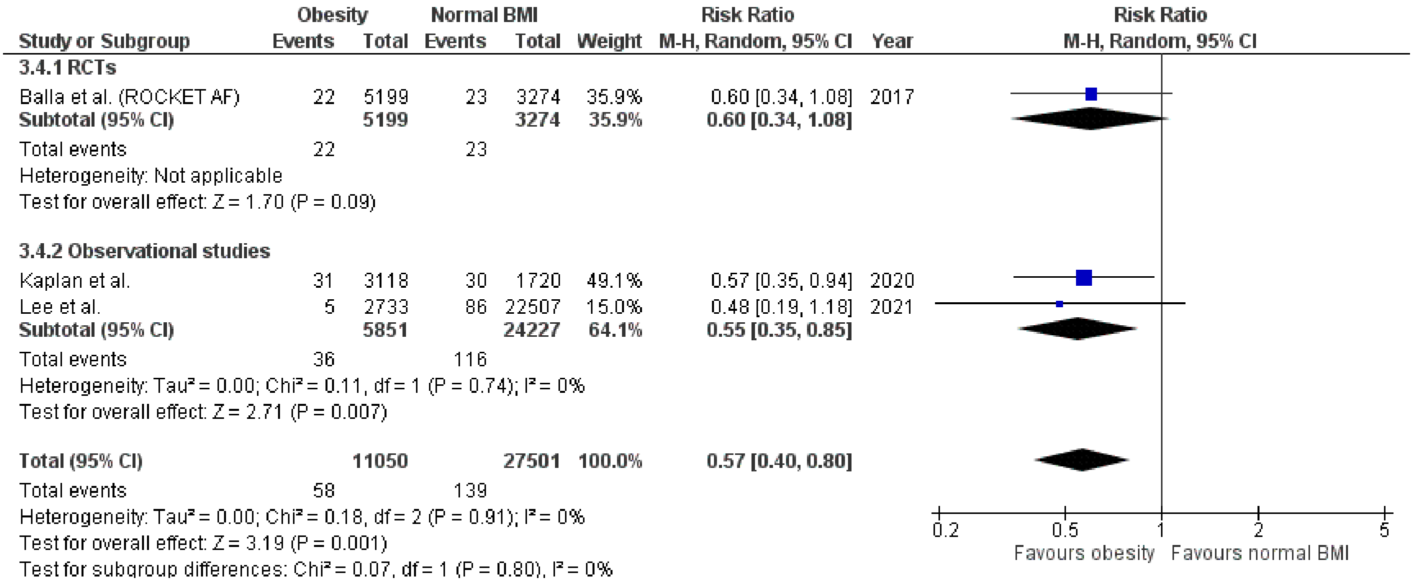

eFigure 4.3: Forest plot of the risk of **A)** stroke or systemic embolism, **B)** all-cause mortality, **C)** major bleeding, and **D)** intracranial bleeding for obese (BMI ≥30 kg/m²) versus normal BMI (18.5-<25 kg/m²) AF patients with and without anticoagulation, categorized according to randomized and observational studies.

AF: atrial fibrillation; ARISTOTLE: the Apixaban for Reduction in Stroke and Other Thromboembolic Events in Atrial Fibrillation trial; BMI: body mass index; CI: confidence interval; ENGAGE AF-TIMI 48: the Effective Anticoagulation with Factor Xa Next Generation in Atrial Fibrillation–Thrombolysis in Myocardial Infarction 48 trial; M-H: Mantel-Haenszel (statistical method); RCT: randomized controlled trial; RE-LY: the Randomized Evaluation of Long-Term Anticoagulation Therapy; ROCKET AF: the Rivaroxaban Once Daily Oral Direct Factor Xa Inhibition Compared with Vitamin K Antagonism for Prevention of Stroke and Embolism Trial in Atrial Fibrillation.

eFigure 4.4: Impact of class II obesity on AF-related outcomes (sensitivity analysis)

A)

Study or Subgroup	Class II obesity Events	Class II obesity Total	Normal BMI Events	Normal BMI Total	Weight	Risk Ratio M-H, Random, 95% CI	Year
**4.1.1 RCTs**							
Boehringer Ingelheim (RE-LY)	53	2241	181	4697	22.4%	0.61 [0.45, 0.83]	2010
Sandhu et al. (ARISTOTLE)	28	1764	142	4038	12.7%	0.45 [0.30, 0.67]	2016
Balla et al. (ROCKET AF)	55	1898	166	3289	22.8%	0.57 [0.43, 0.77]	2017
Boriani et al. (ENGAGE AF-TIMI 48)	70	2099	273	4491	30.9%	0.55 [0.42, 0.71]	2019
**Subtotal (95% CI)**		**8002**		**16515**	**88.8%**	**0.56 [0.48, 0.65]**	
Total events	206		762				
Heterogeneity: Tau² = 0.00; Chi² = 1.50, df = 3 (P = 0.68); I² = 0%							
Test for overall effect: Z = 7.60 (P < 0.00001)							
**4.1.2 Observational studies**							
Pandey et al. (69-75% VKA use)	21	1158	100	2076	9.5%	0.38 [0.24, 0.60]	2016
Kaplan et al.	5	1421	10	1720	1.8%	0.61 [0.21, 1.77]	2020
**Subtotal (95% CI)**		**2579**		**3796**	**11.2%**	**0.41 [0.26, 0.62]**	
Total events	26		110				
Heterogeneity: Tau² = 0.00; Chi² = 0.64, df = 1 (P = 0.42); I² = 0%							
Test for overall effect: Z = 4.14 (P < 0.0001)							
**Total (95% CI)**		**10581**		**20311**	**100.0%**	**0.54 [0.46, 0.62]**	
Total events	232		872				
Heterogeneity: Tau² = 0.00; Chi² = 3.99, df = 5 (P = 0.55); I² = 0%							
Test for overall effect: Z = 8.55 (P < 0.00001)							
Test for subgroup differences: Chi² = 1.84, df = 1 (P = 0.17), I² = 45.7%							

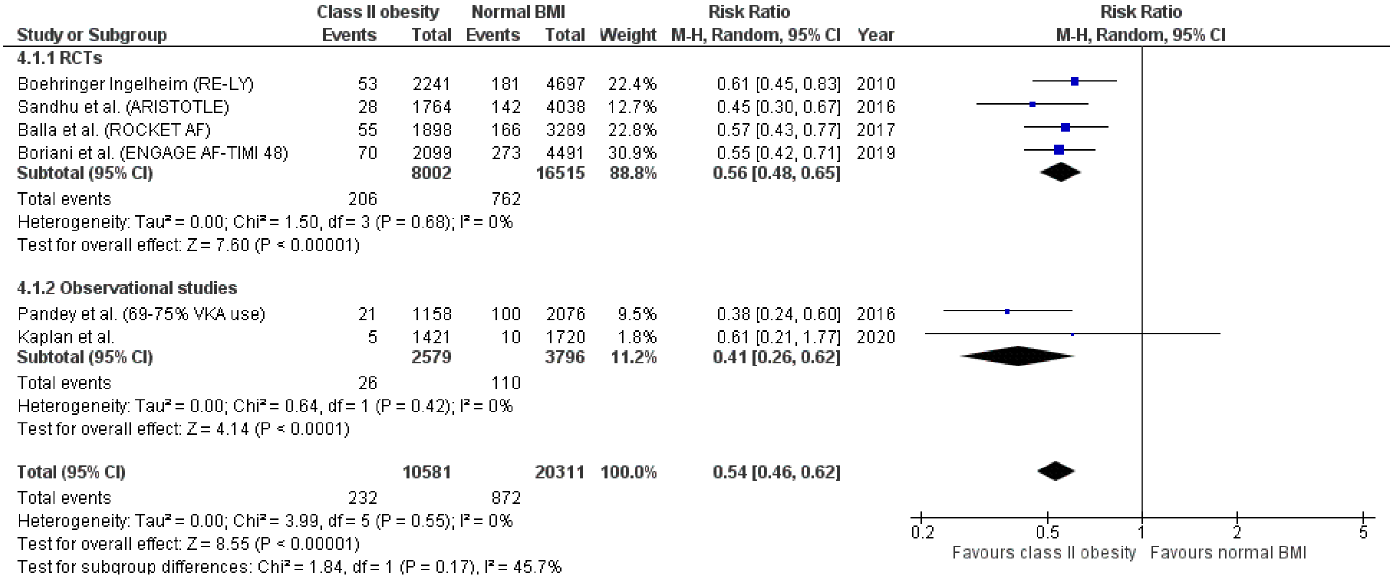

B)

Study or Subgroup	Class II obesity Events	Class II obesity Total	Normal BMI Events	Normal BMI Total	Weight	Risk Ratio M-H, Random, 95% CI	Year
**4.2.1 RCTs**							
Sandhu et al. (ARISTOTLE)	88	1764	397	4038	27.2%	0.51 [0.41, 0.63]	2016
Boriani et al. (ENGAGE AF-TIMI 48)	191	2099	629	4491	42.1%	0.65 [0.56, 0.76]	2019
**Subtotal (95% CI)**		**3863**		**8529**	**69.3%**	**0.58 [0.46, 0.74]**	
Total events	279		1026				
Heterogeneity: Tau² = 0.02; Chi² = 3.20, df = 1 (P = 0.07); I² = 69%							
Test for overall effect: Z = 4.40 (P < 0.0001)							
**4.2.2 Observational studies**							
Pandey et al. (69-75% VKA use)	108	1158	339	2076	30.7%	0.57 [0.47, 0.70]	2016
**Subtotal (95% CI)**		**1158**		**2076**	**30.7%**	**0.57 [0.47, 0.70]**	
Total events	108		339				
Heterogeneity: Not applicable							
Test for overall effect: Z = 5.37 (P < 0.00001)							
**Total (95% CI)**		**5021**		**10605**	**100.0%**	**0.58 [0.51, 0.67]**	
Total events	387		1365				
Heterogeneity: Tau² = 0.01; Chi² = 3.36, df = 2 (P = 0.19); I² = 40%							
Test for overall effect: Z = 7.37 (P < 0.00001)							
Test for subgroup differences: Chi² = 0.01, df = 1 (P = 0.90), I² = 0%							

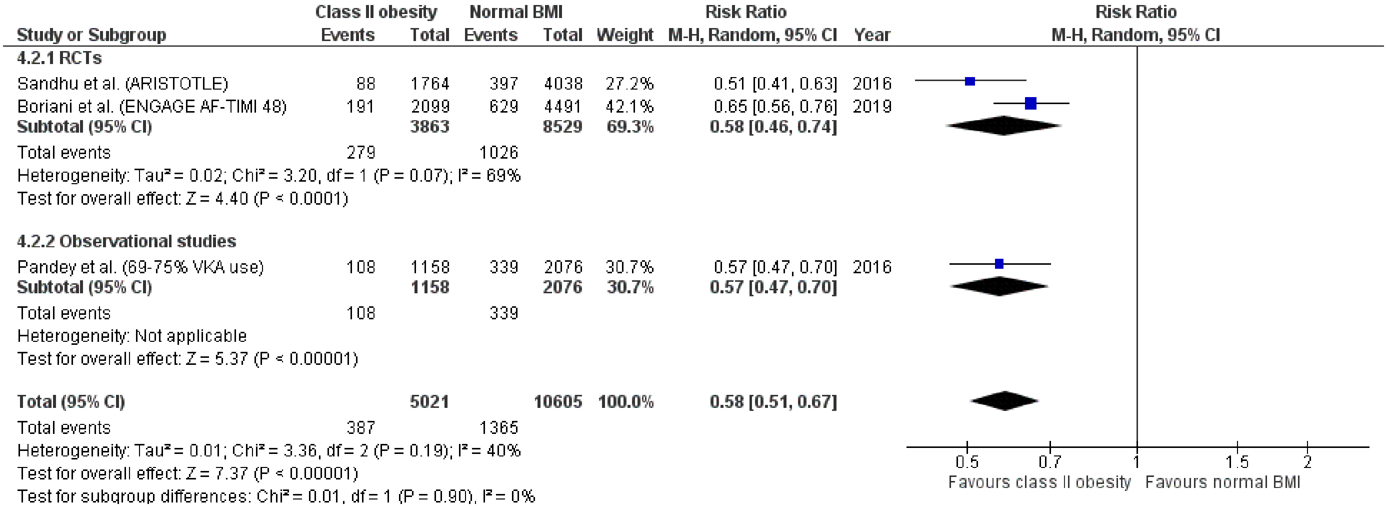

C)

Study or Subgroup	Class II obesity Events	Class II obesity Total	Normal BMI Events	Normal BMI Total	Weight	Risk Ratio M-H, Random, 95% CI	Year
**4.3.1 RCTs**							
Boehringer Ingelheim (RE-LY)	147	2241	344	4697	26.9%	0.90 [0.74, 1.08]	2010
Sandhu et al. (ARISTOTLE)	67	1745	217	3984	15.3%	0.70 [0.54, 0.92]	2016
Balla et al. (ROCKET AF)	106	1896	179	3274	19.1%	1.02 [0.81, 1.29]	2017
Boriani et al. (ENGAGE AF-TIMI 48)	130	2099	283	4491	24.1%	0.98 [0.80, 1.20]	2019
**Subtotal (95% CI)**		**7981**		**16446**	**85.4%**	**0.90 [0.79, 1.04]**	
Total events	450		1023				
Heterogeneity: Tau² = 0.01; Chi² = 5.04, df = 3 (P = 0.17); I² = 40%							
Test for overall effect: Z = 1.38 (P = 0.17)							
**4.3.2 Observational studies**							
Pandey et al. (69-75% VKA use)	71	1158	143	2076	14.6%	0.89 [0.68, 1.17]	2016
**Subtotal (95% CI)**		**1158**		**2076**	**14.6%**	**0.89 [0.68, 1.17]**	
Total events	71		143				
Heterogeneity: Not applicable							
Test for overall effect: Z = 0.83 (P = 0.41)							
**Total (95% CI)**		**9139**		**18522**	**100.0%**	**0.90 [0.81, 1.01]**	
Total events	521		1166				
Heterogeneity: Tau² = 0.00; Chi² = 5.06, df = 4 (P = 0.28); I² = 21%							
Test for overall effect: Z = 1.71 (P = 0.09)							
Test for subgroup differences: Chi² = 0.01, df = 1 (P = 0.92), I² = 0%							

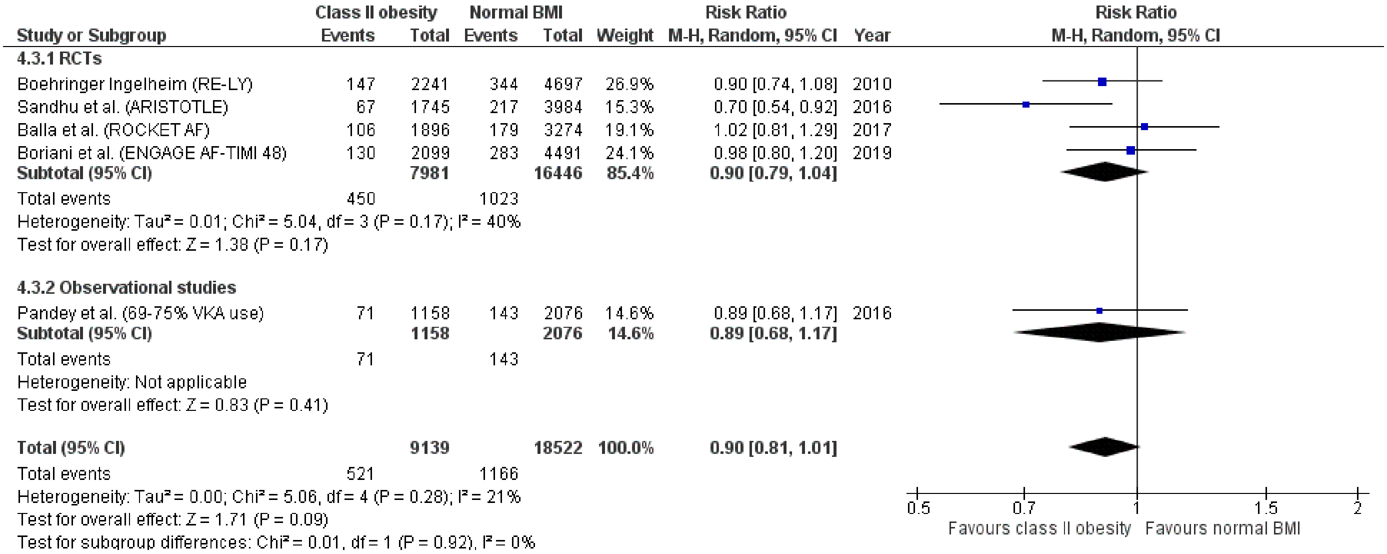

D)

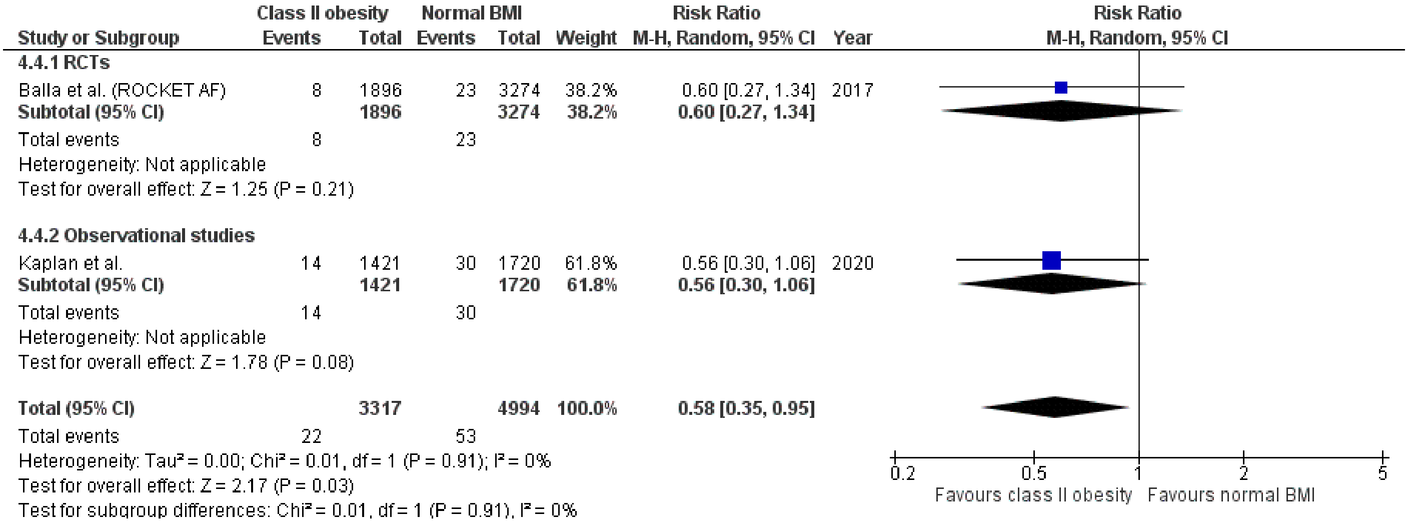

<u>eFigure 4.4:</u> Forest plot of the risk of **A)** stroke or systemic embolism, **B)** all-cause mortality, **C)** major bleeding, and **D)** intracranial bleeding for class II obese (BMI 35-<40 kg/m²) versus normal BMI (18.5-<25 kg/m²) AF patients with and without anticoagulation, categorized according to randomized and observational studies.

AF: atrial fibrillation; ARISTOTLE: the Apixaban for Reduction in Stroke and Other Thromboembolic Events in Atrial Fibrillation trial; BMI: body mass index; CI: confidence interval; ENGAGE AF-TIMI 48: the Effective Anticoagulation with Factor Xa Next Generation in Atrial Fibrillation–Thrombolysis in Myocardial Infarction 48 trial; M-H: Mantel-Haenszel (statistical method); RCT: randomized controlled trial; RE-LY: the Randomized Evaluation of Long-Term Anticoagulation Therapy; ROCKET AF: the Rivaroxaban Once Daily Oral Direct Factor Xa Inhibition Compared with Vitamin K Antagonism for Prevention of Stroke and Embolism Trial in Atrial Fibrillation.

eFigure 4.5: Impact of morbid obesity on AF-related outcomes (sensitivity analysis)

A)

Study or Subgroup	Class III obesity Events	Class III obesity Total	Normal BMI Events	Normal BMI Total	Weight	Risk Ratio M-H, Random, 95% CI	Year
**5.1.1 RCTs**							
Sandhu et al. (ARISTOTLE)	18	1003	142	4038	29.3%	0.51 [0.31, 0.83]	2016
Boriani et al. (ENGAGE AF-TIMI 48)	26	1149	273	4491	43.9%	0.37 [0.25, 0.55]	2019
**Subtotal (95% CI)**		**2152**		**8529**	**73.2%**	**0.42 [0.31, 0.57]**	
Total events	44		415				
Heterogeneity: Tau² = 0.00; Chi² = 0.98, df = 1 (P = 0.32); I² = 0%							
Test for overall effect: Z = 5.50 (P < 0.00001)							
**5.1.2 Observational studies**							
Pandey et al. (69-75% VKA use)	17	942	100	2076	26.8%	0.37 [0.23, 0.62]	2016
**Subtotal (95% CI)**		**942**		**2076**	**26.8%**	**0.37 [0.23, 0.62]**	
Total events	17		100				
Heterogeneity: Not applicable							
Test for overall effect: Z = 3.78 (P = 0.0002)							
**Total (95% CI)**		**3094**		**10605**	**100.0%**	**0.41 [0.31, 0.53]**	
Total events	61		515				
Heterogeneity: Tau² = 0.00; Chi² = 1.13, df = 2 (P = 0.57); I² = 0%							
Test for overall effect: Z = 6.66 (P < 0.00001)							
Test for subgroup differences: Chi² = 0.16, df = 1 (P = 0.69), I² = 0%							

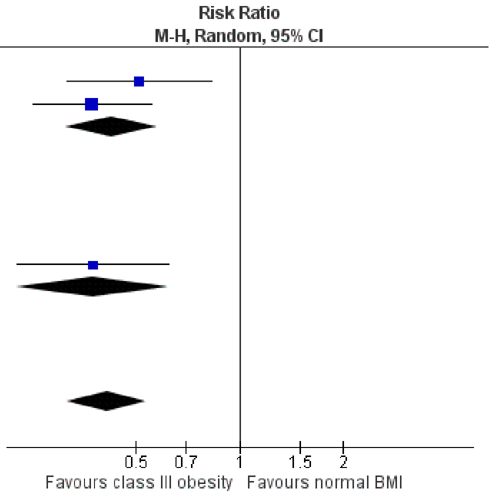

B)

Study or Subgroup	Class III obesity Events	Class III obesity Total	Normal BMI Events	Normal BMI Total	Weight	Risk Ratio M-H, Random, 95% CI	Year
**5.2.1 RCTs**							
Sandhu et al. (ARISTOTLE)	55	1003	397	4038	25.9%	0.56 [0.42, 0.73]	2016
Boriani et al. (ENGAGE AF-TIMI 48)	90	1149	629	4491	42.6%	0.56 [0.45, 0.69]	2019
**Subtotal (95% CI)**		**2152**		**8529**	**68.5%**	**0.56 [0.47, 0.66]**	
Total events	145		1026				
Heterogeneity: Tau² = 0.00; Chi² = 0.00, df = 1 (P = 0.99); I² = 0%							
Test for overall effect: Z = 6.83 (P < 0.00001)							
**5.2.2 Observational studies**							
Pandey et al. (69-75% VKA use)	69	942	339	2076	31.5%	0.45 [0.35, 0.57]	2016
**Subtotal (95% CI)**		**942**		**2076**	**31.5%**	**0.45 [0.35, 0.57]**	
Total events	69		339				
Heterogeneity: Not applicable							
Test for overall effect: Z = 6.36 (P < 0.00001)							
**Total (95% CI)**		**3094**		**10605**	**100.0%**	**0.52 [0.45, 0.60]**	
Total events	214		1365				
Heterogeneity: Tau² = 0.00; Chi² = 2.08, df = 2 (P = 0.35); I² = 4%							
Test for overall effect: Z = 9.03 (P < 0.00001)							
Test for subgroup differences: Chi² = 2.08, df = 1 (P = 0.15), I² = 51.9%							

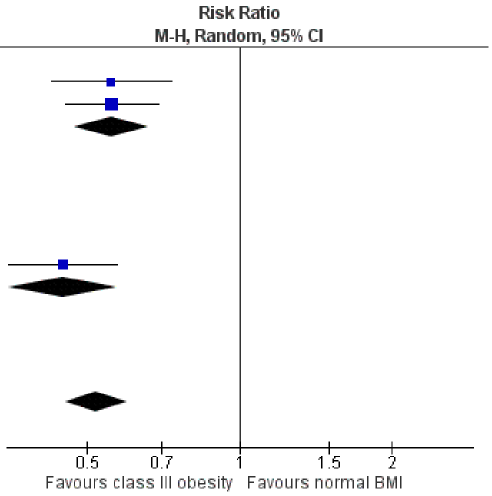

C)

Study or Subgroup	Class III obesity Events	Class III obesity Total	Normal BMI Events	Normal BMI Total	Weight	Risk Ratio M-H, Random, 95% CI	Year
**5.3.1 RCTs**							
Sandhu et al. (ARISTOTLE)	28	997	217	3984	29.3%	0.52 [0.35, 0.76]	2016
Boriani et al. (ENGAGE AF-TIMI 48)	73	1149	283	4491	36.5%	1.01 [0.79, 1.29]	2019
**Subtotal (95% CI)**		**2146**		**8475**	**65.9%**	**0.73 [0.38, 1.42]**	
Total events	101		500				
Heterogeneity: Tau² = 0.20; Chi² = 8.28, df = 1 (P = 0.004); I² = 88%							
Test for overall effect: Z = 0.92 (P = 0.36)							
**5.3.2 Observational studies**							
Pandey et al. (69-75% VKA use)	58	942	143	2076	34.1%	0.89 [0.67, 1.20]	2016
**Subtotal (95% CI)**		**942**		**2076**	**34.1%**	**0.89 [0.67, 1.20]**	
Total events	58		143				
Heterogeneity: Not applicable							
Test for overall effect: Z = 0.74 (P = 0.46)							
**Total (95% CI)**		**3088**		**10551**	**100.0%**	**0.79 [0.55, 1.14]**	
Total events	159		643				
Heterogeneity: Tau² = 0.08; Chi² = 8.42, df = 2 (P = 0.01); I² = 76%							
Test for overall effect: Z = 1.24 (P = 0.21)							
Test for subgroup differences: Chi² = 0.29, df = 1 (P = 0.59), I² = 0%							

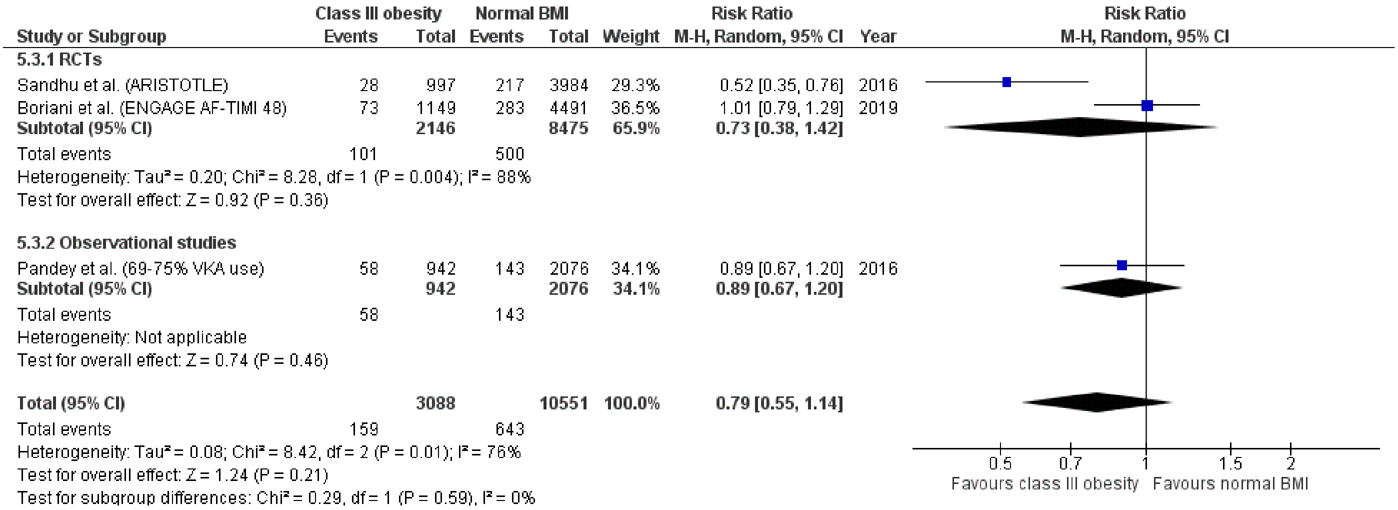

eFigure 4.5: Forest plot of the risk of **A)** stroke or systemic embolism, **B)** all-cause mortality and **C)** major bleeding for anticoagulated morbidly obese (BMI ≥40 kg/m²) versus normal BMI (18.5-<25 kg/m²) AF patients with and without anticoagulation, based on results from randomized studies (no data regarding intracranial bleeding risk).

AF: atrial fibrillation; ARISTOTLE: the Apixaban for Reduction in Stroke and Other Thromboembolic Events in Atrial Fibrillation trial; BMI: body mass index; CI: confidence interval; ENGAGE AF-TIMI 48: the Effective Anticoagulation with Factor Xa Next Generation in Atrial Fibrillation–Thrombolysis in Myocardial Infarction 48 trial; M-H: Mantel-Haenszel (statistical method); RCT: randomized controlled trial.

eFigure 5: Sensitivity analysis for mortality in underweight versus normal BMI AF patients

A)

Study or Subgroup	Underweight Events	Underweight Total	Normal BMI Events	Normal BMI Total	Weight	Risk Ratio M-H, Random, 95% CI	Year
**1.2.1 RCTs**							
Boriani et al. (ENGAGE AF-TIMI 48)	66	177	629	4491	36.2%	2.66 [2.17, 3.27]	2019
**Subtotal (95% CI)**		**177**		**4491**	**36.2%**	**2.66 [2.17, 3.27]**	
Total events	66		629				
Heterogeneity: Not applicable							
Test for overall effect: Z = 9.39 (P < 0.00001)							
**1.2.2 Observational studies**							
Park et al.	6	62	6	753	0.0%	12.15 [4.04, 36.54]	2017
Murakawa et al.	44	542	72	4410	26.6%	4.97 [3.45, 7.16]	2020
Lee et al.	114	1154	822	22507	37.2%	2.70 [2.24, 3.26]	2021
**Subtotal (95% CI)**		**1696**		**26917**	**63.8%**	**3.59 [1.98, 6.51]**	
Total events	158		894				
Heterogeneity: Tau² = 0.16; Chi² = 8.50, df = 1 (P = 0.004); I² = 88%							
Test for overall effect: Z = 4.21 (P < 0.0001)							
**Total (95% CI)**		**1873**		**31408**	**100.0%**	**3.16 [2.34, 4.28]**	
Total events	224		1523				
Heterogeneity: Tau² = 0.06; Chi² = 9.66, df = 2 (P = 0.008); I² = 79%							
Test for overall effect: Z = 7.44 (P < 0.00001)							
Test for subgroup differences: Chi² = 0.87, df = 1 (P = 0.35), I² = 0%							

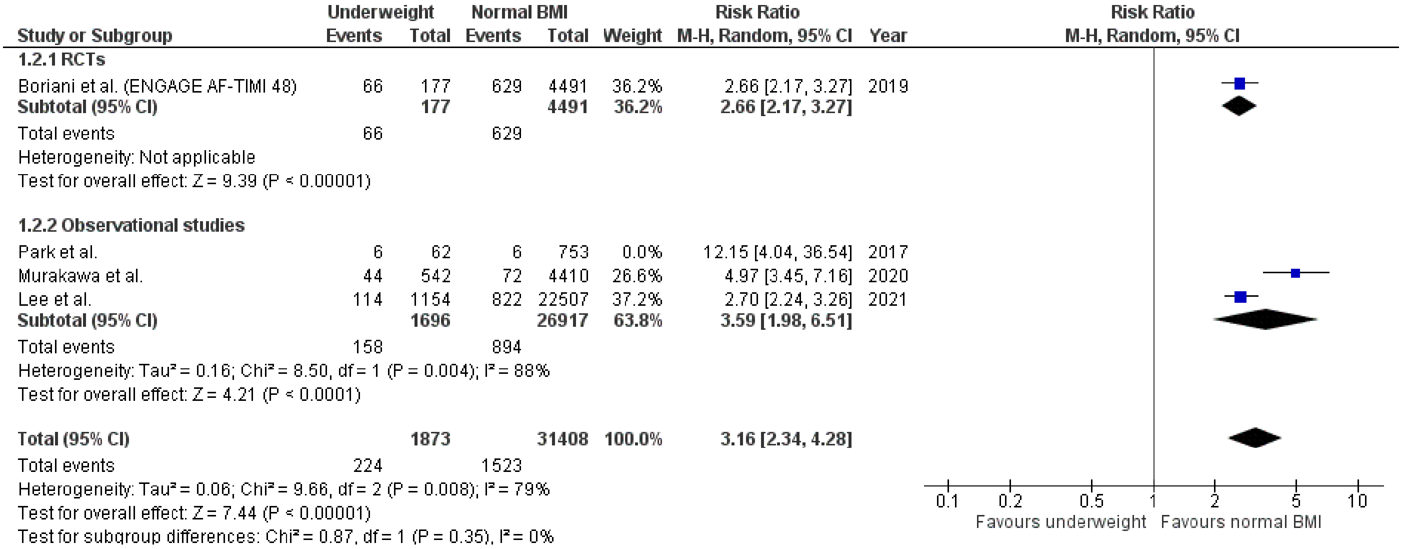

B)

Study or Subgroup	Underweight Events	Underweight Total	Normal BMI Events	Normal BMI Total	Weight	Risk Ratio M-H, Random, 95% CI	Year
**1.2.1 RCTs**							
Boriani et al. (ENGAGE AF-TIMI 48)	66	177	629	4491	45.6%	2.66 [2.17, 3.27]	2019
**Subtotal (95% CI)**		**177**		**4491**	**45.6%**	**2.66 [2.17, 3.27]**	
Total events	66		629				
Heterogeneity: Not applicable							
Test for overall effect: Z = 9.39 (P < 0.00001)							
**1.2.2 Observational studies**							
Park et al.	6	62	6	753	7.4%	12.15 [4.04, 36.54]	2017
Murakawa et al.	44	542	72	4410	0.0%	4.97 [3.45, 7.16]	2020
Lee et al.	114	1154	822	22507	47.0%	2.70 [2.24, 3.26]	2021
**Subtotal (95% CI)**		**1216**		**23260**	**54.4%**	**5.18 [1.20, 22.25]**	
Total events	120		828				
Heterogeneity: Tau² = 0.97; Chi² = 6.94, df = 1 (P = 0.008); I² = 86%							
Test for overall effect: Z = 2.21 (P = 0.03)							
**Total (95% CI)**		**1393**		**27751**	**100.0%**	**3.00 [2.17, 4.15]**	
Total events	186		1457				
Heterogeneity: Tau² = 0.05; Chi² = 7.11, df = 2 (P = 0.03); I² = 72%							
Test for overall effect: Z = 6.67 (P < 0.00001)							
Test for subgroup differences: Chi² = 0.78, df = 1 (P = 0.38), I² = 0%							

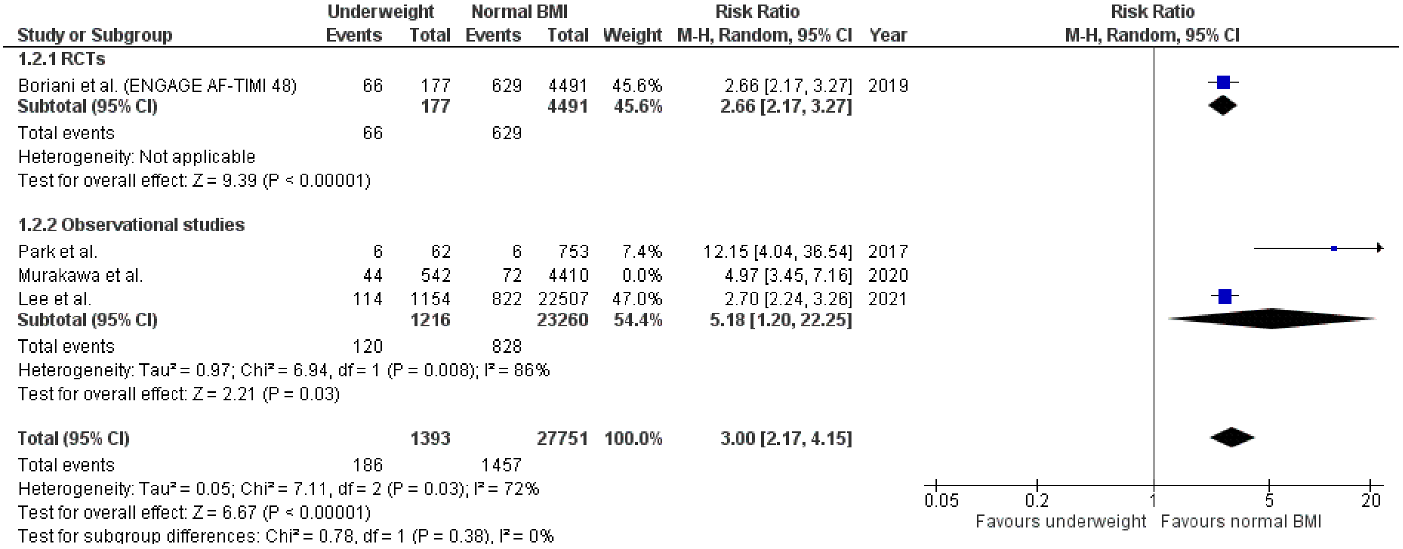

C)

Study or Subgroup	Underweight Events	Underweight Total	Normal BMI Events	Normal BMI Total	Weight	Risk Ratio M-H, Random, 95% CI	Year
**1.2.1 RCTs**							
Boriani et al. (ENGAGE AF-TIMI 48)	66	177	629	4491	41.9%	2.66 [2.17, 3.27]	2019
**Subtotal (95% CI)**		**177**		**4491**	**41.9%**	**2.66 [2.17, 3.27]**	
Total events	66		629				
Heterogeneity: Not applicable							
Test for overall effect: Z = 9.39 (P < 0.00001)							
**1.2.2 Observational studies**							
Park et al.	6	62	6	753	19.6%	12.15 [4.04, 36.54]	2017
Murakawa et al.	44	542	72	4410	38.5%	4.97 [3.45, 7.16]	2020
Lee et al.	114	1154	822	22507	0.0%	2.70 [2.24, 3.26]	2021
**Subtotal (95% CI)**		**604**		**5163**	**58.1%**	**6.64 [2.92, 15.08]**	
Total events	50		78				
Heterogeneity: $\text{Tau}^2 = 0.22$; $\text{Chi}^2 = 2.28$, df = 1 (P = 0.13); $I^2 = 56\%$							
Test for overall effect: Z = 4.53 (P < 0.00001)							
**Total (95% CI)**		**781**		**9654**	**100.0%**	**4.56 [2.36, 8.80]**	
Total events	116		707				
Heterogeneity: $\text{Tau}^2 = 0.26$; $\text{Chi}^2 = 14.67$, df = 2 (P = 0.0007); $I^2 = 86\%$							
Test for overall effect: Z = 4.52 (P < 0.00001)							
Test for subgroup differences: $\text{Chi}^2 = 4.49$, df = 1 (P = 0.03), $I^2 = 77.7\%$							

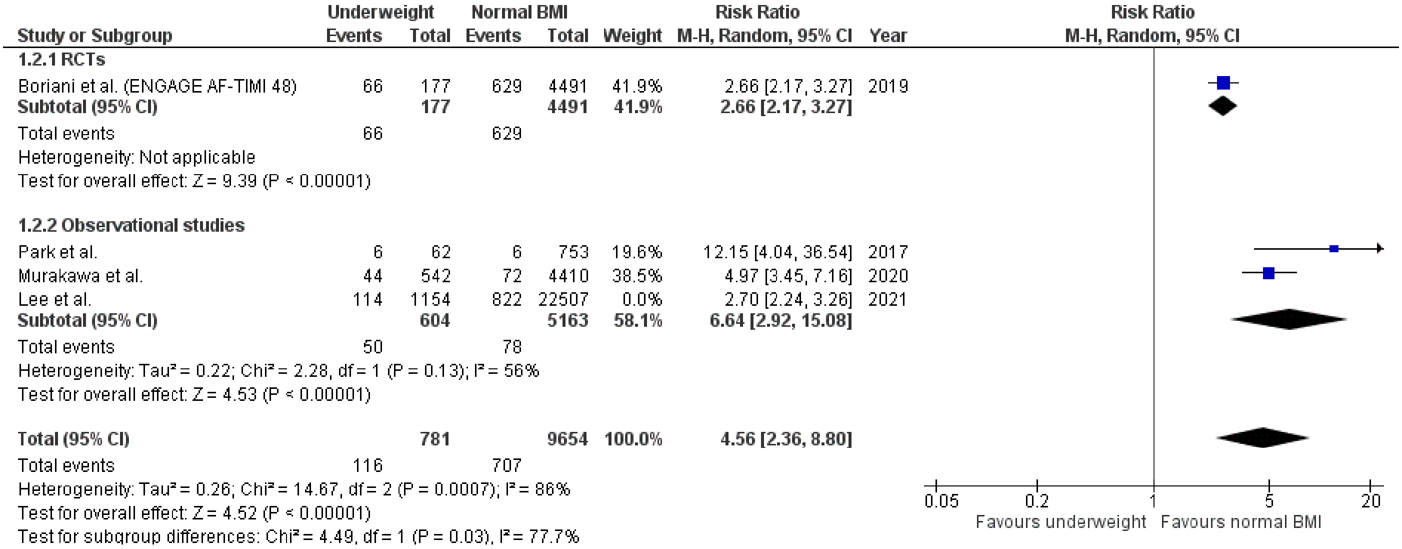

D)

Study or Subgroup	Underweight Events	Underweight Total	Normal BMI Events	Normal BMI Total	Weight	Risk Ratio M-H, Random, 95% CI	Year
**1.2.1 RCTs**							
Boriani et al. (ENGAGE AF-TIMI 48)	66	177	629	4491	45.5%	2.66 [2.17, 3.27]	2019
**Subtotal (95% CI)**		**177**		**4491**	**45.5%**	**2.66 [2.17, 3.27]**	
Total events	66		629				
Heterogeneity: Not applicable							
Test for overall effect: Z = 9.39 (P < 0.00001)							
**1.2.2 Observational studies**							
Park et al.	6	62	6	753	0.0%	12.15 [4.04, 36.54]	2017
Murakawa et al.	44	542	72	4410	0.0%	4.97 [3.45, 7.16]	2020
Lee et al.	114	1154	822	22507	54.5%	2.70 [2.24, 3.26]	2021
**Subtotal (95% CI)**		**1154**		**22507**	**54.5%**	**2.70 [2.24, 3.26]**	
Total events	114		822				
Heterogeneity: Not applicable							
Test for overall effect: Z = 10.44 (P < 0.00001)							
**Total (95% CI)**		**1331**		**26998**	**100.0%**	**2.69 [2.34, 3.08]**	
Total events	180		1451				
Heterogeneity: $\text{Tau}^2 = 0.00$; $\text{Chi}^2 = 0.01$, df = 1 (P = 0.91); $I^2 = 0\%$							
Test for overall effect: Z = 14.05 (P < 0.00001)							
Test for subgroup differences: $\text{Chi}^2 = 0.01$, df = 1 (P = 0.91), $I^2 = 0\%$							

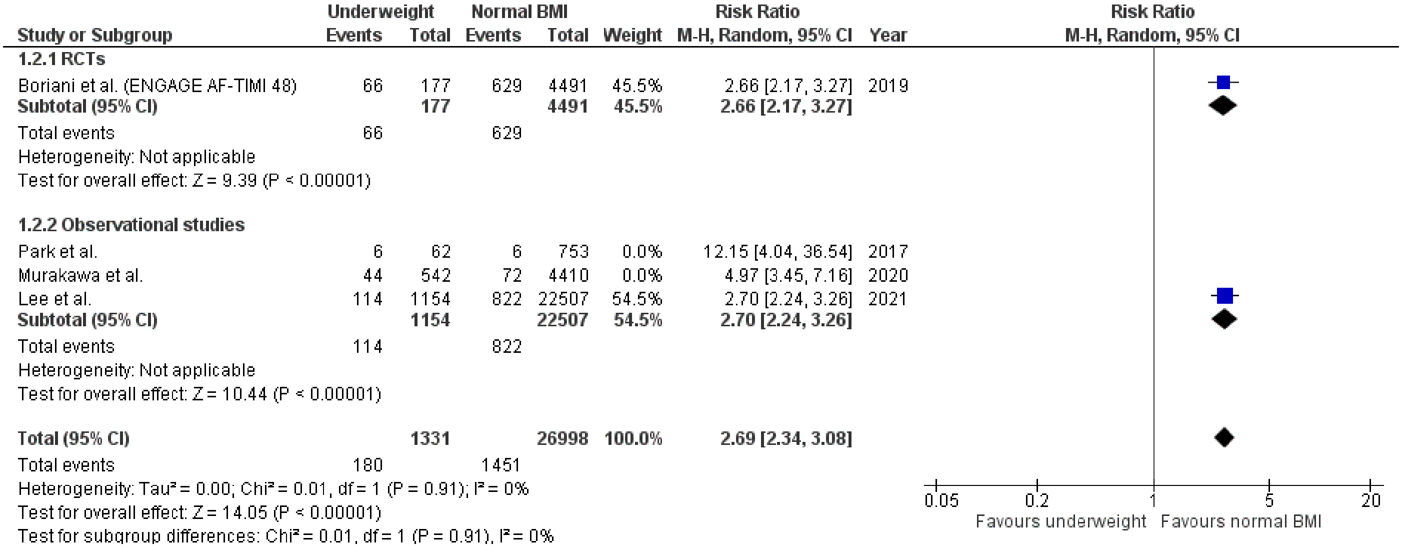

E)

Study or Subgroup	Underweight Events	Underweight Total	Normal BMI Events	Normal BMI Total	Weight	Risk Ratio M-H, Random, 95% CI	Year
**1.2.1 RCTs**							
Boriani et al. (ENGAGE AF-TIMI 48)	66	177	629	4491	52.9%	2.66 [2.17, 3.27]	2019
**Subtotal (95% CI)**		**177**		**4491**	**52.9%**	**2.66 [2.17, 3.27]**	
Total events	66		629				
Heterogeneity: Not applicable							
Test for overall effect: Z = 9.39 (P < 0.00001)							
**1.2.2 Observational studies**							
Park et al.	6	62	6	753	0.0%	12.15 [4.04, 36.54]	2017
Murakawa et al.	44	542	72	4410	47.1%	4.97 [3.45, 7.16]	2020
Lee et al.	114	1154	822	22507	0.0%	2.70 [2.24, 3.26]	2021
**Subtotal (95% CI)**		**542**		**4410**	**47.1%**	**4.97 [3.45, 7.16]**	
Total events	44		72				
Heterogeneity: Not applicable							
Test for overall effect: Z = 8.63 (P < 0.00001)							
**Total (95% CI)**		**719**		**8901**	**100.0%**	**3.57 [1.92, 6.64]**	
Total events	110		701				
Heterogeneity: $\text{Tau}^2 = 0.18$; $\text{Chi}^2 = 8.84$, df = 1 (P = 0.003); $I^2 = 89\%$							
Test for overall effect: Z = 4.02 (P < 0.0001)							
Test for subgroup differences: $\text{Chi}^2 = 8.59$, df = 1 (P = 0.003), $I^2 = 88.4\%$							

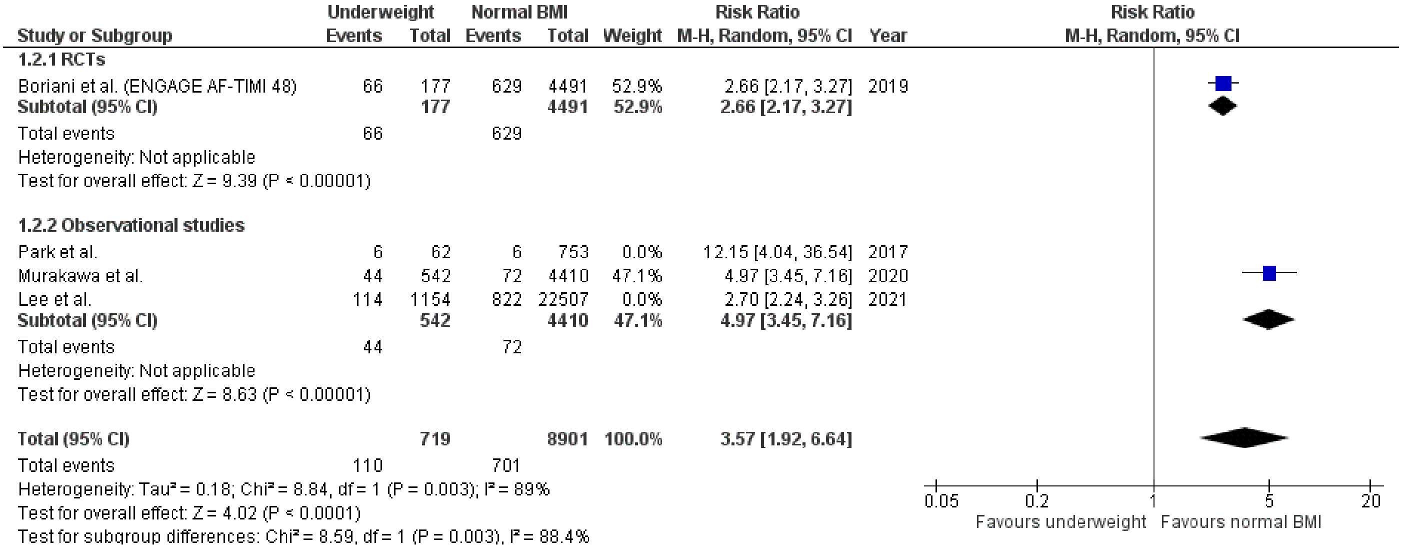

F)

Study or Subgroup	Underweight Events	Underweight Total	Normal BMI Events	Normal BMI Total	Weight	Risk Ratio M-H, Random, 95% CI	Year
**1.2.1 RCTs**							
Boriani et al. (ENGAGE AF-TIMI 48)	66	177	629	4491	56.6%	2.66 [2.17, 3.27]	2019
**Subtotal (95% CI)**		**177**		**4491**	**56.6%**	**2.66 [2.17, 3.27]**	
Total events	66		629				
Heterogeneity: Not applicable							
Test for overall effect: Z = 9.39 (P < 0.00001)							
**1.2.2 Observational studies**							
Park et al.	6	62	6	753	43.4%	12.15 [4.04, 36.54]	2017
Murakawa et al.	44	542	72	4410	0.0%	4.97 [3.45, 7.16]	2020
Lee et al.	114	1154	822	22507	0.0%	2.70 [2.24, 3.26]	2021
**Subtotal (95% CI)**		**62**		**753**	**43.4%**	**12.15 [4.04, 36.54]**	
Total events	6		6				
Heterogeneity: Not applicable							
Test for overall effect: Z = 4.44 (P < 0.00001)							
**Total (95% CI)**		**239**		**5244**	**100.0%**	**5.14 [1.18, 22.52]**	
Total events	72		635				
Heterogeneity: Tau² = 0.99; Chi² = 7.07, df = 1 (P = 0.008); I² = 86%							
Test for overall effect: Z = 2.17 (P = 0.03)							
Test for subgroup differences: Chi² = 7.05, df = 1 (P = 0.008), I² = 85.8%							

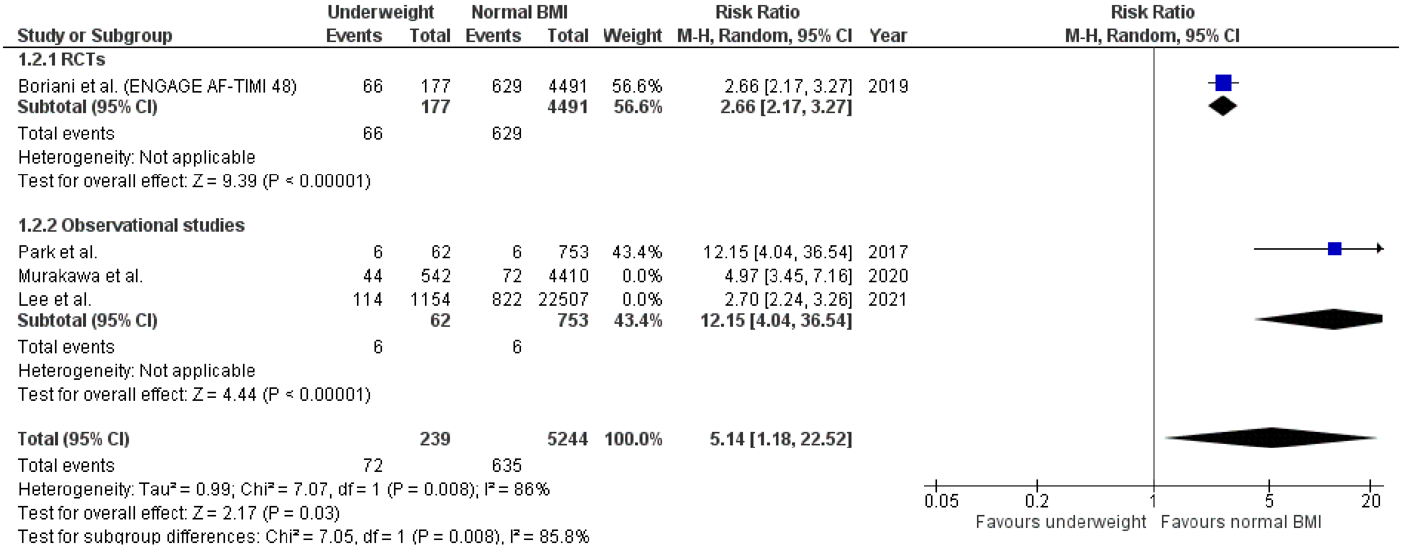

eFigure 5: Forest plot of the risk of all-cause mortality for underweight (BMI <18.5 kg/m²) versus normal BMI (18.5-<25 kg/m²) AF patients receiving anticoagulation, after one-by-one exclusion of the results of the observational studies.

AF: atrial fibrillation; BMI: body mass index; CI: confidence interval; ENGAGE AF-TIMI 48: the Effective Anticoagulation with Factor Xa Next Generation in Atrial Fibrillation–Thrombolysis in Myocardial Infarction 48 trial; M-H: Mantel-Haenszel (statistical method); RCT: randomized controlled trial.

eFigure 6: Sensitivity analysis for major bleeding in underweight versus normal BMI AF patients

A)

Study or Subgroup	Underweight Events	Underweight Total	Normal BMI Events	Normal BMI Total	Weight	Risk Ratio M-H, Random, 95% CI	Year
**1.3.1 RCTs**							
Boriani et al. (ENGAGE AF-TIMI 48)	17	177	283	4491	38.9%	1.52 [0.96, 2.43]	2019
**Subtotal (95% CI)**		**177**		**4491**	**38.9%**	**1.52 [0.96, 2.43]**	
Total events	17		283				
Heterogeneity: Not applicable							
Test for overall effect: Z = 1.77 (P = 0.08)							
**1.3.2 Observational studies**							
Park et al.	6	62	16	753	0.0%	4.55 [1.85, 11.22]	2017
Murakawa et al.	9	542	70	4410	30.7%	1.05 [0.53, 2.08]	2020
Lee et al.	8	1154	283	22507	30.3%	0.55 [0.27, 1.11]	2021
**Subtotal (95% CI)**		**1696**		**26917**	**61.1%**	**0.76 [0.40, 1.44]**	
Total events	17		353				
Heterogeneity: $Tau^2 = 0.08$; $Chi^2 = 1.67$, df = 1 (P = 0.20); $I^2 = 40\%$							
Test for overall effect: Z = 0.84 (P = 0.40)							
**Total (95% CI)**		**1873**		**31408**	**100.0%**	**1.00 [0.54, 1.84]**	
Total events	34		636				
Heterogeneity: $Tau^2 = 0.19$; $Chi^2 = 5.98$, df = 2 (P = 0.05); $I^2 = 67\%$							
Test for overall effect: Z = 0.01 (P = 0.99)							
Test for subgroup differences: $Chi^2 = 2.98$, df = 1 (P = 0.08), $I^2 = 66.4\%$							

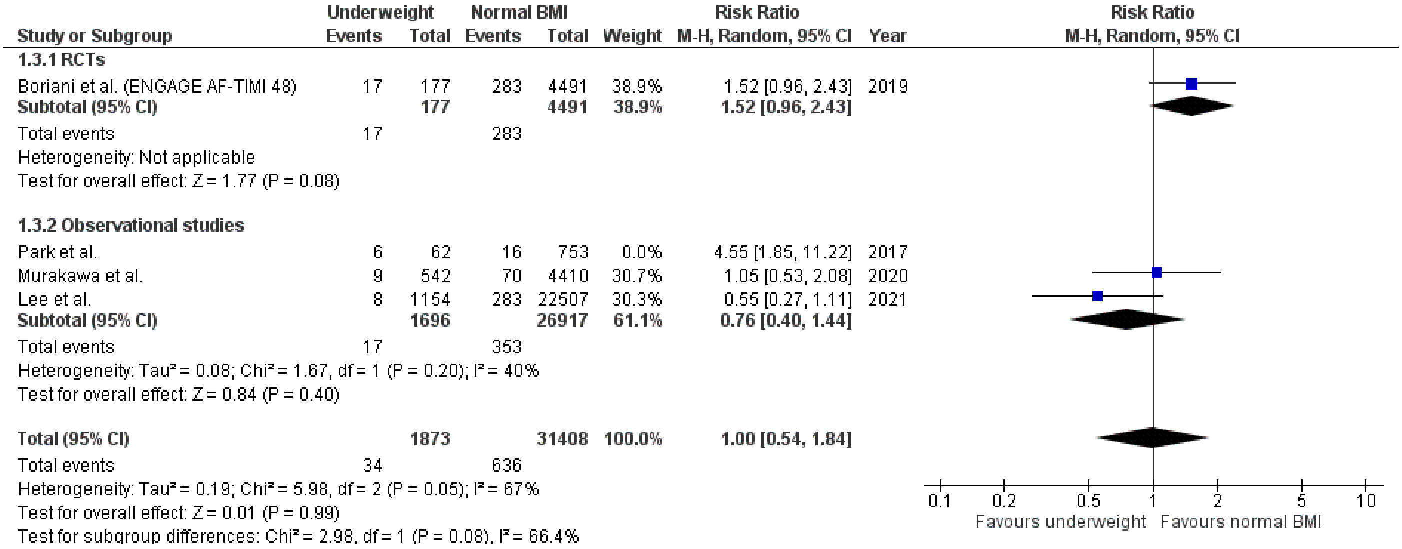

B)

Study or Subgroup	Underweight Events	Underweight Total	Normal BMI Events	Normal BMI Total	Weight	Risk Ratio M-H, Random, 95% CI	Year
**1.3.1 RCTs**							
Boriani et al. (ENGAGE AF-TIMI 48)	17	177	283	4491	36.3%	1.52 [0.96, 2.43]	2019
**Subtotal (95% CI)**		**177**		**4491**	**36.3%**	**1.52 [0.96, 2.43]**	
Total events	17		283				
Heterogeneity: Not applicable							
Test for overall effect: Z = 1.77 (P = 0.08)							
**1.3.2 Observational studies**							
Park et al.	6	62	16	753	30.4%	4.55 [1.85, 11.22]	2017
Murakawa et al.	9	542	70	4410	0.0%	1.05 [0.53, 2.08]	2020
Lee et al.	8	1154	283	22507	33.3%	0.55 [0.27, 1.11]	2021
**Subtotal (95% CI)**		**1216**		**23260**	**63.7%**	**1.56 [0.18, 13.70]**	
Total events	14		299				
Heterogeneity: $Tau^2 = 2.29$; $Chi^2 = 14.52$, df = 1 (P = 0.0001); $I^2 = 93\%$							
Test for overall effect: Z = 0.40 (P = 0.69)							
**Total (95% CI)**		**1393**		**27751**	**100.0%**	**1.51 [0.53, 4.34]**	
Total events	31		582				
Heterogeneity: $Tau^2 = 0.74$; $Chi^2 = 14.63$, df = 2 (P = 0.0007); $I^2 = 86\%$							
Test for overall effect: Z = 0.77 (P = 0.44)							
Test for subgroup differences: $Chi^2 = 0.00$, df = 1 (P = 0.99), $I^2 = 0\%$							

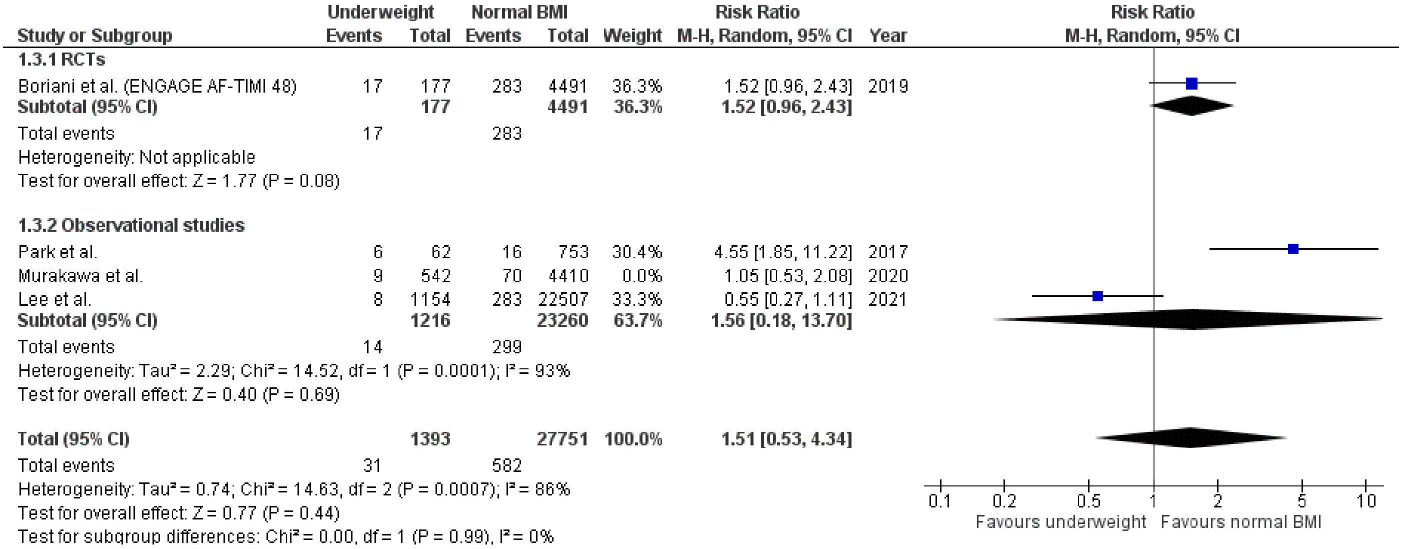

C)

Study or Subgroup	Underweight Events	Underweight Total	Normal BMI Events	Normal BMI Total	Weight	Risk Ratio M-H, Random, 95% CI	Year
**1.3.1 RCTs**							
Boriani et al. (ENGAGE AF-TIMI 48)	17	177	283	4491	39.8%	1.52 [0.96, 2.43]	2019
**Subtotal (95% CI)**		**177**		**4491**	**39.8%**	**1.52 [0.96, 2.43]**	
Total events	17		283				
Heterogeneity: Not applicable							
Test for overall effect: Z = 1.77 (P = 0.08)							
**1.3.2 Observational studies**							
Park et al.	6	62	16	753	27.1%	4.55 [1.85, 11.22]	2017
Murakawa et al.	9	542	70	4410	33.1%	1.05 [0.53, 2.08]	2020
Lee et al.	8	1154	283	22507	0.0%	0.55 [0.27, 1.11]	2021
**Subtotal (95% CI)**		**604**		**5163**	**60.2%**	**2.12 [0.48, 9.30]**	
Total events	15		86				
Heterogeneity: Tau² = 0.97; Chi² = 6.80, df = 1 (P = 0.009); I² = 85%							
Test for overall effect: Z = 1.00 (P = 0.32)							
**Total (95% CI)**		**781**		**9654**	**100.0%**	**1.81 [0.89, 3.69]**	
Total events	32		369				
Heterogeneity: Tau² = 0.28; Chi² = 6.80, df = 2 (P = 0.03); I² = 71%							
Test for overall effect: Z = 1.63 (P = 0.10)							
Test for subgroup differences: Chi² = 0.17, df = 1 (P = 0.68), I² = 0%							

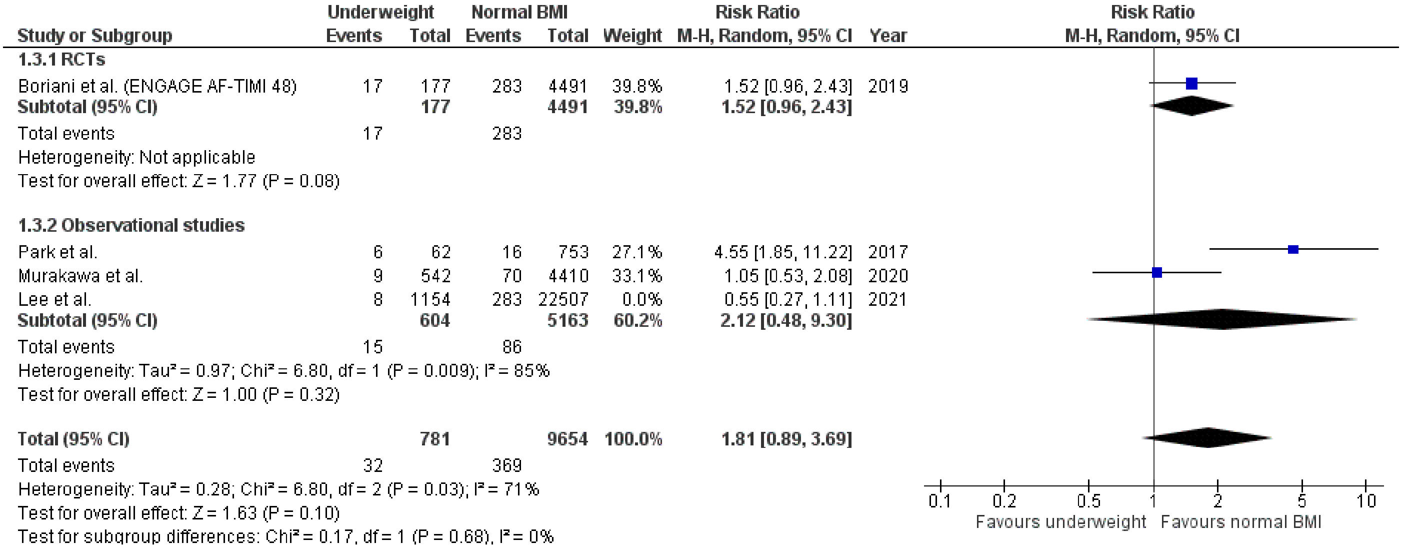

D)

Study or Subgroup	Underweight Events	Underweight Total	Normal BMI Events	Normal BMI Total	Weight	Risk Ratio M-H, Random, 95% CI	Year
**1.3.1 RCTs**							
Boriani et al. (ENGAGE AF-TIMI 48)	17	177	283	4491	53.2%	1.52 [0.96, 2.43]	2019
**Subtotal (95% CI)**		**177**		**4491**	**53.2%**	**1.52 [0.96, 2.43]**	
Total events	17		283				
Heterogeneity: Not applicable							
Test for overall effect: Z = 1.77 (P = 0.08)							
**1.3.2 Observational studies**							
Park et al.	6	62	16	753	0.0%	4.55 [1.85, 11.22]	2017
Murakawa et al.	9	542	70	4410	0.0%	1.05 [0.53, 2.08]	2020
Lee et al.	8	1154	283	22507	46.8%	0.55 [0.27, 1.11]	2021
**Subtotal (95% CI)**		**1154**		**22507**	**46.8%**	**0.55 [0.27, 1.11]**	
Total events	8		283				
Heterogeneity: Not applicable							
Test for overall effect: Z = 1.67 (P = 0.10)							
**Total (95% CI)**		**1331**		**26998**	**100.0%**	**0.95 [0.34, 2.66]**	
Total events	25		566				
Heterogeneity: Tau² = 0.47; Chi² = 6.07, df = 1 (P = 0.01); I² = 84%							
Test for overall effect: Z = 0.10 (P = 0.92)							
Test for subgroup differences: Chi² = 5.62, df = 1 (P = 0.02), I² = 82.2%							

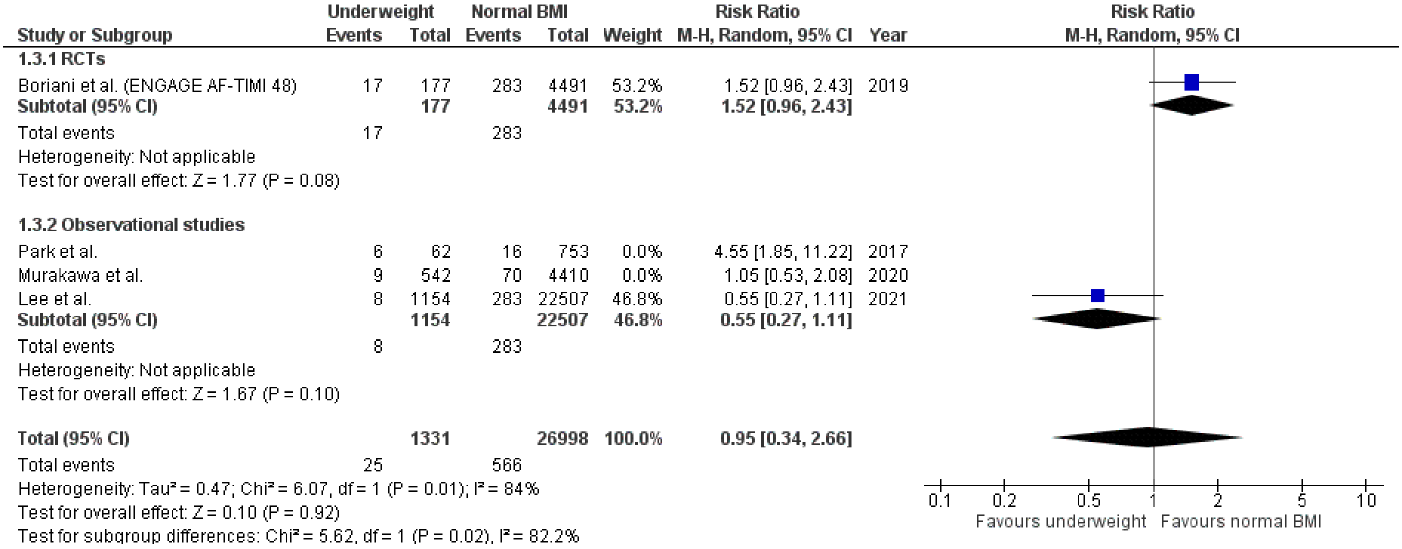

E)

Study or Subgroup	Underweight Events	Underweight Total	Normal BMI Events	Normal BMI Total	Weight	Risk Ratio M-H, Random, 95% CI	Year
**1.3.1 RCTs**							
Boriani et al. (ENGAGE AF-TIMI 48)	17	177	283	4491	68.6%	1.52 [0.96, 2.43]	2019
**Subtotal (95% CI)**		**177**		**4491**	**68.6%**	**1.52 [0.96, 2.43]**	
Total events	17		283				
Heterogeneity: Not applicable							
Test for overall effect: Z = 1.77 (P = 0.08)							
**1.3.2 Observational studies**							
Park et al.	6	62	16	753	0.0%	4.55 [1.85, 11.22]	2017
Murakawa et al.	9	542	70	4410	31.4%	1.05 [0.53, 2.08]	2020
Lee et al.	8	1154	283	22507	0.0%	0.55 [0.27, 1.11]	2021
**Subtotal (95% CI)**		**542**		**4410**	**31.4%**	**1.05 [0.53, 2.08]**	
Total events	9		70				
Heterogeneity: Not applicable							
Test for overall effect: Z = 0.13 (P = 0.90)							
**Total (95% CI)**		**719**		**8901**	**100.0%**	**1.35 [0.92, 1.99]**	
Total events	26		353				
Heterogeneity: Tau² = 0.00; Chi² = 0.80, df = 1 (P = 0.37); I² = 0%							
Test for overall effect: Z = 1.54 (P = 0.12)							
Test for subgroup differences: Chi² = 0.79, df = 1 (P = 0.37), I² = 0%							

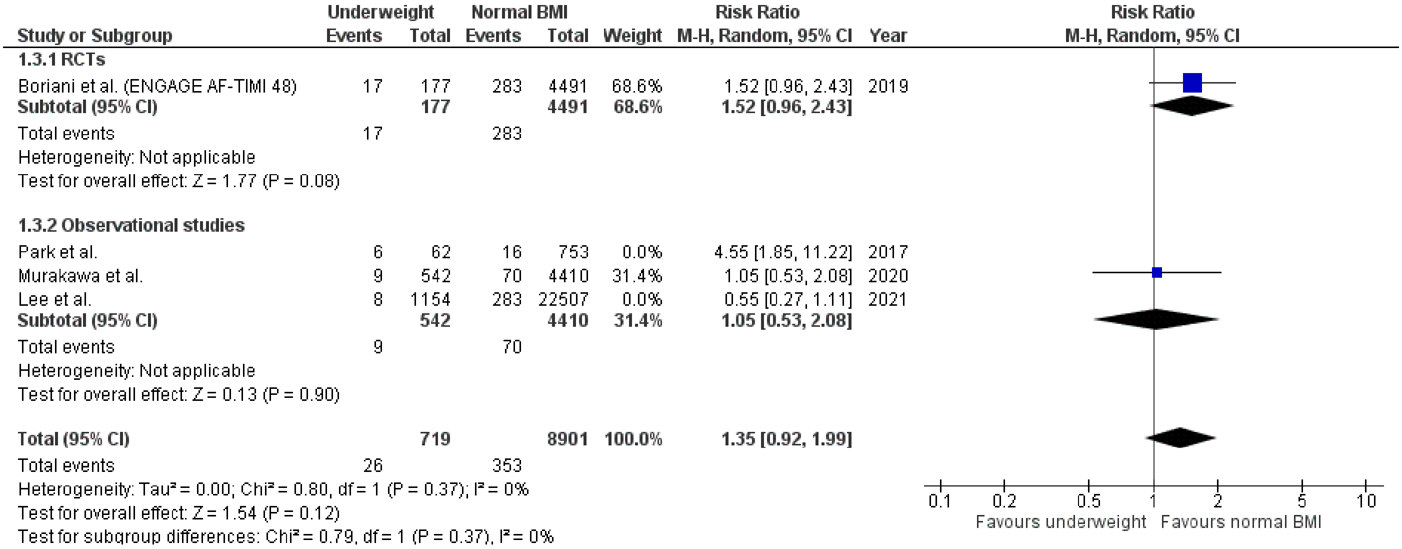

F)

Study or Subgroup	Underweight Events	Underweight Total	Normal BMI Events	Normal BMI Total	Weight	Risk Ratio M-H, Random, 95% CI	Year
**1.3.1 RCTs**							
Boriani et al. (ENGAGE AF-TIMI 48)	17	177	283	4491	56.4%	1.52 [0.96, 2.43]	2019
**Subtotal (95% CI)**		**177**		**4491**	**56.4%**	**1.52 [0.96, 2.43]**	
Total events	17		283				
Heterogeneity: Not applicable							
Test for overall effect: Z = 1.77 (P = 0.08)							
**1.3.2 Observational studies**							
Park et al.	6	62	16	753	43.6%	4.55 [1.85, 11.22]	2017
Murakawa et al.	9	542	70	4410	0.0%	1.05 [0.53, 2.08]	2020
Lee et al.	8	1154	283	22507	0.0%	0.55 [0.27, 1.11]	2021
**Subtotal (95% CI)**		**62**		**753**	**43.6%**	**4.55 [1.85, 11.22]**	
Total events	6		16				
Heterogeneity: Not applicable							
Test for overall effect: Z = 3.30 (P = 0.0010)							
**Total (95% CI)**		**239**		**5244**	**100.0%**	**2.46 [0.84, 7.16]**	
Total events	23		299				
Heterogeneity: Tau² = 0.47; Chi² = 4.52, df = 1 (P = 0.03); I² = 78%							
Test for overall effect: Z = 1.65 (P = 0.10)							
Test for subgroup differences: Chi² = 4.47, df = 1 (P = 0.03), I² = 77.6%							

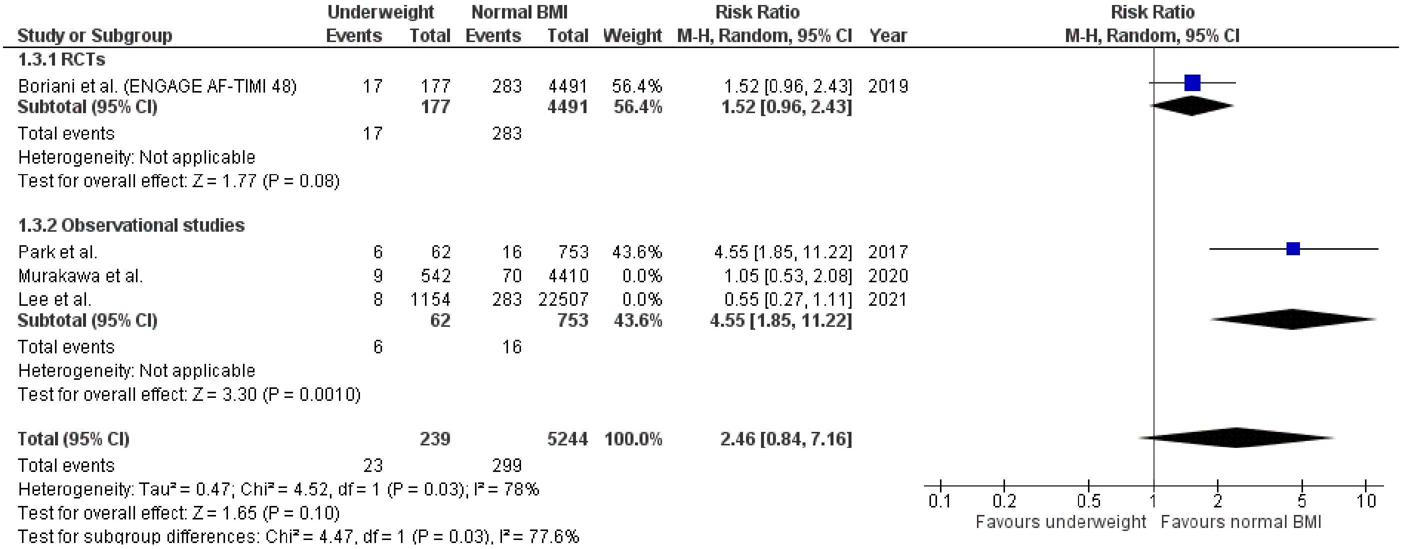

eFigure 6: Forest plot of the risk of major bleeding for underweight (BMI <18.5 kg/m²) versus normal BMI (18.5-<25 kg/m²) AF patients receiving anticoagulation, after one-by-one exclusion of the results of the observational studies.

AF: atrial fibrillation; BMI: body mass index; CI: confidence interval; ENGAGE AF-TIMI 48: the Effective Anticoagulation with Factor Xa Next Generation in Atrial Fibrillation–Thrombolysis in Myocardial Infarction 48 trial; M-H: Mantel-Haenszel (statistical method); RCT: randomized controlled trial.

eFigure 7: Assessment of publication bias

A1)

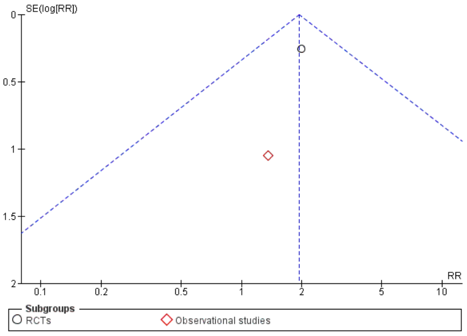

B1)

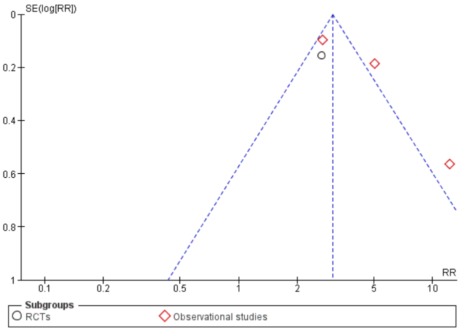

C1)

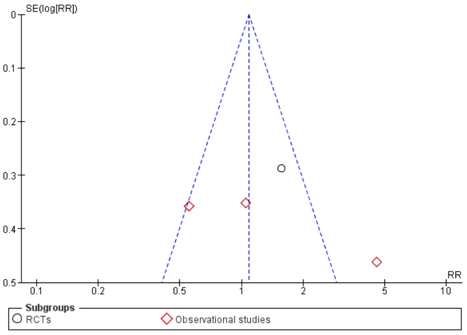

D1)

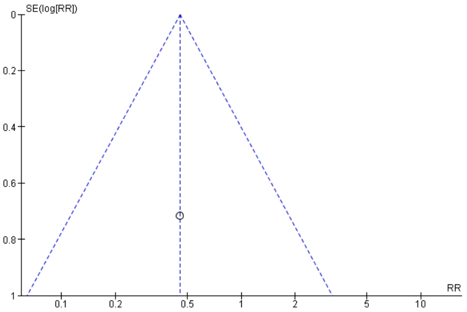

A2)

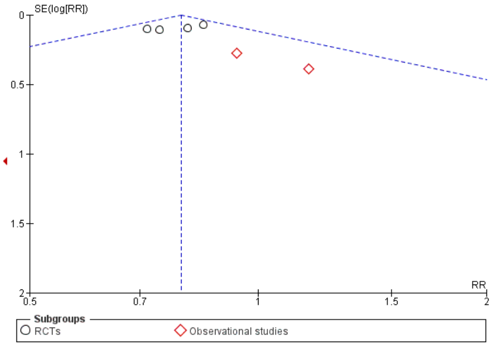
SE(log[RR])
0
0.5
1
1.5
2
0.5
0.7
1
1.5
2
RR
Subgroups
RCTs
Observational studies

B2)

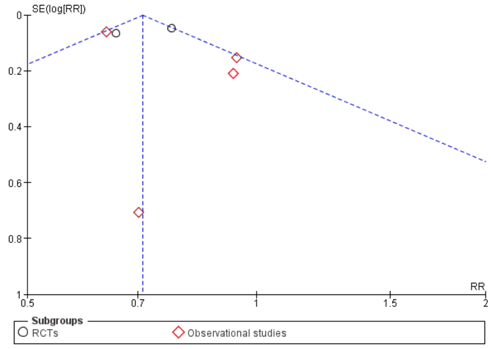
SE(log[RR])
0
0.2
0.4
0.6
0.8
1
0.5
0.7
1
1.5
2
RR
Subgroups
RCTs
Observational studies

C2)

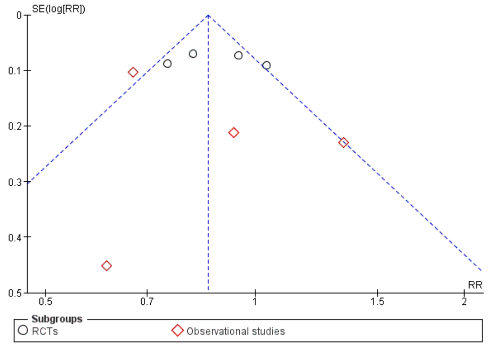
SE(log[RR])
0
0.1
0.2
0.3
0.4
0.5
0.5
0.7
1
1.5
2
RR
Subgroups
RCTs
Observational studies

D2)

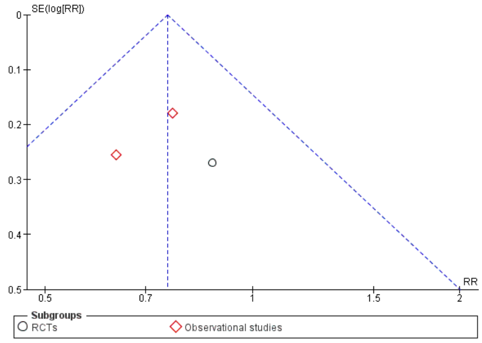
SE(log[RR])
0
0.1
0.2
0.3
0.4
0.5
0.5
0.7
1
1.5
2
RR
Subgroups
RCTs
Observational studies

A3)

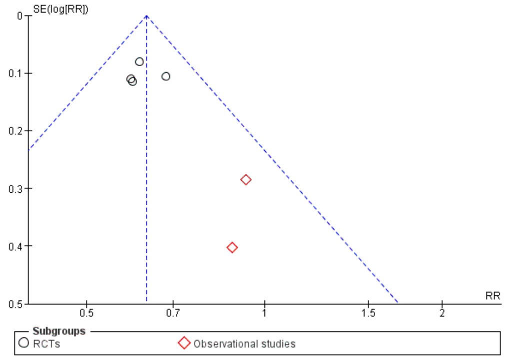
SE(log[RR])
0
0.1
0.2
0.3
0.4
0.5
0.5
0.7
1
1.5
2
RR
Subgroups
RCTs
Observational studies

B3)

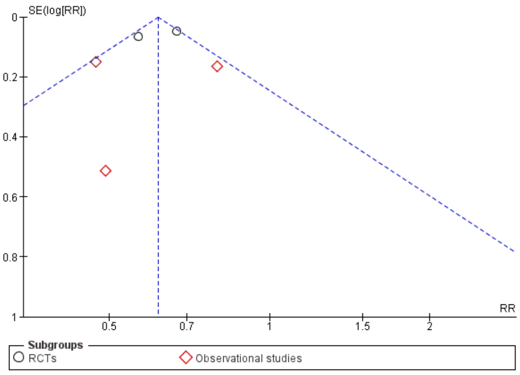
SE(log[RR])
0
0.2
0.4
0.6
0.8
1
0.5
0.7
1
1.5
2
RR
Subgroups
RCTs
Observational studies

C3)

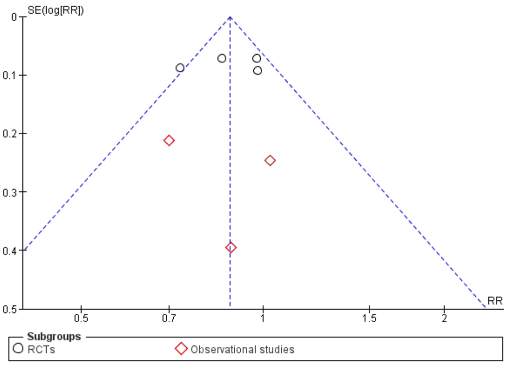
SE(log[RR])
0
0.1
0.2
0.3
0.4
0.5
0.5
0.7
1
1.5
2
RR
Subgroups
RCTs
Observational studies

D3)

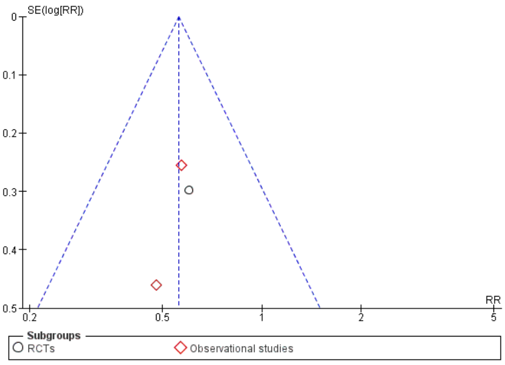
SE(log[RR])
0
0.1
0.2
0.3
0.4
0.5
0.2
0.5
1
2
5
RR
Subgroups
RCTs
Observational studies

A4)

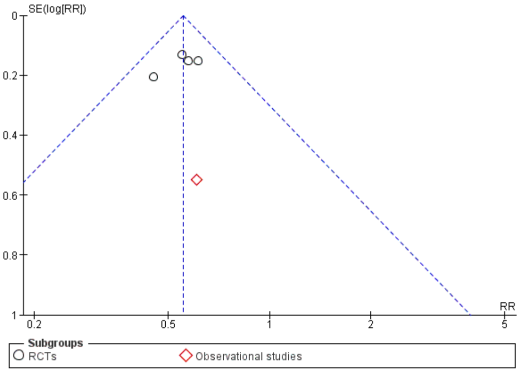
SE(log[RR])
0
0.2
0.4
0.6
0.8
1
0.2
0.5
1
2
5
RR
Subgroups
RCTs
Observational studies

B4)

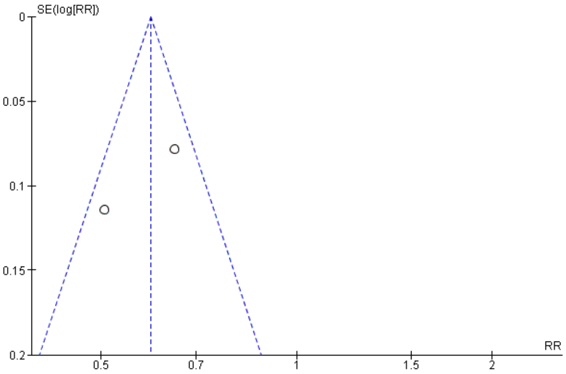
SE(log[RR])
0
0.05
0.1
0.15
0.2
0.5
0.7
1
1.5
2
RR

C4)

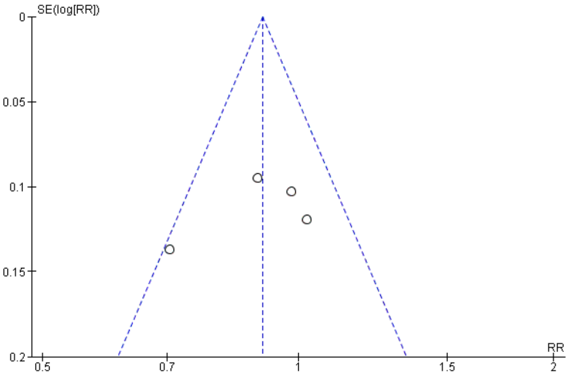
SE(log[RR])
0
0.05
0.1
0.15
0.2
0.5
0.7
1
1.5
2
RR

D4)

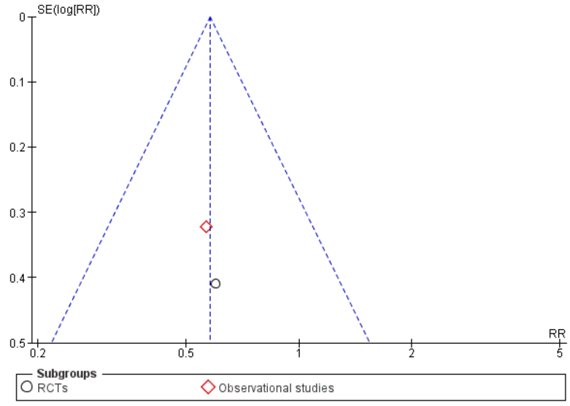
SE(log[RR])
0
0.1
0.2
0.3
0.4
0.5
0.2
0.5
1
2
5
RR
Subgroups
RCTs
Observational studies

A5)

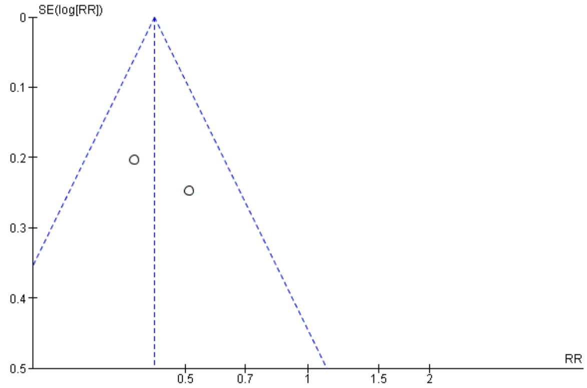
SE(log[RR])
0
0.1
0.2
0.3
0.4
0.5
0.5
0.7
1
1.5
2
RR

B5)

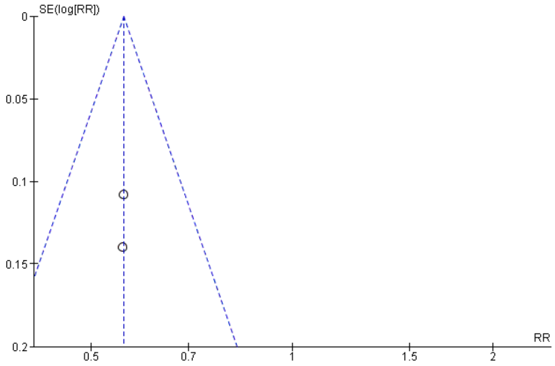

C5)

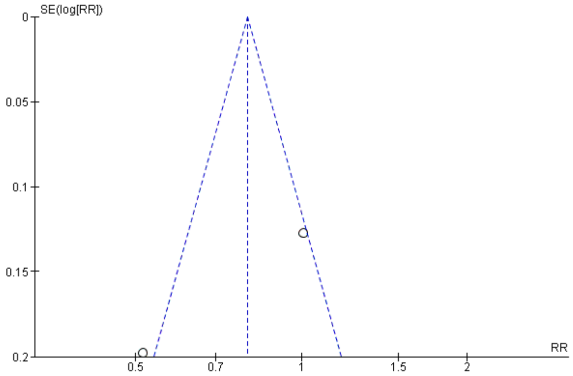

D5)

No data

eFigure 7: Funnel plot for assessment of potential publication bias for the risk of **A)** stroke or systemic embolism, **B)** all-cause mortality, **C)** major bleeding, and **D)** intracranial bleeding in underweight **(A-D1)**, overweight **(A-D2)**, obese **(A-D3)**, class II obese **(A-D4)** and morbidly obese **(A-D5)** versus normal BMI atrial fibrillation patients.

References

1. Connolly SJ, Ezekowitz MD, Yusuf S, et al. Dabigatran versus warfarin in patients with atrial fibrillation. N Engl J Med. 2009;361(12):1139-51.
2. Eikelboom JW, Wallentin L, Connolly SJ, et al. Risk of bleeding with 2 doses of dabigatran compared with warfarin in older and younger patients with atrial fibrillation: an analysis of the randomized evaluation of long-term anticoagulant therapy (RE-LY) trial. Circulation. 2011;123(21):2363-72.
3. Boehringer Ingelheim. Pradaxa (dabigatran), U.S. Food and Drug Administration Highlights of Prescribing Information. https://www.accessdata.fda.gov/drugsatfda_docs/label/2015/022512s027lbl.pdf. Accessed 10 July 2020.
4. Hohnloser SH, Fudim M, Alexander JH, et al. Efficacy and Safety of Apixaban Versus Warfarin in Patients With Atrial Fibrillation and Extremes in Body Weight. Circulation. 2019;139(20):2292-300.
5. Xarelto (rivaroxaban). U.S. Food and Drug Administration Highlights of Prescribing Information. https://www.accessdata.fda.gov/drugsatfda_docs/label/2018/022406s028lbl.pdf. Accessed 10 July 2020.
6. Savaysa (edoxaban). U.S. Food and Drug Administration Highlights of Prescribing Information. https://www.accessdata.fda.gov/drugsatfda_docs/label/2015/206316lbl.pdf. Accessed 10 July 2020.
7. Boriani G, Ruff CT, Kuder JF, et al. Edoxaban versus Warfarin in Patients with Atrial Fibrillation at the Extremes of Body Weight: An Analysis from the ENGAGE AF-TIMI 48 Trial. Thromb Haemost. 2020.
8. Boonyawat K, Caron F, Li A, et al. Association of body weight with efficacy and safety outcomes in phase III randomized controlled trials of direct oral anticoagulants: a systematic review and meta-analysis. Journal of thrombosis and haemostasis : JTH. 2017;15(7):1322-33.
9. Hamatani Y, Ogawa H, Uozumi R, et al. Low Body Weight Is Associated With the Incidence of Stroke in Atrial Fibrillation Patients - Insight From the Fushimi AF Registry. Circulation journal : official journal of the Japanese Circulation Society. 2015;79(5):1009-17.
10. Yasuda K, Fukuda S, Nakamura M, et al. Predictors of Cardioembolic Stroke in Japanese Patients with Atrial Fibrillation in the Fushimi AF Registry. Cerebrovascular diseases extra. 2018;8(2):50-9.
11. Kuronuma K, Okumura Y, Yokoyama K, et al. Different determinants of vascular and nonvascular deaths in patients with atrial fibrillation: A SAKURA AF Registry substudy. Journal of cardiology. 2019;73(3):210-7.
12. Sandhu RK, Ezekowitz J, Andersson U, et al. The 'obesity paradox' in atrial fibrillation: observations from the ARISTOTLE (Apixaban for Reduction in Stroke and Other Thromboembolic Events in Atrial Fibrillation) trial. Eur Heart J. 2016;37(38):2869-78.
13. Balla SR, Cyr DD, Lokhnygina Y, et al. Relation of Risk of Stroke in Patients With Atrial Fibrillation to Body Mass Index (from Patients Treated With Rivaroxaban and Warfarin in the Rivaroxaban Once Daily Oral Direct Factor Xa Inhibition Compared with Vitamin K Antagonism for Prevention of Stroke and Embolism Trial in Atrial Fibrillation Trial). The American journal of cardiology. 2017;119(12):1989-96.
14. Boriani G, Ruff CT, Kuder JF, et al. Relationship between body mass index and outcomes in patients with atrial fibrillation treated with edoxaban or warfarin in the ENGAGE AF-TIMI 48 trial. Eur Heart J. 2019;40(19):1541-50.
15. Boehringer Ingelheim Pharmaceuticals. FDA Advisory Committee Briefing Document, Dabigatran Etexilate Mesylate Capsules, for the September 20, 2010 Meeting of the Cardiovascular and Renal Drugs Advisory Committee. https://wayback.archive-it.org/7993/20170405212218/https://www.fda.gov/downloads/AdvisoryCommittees/CommitteesMeetingMaterials/Drugs/CardiovascularandRenalDrugsAdvisoryCommittee/UCM247244.pdf. Accessed 1 September 2020.
16. Proietti M, Guiducci E, Cheli P, Lip GY. Is There an Obesity Paradox for Outcomes in Atrial Fibrillation? A Systematic Review and Meta-Analysis of Non-Vitamin K Antagonist Oral Anticoagulant Trials. Stroke. 2017;48(4):857-66.
17. Zhou Y, Ma J, Zhu W. Efficacy and Safety of Direct Oral Anticoagulants Versus Warfarin in Patients with Atrial Fibrillation Across BMI Categories: A Systematic Review and Meta-Analysis. American Journal of Cardiovascular Drugs. 2020;20(1):51-60.
18. Badheka AO, Rathod A, Kizilbash MA, et al. Influence of obesity on outcomes in atrial fibrillation: yet another obesity paradox. The American journal of medicine. 2010;123(7):646-51.
19. Overvad TF, Rasmussen LH, Skjøth F, et al. Body mass index and adverse events in patients with incident atrial fibrillation. The American journal of medicine. 2013;126(7):640.e9-.e6.4E17.
20. Hamatani Y, Yamashita Y, Esato M, et al. Predictors for Stroke and Death in Non-Anticoagulated Asian Patients with Atrial Fibrillation: The Fushimi AF Registry. PloS one. 2015;10(11):e0142394.
21. Pandey A, Gersh BJ, McGuire DK, et al. Association of Body Mass Index With Care and Outcomes in Patients With Atrial Fibrillation: Results From the ORBIT-AF Registry. JACC Clinical electrophysiology. 2016;2(3):355-63.

22. Inoue H, Kodani E, Atarashi H, et al. Impact of Body Mass Index on the Prognosis of Japanese Patients With Non-Valvular Atrial Fibrillation. The American journal of cardiology. 2016;118(2):215-21.
23. Senoo K, Lip GY. Body Mass Index and Adverse Outcomes in Elderly Patients With Atrial Fibrillation: The AMADEUS Trial. Stroke. 2016;47(2):523-6.
24. Lee CH, Lin TY, Chang SH, et al. Body mass index is an independent predictor of major bleeding in non-valvular atrial fibrillation patients taking dabigatran. Int J Cardiol. 2017;228:771-8.
25. Wang H, Wang HJ, Chen YD, et al. Prognostic factors of clinical endpoints in elderly patients with atrial fibrillation during a 2-year follow-up in China: An observational cohort study. Medicine (United States). 2017;96(33).
26. Park CS, Choi EK, Kim HM, et al. Increased risk of major bleeding in underweight patients with atrial fibrillation who were prescribed non-vitamin K antagonist oral anticoagulants. Heart Rhythm. 2017;14(4):501-7.
27. Shinohara M, Fujino T, Yao S, et al. Assessment of the bleeding risk of anticoagulant treatment in non-severe frail octogenarians with atrial fibrillation. Journal of cardiology. 2019;73(1):7-13.
28. Wang L, Du X, Dong JZ, et al. Body mass index and all-cause mortality in patients with atrial fibrillation: insights from the China atrial fibrillation registry study. Clinical research in cardiology : official journal of the German Cardiac Society. 2019;108(12):1371-80.
29. Netley J, Howard K, Wilson W. Effects of body mass index on the safety and effectiveness of direct oral anticoagulants: a retrospective review. Journal of Thrombosis and Thrombolysis. 2019;48(3):359-65.
30. Murakawa Y, Ikeda T, Ogawa S, et al. Impact of body mass index on real-world outcomes of rivaroxaban treatment in Japanese patients with non-valvular atrial fibrillation. Heart and vessels. 2020.
31. Bertomeu-Gonzalez V, Moreno-Arribas J, Esteve-Pastor MA, et al. Association of Body Mass Index With Clinical Outcomes in Patients With Atrial Fibrillation: A Report From the FANTASIIA Registry. J Am Heart Assoc. 2020;9(1):e013789.
32. Lucijanic M, Jurin I, Jurin H, et al. Patients with higher body mass index treated with direct / novel oral anticoagulants (DOAC / NOAC) for atrial fibrillation experience worse clinical outcomes. Int J Cardiol. 2020;301:90-5.
33. Li MH, Hu LH, Xiong YR, et al. Association between body mass index and the risk of bleeding in elderly patients with non-valvular atrial fibrillation taking dabigatran: a cohort study. Journal of geriatric cardiology : JGC. 2020;17(4):193-201.
34. Okumura K, Tomita H, Nakai M, et al. Risk Factors Associated With Ischemic Stroke in Japanese Patients With Nonvalvular Atrial Fibrillation. JAMA network open. 2020;3(4):e202881.
35. Patti G, Pecen L, Manu MC, et al. Thromboembolic and bleeding risk in obese patients with atrial fibrillation according to different anticoagulation strategies. Int J Cardiol. 2020;318:67-73.
36. Kaplan RM, Tanaka Y, Passman RS, et al. Efficacy and Safety of Direct Oral Anticoagulants for Atrial Fibrillation Across Body Mass Index Categories. J Am Heart Assoc. 2020;9(24):e017383.
37. Lee SR, Choi EK, Jung JH, et al. Body Mass Index and Clinical Outcomes in Asian Patients With Atrial Fibrillation Receiving Oral Anticoagulation. Stroke. 2021:STROKEAHA120030356.
38. Kmet L, Lee R, Cook L. The quality assessment tool 'QUALSYST' from the "Standard Quality Assessment Criteria for Evaluating Primary Research Papers from a Variety of Fields". 2004. https://www.ihe.ca/advanced-search/standard-quality-assessment-criteria-for-evaluating-primary-research-papers-from-a-variety-of-fields. Accessed 1 August 2020.
